# A systematic review of sport-based adolescent mental health awareness programmes

**DOI:** 10.1371/journal.pone.0315315

**Published:** 2025-03-27

**Authors:** Nora Sullivan, Gavin Breslin, Marian McLaughlin, Stephen Shannon, Gerard Leavey, Martin Dempster

**Affiliations:** 1 School of Psychology, Queens University Belfast, Belfast, Northern Ireland; 2 Bamford Centre for Mental Health and Wellbeing, School of Psychology, Ulster University, Coleraine, Northern Ireland; 3 School of Psychology, Ulster University, Coleraine, Northern Ireland; 4 Sport and Exercise Sciences Research Institute, Ulster University, Belfast, Northern Ireland; Rehabilitative Hospital Affiliated to Fujian University of Traditional Chinese Medicine: Fujian University of Traditional Chinese Medicine Affiliated Rehabilitative Hospital, CHINA

## Abstract

**Background:**

Adolescent mental illness is of increasing concern, with a high prevalence in many parts of the world. Early engagement, detection and receiving support are warranted to reduce the severity of symptoms. Increasing mental health literacy (MHL) through sport to adolescents is one way of engaging young people and signposting them to services. The aim of this systematic review was to identify the effect of interventions, risk of bias, and theoretical application in sport-based adolescent mental health awareness programmes.

**Methods:**

Six electronic databases (MEDLINE Ovid, PsycINFO, Scopus, CINAHL, SPORTDiscus, Cochrane) were searched from 2012 to September 2022 (updated January 2024). Inclusion criteria stated the sample had to include adolescents aged 11–17 years, include mental health outcomes, and a sport component.

**Results:**

Six studies met the inclusion criteria. Sample size ranged from nine to 816 participants. Four distinct sport-based programmes were evaluated, with three interventions applying a psychological behaviour change theory. The results of the interventions indicated positive effects on several indices of MHL, such as increased knowledge of mental health, depression and anxiety literacy and the recognition of disorders, increased resilience and intentions to provide help. It was found that addressing stigma remains a challenge. The interventions produced the most significant effects for those who scored lower at baseline measures and the younger cohorts.

**Conclusions:**

Sport is useful for the engagement and dissemination of mental health awareness information to adolescents. Given the limited number of psychological theory informed interventions, there is a need for further interventions that explicitly adopt behaviour change theories and improve the quality of research design for these interventions. The findings from this review will be of interest to health promotion and public health practitioners and those designing mental health awareness programmes for adolescents.

**Systematic review registration:**

PROSPERO CRD42022312260

## Introduction

According to the United Nations International Children’s Emergency Fund (UNICEF) “The State of the World’s Children” 2021 report, estimated an 86 million adolescents aged 15-19 years, and 80 million adolescents aged 10-14 years live with a mental disorder [1]. The World Health Organisation (WHO) have stated the negative effects of inadequate intervention to adolescent mental health conditions results in lasting problems into adulthood [2]. Studies have shown that the highest prevalence of mental health disorders occur during adolescence and young adulthood, compared to any other stage of the lifespan [3], yet adolescents are recognised as displaying lower levels of help-seeking to professionals in comparison to adults [4]. Mental health literacy (MHL) is defined as “knowledge and beliefs about mental disorders which aid their recognition, management or prevention” [5]. This concept encompasses multiple elements including: (a) the ability to recognise specific disorders or types of psychological distress; (b) knowledge and beliefs about risk factors and causes; (c) knowledge and beliefs about self-help interventions; (d) knowledge and beliefs about professional help available; (e) attitudes which facilitate recognition and appropriate help-seeking; and (f) knowledge of how to seek mental health information [6]. By increasing adolescents’ MHL, there is a potential to increase help seeking and for a reduction of serious mental illness [7]. Poor MHL and stigma have been identified as barriers for young people to seek help [8]. A more recent study also identified stigma of mental illness as being a significant barrier to help-seeking [9], stating that the absence of basic knowledge on mental health results in misunderstandings and the development of enduring negative stigmas. By deterring help seeking, treatment-use and recovery, the stigmas associated with mental illness can worsen psychological symptoms and further compromise an individual’s mental health [10,11]. Therefore, early engagement and the education of young people on MHL is important in prevention and facilitating early treatment.

A challenge of increasing MHL is securing the engagement of young people in mental health awareness training. Renewed efforts are needed to increase MHL and educate adolescents on the help available. By integrating mental health awareness and self-care messaging into leisure activities which youth typically enjoy and participate in, there is potential for the programme to be attractive for uptake and possibly increase effectiveness. Implementation science offers a range of theories, models, and frameworks aimed at enhancing the dissemination of evidence-based interventions [12]. Implementation science includes customizing innovations to fit local contexts, gaining a deeper insight into the implementation environment, and evaluating the implementation procedure [13]. A comprehensive understanding of the context is instrumental in aligning the innovation and implementation strategies, ultimately boosting the feasibility, acceptability, and long-term viability of the intervention [14].

Sport and the community setting can be a suitable environment for increasing MHL and reducing stigma, as it has the ability to engage young people in mental health dialogue, particularly among those who are typically disengaged by the use of general public health messaging [15]. Sport has the ability to engage young people in mental health promotion due to its cultural appeal, its emphasis on fun and enjoyment, and establishment of social networks [15]. In recent years there has been an emergence in sport based mental health awareness initiatives that seek to tackle the rising mental health problems, focusing on concepts associated with MHL, areas including de-stigmatising attitudes, improving knowledge of mental health, and establishing paths for help-provision. A study by Swann et al., [16] found that adolescent males perceived sport to be an engaging way for supporting mental health in young people, and therefore offers potential for future programme development and intervention. A recent systematic review and meta-analysis of mental health awareness programmes in sport showed programme effectiveness in increasing MHL, including having significant effects on knowledge of mental health, stigmatising attitudes and help-provision [17]. This review was based on studies from 2004-2020, and it included interventions involving athletes, parents and coaches. Other systematic reviews have been conducted on sport and mental health, mostly focused on elite athletes, with similar messages that mental health awareness can be incorporated into sport settings [18–21]. While these reviews offer valuable information, there has yet to be a systematic review focused directly on adolescent MHL promotion through sport.

Existing reviews have also highlighted some limitations including studies typically being of low methodological quality [22,23], a lack of evidence-based approaches, and interventions of limited efficacy [24]. Also, within many reviews programme content or evaluation has not been underpinned by psychological theory of behaviour change. The inclusion of a psychological behaviour change theory can offer valuable data on the aspects of interventions responsible for, and likely to facilitate behaviour change [25]. Utilizing a validated theory may significantly increase the likelihood of producing successful interventions and positive outcomes, as these theories play a vital role in understanding the underlying factors that influence human behaviour. Theories aim to provide explanations and predictions regarding when, how, and why behaviour (change) occurs or does not occur [26]. They propose potential action mechanisms and variables that can impact change within various causal pathways. For theory-based interventions to be effective, their active components should target relevant mechanisms of action and be implemented at all stages of the design, delivery, and subsequent evaluation of an intervention to ensure a higher probability of the intervention being effective and reliable in changing behaviours [26]. The existing interventions that do mention a theory, provide limited detail of how the theory has been used and implemented in the intervention.

In response to the lack of a systematic review of sport based mental health awareness in adolescents, the present systematic review is required. The primary objective of this review is to examine programme effectiveness, research design, risk of bias and methodological strength, and theoretical application in the design and evaluation of sport-based adolescent mental health awareness programmes. The review aimed to answer the following research questions: 1. Are sport-based programmes effective in increasing adolescent mental health awareness? 2. What are the content and research design of existing interventions? and 3, is a psychological theory of behaviour change included in the design and implementation of the programmes, and does the theory feature in their subsequent evaluation?

## Methods

### Protocol

This review was registered on the International Prospective Register of Systematic Reviews (PROSPERO Registration Number: CRD42022312260). The Preferred Reporting Items for Systematic Reviews and Meta-Analyses (PRISMA) guidelines [27] were followed. A PRISMA checklist is included as supplementary file one.

### Eligibility criteria

This review will focus solely on non-elite adolescent participants and will examine peer reviewed articles published from 2012- September 29^th^, 2022 (search updates January 30^th^, 2024). Eligibility criteria was structured around the Population, Intervention, Control, Outcome (PICO) framework [28].

#### Population.

The population included adolescents, aged 11–17 years old, all genders, and those with disabilities.

#### Intervention.

Interventions which addressed mental health awareness were included. This included programmes that aimed to improve awareness of mental health literacy such as the recognition and management of psychological or emotional problems, or interventions tailored to focus on a specific mental health disorder (e.g., depression, anxiety, substance misuse). To be eligible, the intervention was required to include a sport component. To meet this requirement, the intervention had to be delivered to a sporting organization or involve sport-based activities for the promotion of mental health messaging. Specifically, interventions which focused on burnout or psychological performance profiling, or non-mental health related themes were excluded from the review. The mode of intervention delivery was to individuals, groups or online via the worldwide web.

#### Control.

Studies were included if they contained control or comparison groups, within-group comparison study designs. Qualitative studies were also included.

#### Outcomes.

Studies had to include at least one outcome measure relating to mental health awareness, MHL, or self-management of emotional and psychological problems. This could include attitudes towards mental health, knowledge of mental health (e.g., disorder and symptom recognition) or intention to or actual help-seeking behaviour.

### Information sources and search strategy

Six electronic databases including MEDLINE Ovid, PsycINFO, Scopus, CINAHL, SPORTDiscus, and Cochrane were searched. Each database was searched from 2012 up until September 29^th^, 2022. Key words were used for the search strategy, with truncation and Mesh terms to ensure maximum results from each database (See Table 1). The search strategy was developed by the authors alongside the institution’s librarian.

**Table 1:** An example of search terms used in the PsychInfo database.

*Search Concept-*	*Alternatives-*
Mental Health Program *	Mental health intervention
	Mental health awareness
	Mental health literacy
	Mental wellbeing
	Mental health disorder *
	Mental illness
	Depress *
	Anxiety
	Help-seeking behaviour *
	Mental health symptom recognition
Adolescent *	Young people
	Teen *
	Youth
	Young adult *
	Young male *
	Young female *
	Child *
Sport *	Exercis *
	Physical activit *
	Fitness

### Study selection

All references retrieved from the electronic databases were imported into the web-based systematic review software, Covidence [29]. Following the removal of duplicates, all titles and abstracts were screened by the lead author to assess eligibility (NS). A screening of 15% of the articles was completed by GB, MM, and SS. There was a high level of agreement between reviewers, and any conflicts (<2%) were discussed by authors until a consensus was reached on article inclusion or exclusion. Articles which met the eligibility criterion were sourced and full-text articles were reviewed by NS independently against the inclusion and exclusion criteria and inclusion confirmed by the other reviewers.

### Search terms. Data extraction

A data extraction table was developed (Table 1 in S1 File). All included studies underwent a narrative analysis with the information extracted summarised and presented in the table. The study aim, design, participant demographics (including the number of participants, age range and gender), duration of intervention, use of behaviour change theory, outcome measures and results were extracted. Unrelated outcomes such as physical health and economic variables (e.g., intervention cost effectiveness) were excluded.

### Methodological quality assessment

The Modified Methodological Quality Checklist [30] was used to assess the methodological quality of the quantitative studies. The checklist has a criterion validity score of *r* =  0.90, an internal consistency of 0.89, and a test-retest reliability of *r* =  0.88 [31]. The checklist consists of 27 items across 5 domains (reporting, external validity, internal validity, bias, and statistical power). The checklist was used to assess the quality of both randomised and non-randomised controlled studies. Randomised studies can score a maximum of 28, while non-randomised had a maximum of 25. All items were scored 0-1, except for item 5 which was scored 0-2. If the answer is no or the authors are unable to determine the answer, a score of 0 was given. If yes, it is scored 1. For item 5, a score of 2 means yes, 1 means partially, and 0 means no. Studies were rated as: excellent with a score of >  21; moderate if scored 14–20; limited with a score of 7–13; and poor if scored 7 or less [32–34]. NS and MM completed the quality assessments.

The Critical Appraisal Skills Programme (CASP) is a generic tool for appraising the strengths and limitations of any qualitative research methodology [35]. The CASP tool is endorsed by the Cochrane Qualitative and Implementation Methods Group [36]. When using the tool, you begin by selecting the checklist which aligns with the study you are examining, from a list of eight different study types. Each checklist consists of ten questions, which are each answered yes, no, or can’t tell. The questions each focus on a different methodological aspect of a qualitative study, and are separated into three sections; Section A, are the results of the study valid?; Section B, what are the results?; Section C, will the results help locally? The CASP tool prompts the researcher to evaluate whether the chosen research methods were suitable and if the results are effectively showcased and are of significance.

The Mixed Methods Appraisal Tool (MMAT) [37] is a critical appraisal tool that is designed for the appraisal stage of systematic mixed studies reviews (i.e., reviews that include qualitative, quantitative and mixed methods studies). The MMAT is used to assess the methodological quality of five categories to studies: qualitative research, randomized controlled trials, non-randomized studies, quantitative descriptive studies, and mixed methods studies. MMAT is separated into two parts. Part one contains two screening questions, which help identify whether this tool is appropriate for the study or not. If the answer to the two screening questions is both no, then it would be suggested that a different critical appraisal tool is used. For part two of the MMAT, each included study must be placed into one of the five identified categories. Upon choosing the relevant category, the criteria are evaluated through the response to five questions, which can be answered as “yes,” “no,” or “uncertain.” It is not advisable to calculate an overall score by aggregating individual criterion ratings. Instead, it is recommended to provide a more detailed breakdown of the ratings for each criterion to improve comprehension of the quality of the studies included.

## Results

### Search results

**Fig 1:**
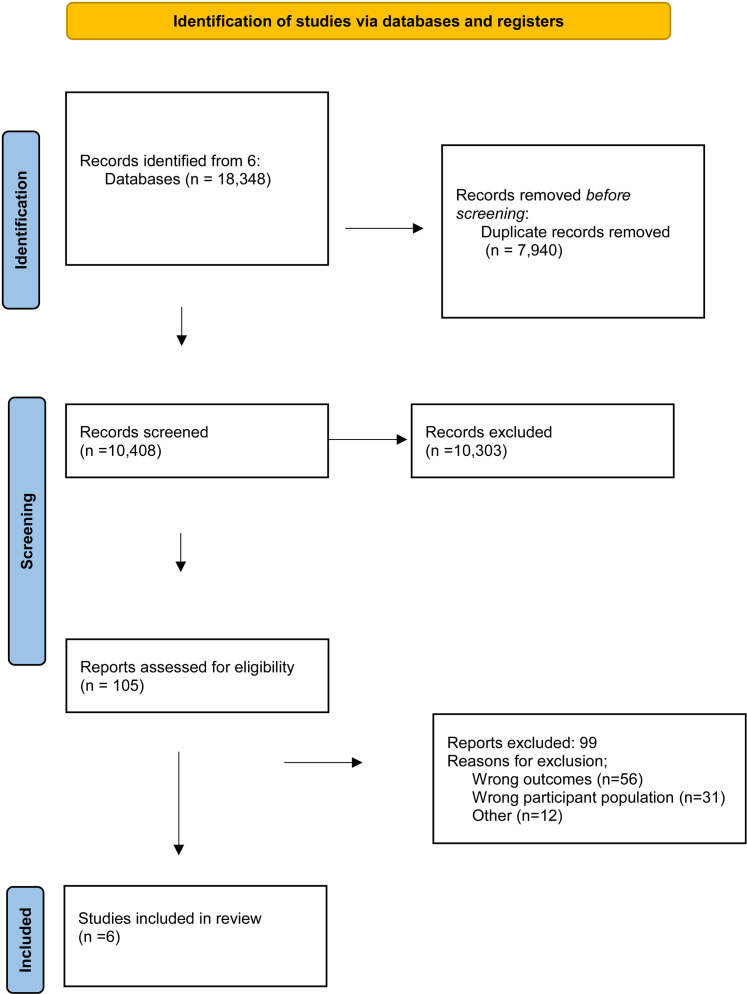
PRSIMA flow diagram of the study selection process.

The search yielded 18,348 articles. Of these, 7,940 were identified as duplicates and removed. One researcher (NS) completed the title and abstract screening for all 10,408 articles, with three researchers (GB, MM, SS) screening a combined total of 15% of the articles as second screeners. A total of 105 articles were identified as being potentially relevant and considered for full text screening. After full text review, which was completed independently by NS against the inclusion and exclusion criteria, 99 articles were excluded. Exclusion was due to one or more of the following reasons: incorrect population, incorrect outcomes, article type (e.g., conference abstract, study protocol, dissertation chapter), or study design. A total of six studies met the full inclusion criteria and were included in the final analysis. Fig 1 illustrates the PRISMA flow diagram detailing the study selection process.

### Description of studies

The study characteristics for each of the six included articles are outlined in Table 2. These included: author/year of study publication; study design; duration of intervention; the inclusion of a behaviour change theory in the programme design or evaluation; and sample characteristics.

**Table 2:** Author, year of publication, intervention name, study design, duration, psychological theory and participant sample characteristics of included studies.

Author, Year of Publication, Intervention	Study design; duration	Psychological Behaviour Change Theory	Participant Sample characteristics
Liddle et al. 2021 [38] Ahead of the Game	Clustered RCT; One 45-minute session	Integrated Behaviour Change Theory Self-Determination Theory	102 males (mean age 14.30) Intervention = 59 Control = 63
McKenzie et al. 2021 [39] Waves of Wellness	Mixed-methods Exploratory Research; 8 weeks of 2-hour sessions (45 minutes mental health related discussion, 60 minutes sea-surfing)	Ecological Dynamics Perspective	9 youth (mean age 14.9) 8 females 1 male
Moore et al. 2021 [40] Wellbeing Warriors	Experimental research design using a clustered RCT; 10 weeks, one 50/60-minute session per week	Not reported	Schools = 5; 283 youth (mean age 12.76) Intervention = 142 Control = 141
Patafio et al. 2021 [41] Read the Play	Repeated measures experimental-control design; single 1 hour session	Not reported	Sport clubs = 12, 330 youth (mean age 13.73) Experimental = 10 sport clubs (272) Control = 2 sport clubs (58)
Vella et al. 2021 [15] Ahead of the Game	Non-randomised control trial; 2 45-minute face-to-face sessions and 6 internet modules lasting 15 minutes each)	Socio-Ecological Approach	816 males Intervention = 350 males (mean age 14.53) Control = 466 males (mean age 14.66)
Wynters et al. 2021 [42] Ahead of the Game	Retrospective qualitative design; 45 minutes in person session	Not reported	33 males, 6 focus groups

### Methodological quality assessment

The quality of each of the four quantitative studies included in this review were assessed using a modified version of the Downs and Black checklist [30]. One qualitative study [42] was assessed using the MMAT questions [37], while the mixed methods study [39] was assessed using the Critical Appraisal Skills Programme [35].

### Study design

All articles were published in 2021 and were conducted in Australia. Four of the six studies included a control or comparison group [15,38,40,41], while two did not [39,42]. The study sample size ranged greatly from nine [39] to 816 participants [15]. Three studies included both male and female participants in the intervention [39–41], while three studies [15,38,42] only included males.

Three articles evaluated the Ahead of the Game intervention, they all assessed the outcomes of the programme at different time points. Vella et al. [15] used a non-randomised control trial with baseline measures being recorded and follow-up measures taken after the programme completion (6-8 weeks after baseline). Liddle et al. [38] adopted a cluster-randomised control trial and took measures at three time points; at baseline (2 weeks prior to intervention), immediately post intervention, and at one month follow-up. Wynters et al. [42] took a retrospective qualitative approach conducting focus groups with participants after the soccer season completed.

Patafio et al. [41] employed a repeated measures experimental-control design using a convenience sample. McKenzie et al. [39] adopted a mixed-methods exploratory research design, with quantitative measures collected at four time points; baseline (0 weeks), programme mid-point (four weeks), programme end (8 weeks), and follow-up (12 weeks). Interviews were then conducted at the follow up in week 12. Finally, Moore et al. [40] undertook an experimental randomised control trial with participant measurements being conducted at baseline, post-intervention, and follow-up (12 weeks post intervention).

### Theoretical approach

Of the six articles included, only three authors explicitly reported a health behaviour change theory. The Integrated Behaviour Change Model (IBC) [43,44] combines the core factors of the Theory of Planned Behaviour [25] with Self-determination Theory [45]. The IBC was integrated by Liddle et al. [38] in the Ahead of the Game intervention. According to the IBC, individuals align their beliefs and intentions with their motives in order to pursue a behaviour in the future. The intervention was designed in line with this theory, by targeting subjective norms, perceived behavioural control, and attitudes. Attitudes towards mental illness were addressed by dispelling common misconceptions about mental health, facilitating interactions with individuals who have first-hand experience with mental illness, highlighting instances of elite athletes who have dealt with mental health challenges, and emphasizing the advantages of timely and supportive interventions. Subjective norms were targeted by emphasizing the potential negative consequences of unnoticed mental health issues and the significance of being a supportive team member, recognizing their crucial role in mutual support. Perceived behavioural control is addressed through skill practice via role-playing exercises and by actively addressing and minimizing hindrance from barriers by discussing barriers.

The Waves of Wellness intervention adopted the Ecological Dynamics Perspective [46] which combines Dynamic Systems Theory [47] and a Constraint-led Perspective [48]. The constraints-led perspective alters the outlook of practitioners on how to deal with individual differences, structure practice for optimal learning, provide verbal instructions and feedback, and guide learners’ focus while observing skills [48]. The ecological dynamics perspective takes into account the interactions of the individual, environment, and task to explain the formation of processes of action, perception and cognition [49]. This viewpoint suggests that the psychological advantages of nature, including engaging in action and adventure sports, could be a result of the dynamic interplay between the individual, their distinct attributes and life experiences, the sport (i.e., surfing), and the surrounding environment. Consistent with the ecological dynamics perspective, existing research suggests that surf therapy yields psychological benefits, allowing for a heightened sensory experience, building self-efficacy, promoting a sense of mastery, and fostering a culture of acceptance and connection [50]. McKenzie et al., [39] used the ecological dynamics perspective to inform the surf-based intervention, with a focus on resilience, self-esteem, social-connectedness, and negative psychological symptoms.

Finally, the Socio-Ecological Approach to health promotion [51] was adopted by Vella et al., [15]. The Socio-Ecological Approach highlights the importance of health promotion targeting not just individual behaviours, but also the various factors at multiple levels that impact those behaviours. By adopting the Socio-Ecological Approach, the emphasis lies on understanding the interconnectedness between individuals and their social, physical, and policy environments, which all play a role in shaping specific behaviours [52]. The Socio- Ecological Approach was integrated by Vella et al., [15] when designing the intervention, which led to the creation of a multilevel programme, intervening with not only the athlete, but the coaches and parents as well.

### Delivery mode

All the interventions included at least one in person session, with some of the interventions containing online components. All in person sessions were group based. The person delivering the sessions differed in each intervention. Waves of Wellness [39] was delivered by mental health clinicians who were qualified surf instructors. This intervention was directed at at-risk youth, who were recruited through Waves of Wellness partner mental health organisations. Additionally, participants could be referred by their doctor, case manager, or complete a self-referral. All the sessions took place on a beach. Wellbeing Warriors [40] was delivered by a registered psychologist who had a minimum of 6 years’ experience as a school psychologist, and taekwondo instructor with a minimum of 5 years’ experience. This intervention took place in secondary school settings. Read the Play [41] was delivered by a mental health professional and was delivered in amateur sport settings. This intervention was designed to target those aged under 15 years old. The Ahead of the Game intervention was featured in three studies [15,38,42].

The Help Out a Mate programme [38] was delivered by two male student volunteers with lived experience of mental illness. These volunteers were accredited in Mental Health First Aid and had a valid Working with Children Check. The volunteers were trained and were provided a manual that included a script, to help with delivery. In the study conducted by Vella et al., [15] The Help Out a Mate programme was delivered by an accredited facilitator with basic mental health training. This study also included the Your Path to Success component, which was delivered by a member of the research team, or a registered sport psychology practitioner. All the workshops were delivered at the sport club’s training grounds or on the campus of a local university.

### Intervention duration

Three of the included articles were studies relating to the Ahead of the Game intervention [15,38,42]. This intervention had 4 components, only two of which are aimed at adolescents and are of interest in this systematic review. The two parts examined included one 45-minute session entitled ‘Help Out a Mate’, another 45-minute session titled ‘Your Path to Success in Sport’, which is then supported by six online modules lasting approximately 15 minutes each. The evaluations by Liddle et al. [38] and Wynters et al. [42] focus specifically on the ‘Help Out a Mate’ session, while Vella’s review covered all components. ‘Read the Play’ is the programme which is evaluated by Patafio et al. [41]. This intervention consists of a single one-hour psychoeducational intervention. ‘Waves of Wellness’ [39] is a longer-term programme. This intervention lasted eight weeks and involved one two-hour session per week. Similar in duration is ‘Wellbeing Warriors’ [40], which is delivered over 10 weeks, with one 50/60-minute session per week.

### Intervention content

The six studies included covered four distinct interventions, including;

Wellbeing Warriors [40] is a martial arts programme that incorporates a psychoeducation component. The topics addressed includes respect, goal setting, self-concept, self-esteem, courage, resilience, bullying, peer pressure, caring for others, values, optimism, and hope, and;

Waves of Wellness [39] is a surf therapy programme for at risk adolescents. This programme involves early morning discussions on the beach which relate to physical and mental wellness, identifying healthy and unhealthy emotions, managing change, mindfulness, problem solving, asking for help, relationships, meaning making, and looking to the future. This is followed by a surf session, and;

Ahead of the Game was covered in three studies [15,38,42]. This programme is implemented in sport clubs, with modules covering what is mental health and mental illness, myths about mental illness, what is depression, what is anxiety, how to provide help, where to get reliable information, and;

Read the Play [41] is a sport-based intervention with programme content focusing on mental health, in particular mood disorders, anxiety disorders, suicide, substance use, personality disorders and accessing support if/when needed.

### Outcomes

#### Effects on mental health literacy.

The Mental Health Literacy Scale [53] was employed by Patafio et al. [41] and Liddle et al. [38], to obtain an overall mental health literacy score for each participant in the Read the Play and Ahead of the Game interventions. Patafio et al. [41] found a small effect for MHL across the intervention group (and not in the control group), for all cohorts except females. Females reported no significant changes. A statistically significant effect (p = 0.03) was found for the low scoring cohort in the intervention group from pre to post assessment. This included those who had a baseline score of 122 or below.

The results for MHL were presented by Liddle et al. [38] in two different subscales; ‘knowledge of where to seek information’ and ‘attitudes that promote recognition and reduce stigma’. The results showed no significant interaction effect for knowledge of where to seek information. However, for attitudes that promote recognition and reduce stigma, there was a significant interaction effect (p < 0.01) at all three time points and for age as a covariate.

#### Effects on help seeking behaviour.

Liddle et al. [38], Patafio et al. [41] and Vella et al. [15], used the General Help-Seeking Questionnaire [54] as a means of examining help-seeking intentions for Ahead of the Game [38,11], and Read the Play [41].

The results from Liddle et al. [38] showed no significant interaction effect in increasing help-seeking intentions from formal or informal sources. However, there was a significant increase (p < 0.05) in intentions to seek help from informal sources from all participants at follow-up, possibly due to conversations between friends or siblings.

Patafio et al. [41] found a significant effect (p < 0.03) in increasing help-seeking intentions by the low scoring participants. For this outcome, low scoring participants were those who scored 4.82 or below at the baseline measure. A significant effect was also reported across the low scoring cohort (those who scored 5.14 or below at baseline) for informal help-seeking intentions (p < 0.02), and sport-related help-seeking intentions (those who scored 4.0 or below at baseline) (p < 0.01). These results indicated a small effect on help-seeking intentions for the intervention group compared to the control group for help-seeking intentions and informal help-seeking intentions, with a medium effect for sport related help-seeking intentions.

Using the General Help-seeking Questionnaire [54], Vella et al. [15], reported a significant group-time interaction effect for help seeking from formal sources (p = 0.36), but not for informal services.

Liddle et al. [38] measured the intentions to provide help, and confidence to provide help. To understand the participants’ intentions to provide help, they were asked how likely they were to engage if they encountered someone, they thought was experiencing mental health problems. The list was comprised of six behaviours, which were drawn from the content of the programme. This measure was developed specifically for this study and therefore has not been validated. The results indicated a statistically significant interaction effect (p < 0.01), from time point one to time point two, in their intentions to provide help to others. However, this effect was not sustained one month post intervention.

To assess confidence to provide help to someone with a mental health issue, a single item was used, in which the participants answered one to five of how confident they would be (one meaning not at all confident and five meaning extremely confident) [55]. Results indicated that confidence was higher pre workshop, suggesting possible ceiling effects.

Vella et al. [15], used a single item from the Mental Health Literacy Scale [53] to assess one’s confidence to seek help. The results indicated a statistically significant group-time interaction effect in this behaviour (p = 0.03).

The Actual Help-Seeking Questionnaire (adapted from [56]) was employed by Patafio et al. [41] to measure help-seeking behaviours. The reports of help-seeking for a mental health issue did not significantly change, with the exception of the low scoring cohort (p < 0.01). The low scoring cohort included those who scored 4.82 or below at the baseline measure. The effect size was small.

#### Effects on resilience.

Three of the studies assessed resilience, all using a different scale. Vella et al. [15] utilised a 10-item version of the Connor and Davidson [57] resilience scale to measure the impact of Ahead of the Game. Results indicated a statistically significant group time interaction effect for the intervention group (p < 0.01).

McKenzie et al. [39] used the Brief Resilience Scale [58] to test the effect of Waves of Wellness of participants resilience. Mean changes on this measure indicated improvements from pre to post intervention, however these improvements were not maintained at follow-up. No statical significance was detected as a result of the small sample size (n = 9).

Moore et al. [40] assessed impact on resilience by the Wellbeing Warriors intervention, through the Child and Youth Resilience Measure [59]. The results displayed a significant difference in favour of the intervention group at post-intervention measurement compared to the baseline measure (p < 0.01), showing a moderate effect size. However, this effect was not sustained at follow up.

#### Effects on depression and anxiety literacy.

Vella et al. [15] and Liddle et al. [38] measured depression and anxiety literacy of Ahead of the Game by using 13 items from both the Depression Literacy Questionnaire [60] and the Anxiety Literacy Questionnaire [61].

The results from Vella et al. [15] showed significant effects for both depression and anxiety literacy for the intervention group, with group-time interaction effects of p < 0.01.

Similar results were reported by Liddle et al. [38], with a significant interaction effect for depression literacy from time point one to timepoint two (p < 0.01). These improvements were washed out at the follow-up measure and were not statistically significant at that time point. A statistically significant interaction also reported by Liddle et al., [38] for anxiety literacy from time point one to timepoint two (p < 0.01). These improvements were maintained at follow-up (p < 0.01).

#### Effects on stigmatizing attitudes.

An adapted version of the youth version of the Social Distance Scale [62] was used by Vella et al. [15] to assess the impact of Ahead of the Game on stigmatizing attitudes. Five items were used to assess the participants’ self-reported willingness to engage with a person with mental illness. No significant effects were found for stigmatizing attitudes (p = 0.26).

Liddle et al., [38] also assessed the impact of Ahead of the Game on stigma using the Mental Health Literacy Scale [53] which included 14 items relating to Attitudes that Promote Recognition and Reduce Stigma. Items are rated on a five-point likert scale, with some items being excluded if they were not appropriate to the demographic or content of this study. The results showed a significant effect on decreasing stigmatizing attitudes (p < 0.01) recorded for intervention versus control from baseline to immediate post, as well as baseline to follow-up.

#### Qualitative findings.

McKenzie et al. [39] conducted a qualitative analysis of Waves of Wellness through the use of semi-structured interviews. They reported changes to mental health awareness and reductions in stigma associated with mental health difficulties. Participants stated the programme helped to normalise their mental health experience and assisted in the realisation that they are not alone or different. Participants reported that the environment and enjoyment in the programme resulted in noticeable improvements in emotional wellbeing, attitudes, and confidence. Participants gained a deeper understanding of mental health, realised the need for self-care, and were able to develop coping and relaxation skills to manage negative emotions. Participants reported that the programme provided an enjoyable environment which fostered learning about mental health awareness.

Wynters et al. [42] completed a qualitative evaluation of Ahead of the Game conducting focus groups and a thematic analysis. Five themes were identified, these were: sports-based mental health promotion is engaging; an increased confidence to provide or seek help; addressing mental health stigma remains a challenge; external and internal factors shape motivation to attend workshops; workshops can be improved by being more practical and well managed. Participants improved their knowledge, attitudes and behaviours towards mental health post intervention. As a result of these changes, participants displayed an increased confidence to provide or seek help. The discussions during the focus groups suggest there was an increase in depression and anxiety literacy following the intervention. The focus groups also uncovered examples of participants using what they learned from the workshop to help support others, with one participant explaining how they called a friend at a late hour to check if they were ‘doing ok’ as they didn’t ‘seem themselves’. That participant noted that the workshop increased their confidence to talk more openly about mental health and to have the confidence to provide help to others. While positive outcomes of the programme have been highlighted, there appeared to be mixed results in relation to stigmatizing attitudes. Some adolescents experienced a change in their views surrounding mental health stigmas, reflecting that they previously did not feel comfortable talking about mental health but find it easier to do so now. However, many participants still appeared to display stigmatizing attitudes towards those with mental health symptoms, with one participant explaining they feel people say they’re depressed to get attention. Overall, the authors reported that the intervention increased MHL.

## Discussion

This systematic review was conducted in response to the limited evidence synthesis of adolescent sport-based mental health awareness interventions and provides methodological guidance and recommendations to support further research and practice. The primary focus of the review was to determine the effectiveness of programmes and in which settings and contexts, identify any effective components of the interventions, and determine whether psychological theory of behaviour change was included in any of the programme designs and their subsequent evaluations. Using an established systematic review methodology, overall findings suggest that adolescent sport-based MHL interventions can be successful in improving MHL, anxiety and depression literacy, symptom recognition, resilience, and help-seeking behaviours. Each of the six studies are summarised below, with specific recommendations outlined.

### Programme effectiveness in increasing mental health awareness.

Across the included studies, four different interventions were evaluated, including Wellbeing Warriors [40], Waves of Wellness [39], Ahead of the Game (in particular the ‘Help Out a Mate’ (HOAM) programme targeted to adolescents) [15,38,42], and Read the Play [41]. Each of the interventions had a focus on improving MHL outcomes. The results of the interventions in all six articles indicated positive effects on several indices of MHL, such as increased knowledge of mental health, depression and anxiety literacy and the recognition of disorders.

The qualitative evaluation of the Ahead of the Game intervention, conducted by Wynters et al. [42], reported increased knowledge of mental health, confidence to seek and provide help, and increased mental health first-aid intentions. The same report determined that addressing stigma remains a challenge for MHL workshops, with participants acknowledging a maintenance in stigmatizing attitudes [42].

Differing results were reported for stigmatizing attitudes in relation to the two quantitative evaluations of Ahead of the Game [15,38]. Vella et al., [15] reviewed the full Ahead of the Game programme, and found no effect on reducing stigma, however, Liddle et al., [38] focused purely on the HOAM workshop and saw a decrease in stigmatizing attitudes that was sustained over the one-month period at the follow-up evaluation. The intervention content that aimed to address stigma focused on increasing awareness of the misconceptions about mental illness. An activity was played where participants had to answer ‘true or false’ to statements relating to mental illness. Wider literature has suggested that stigma is difficult to change, with Chandra et el., [63] expressing that students’ with limited or inaccurate mental health information display more stigmatizing attitudes about individuals with mental health disorders. Based on this, perhaps promoting accurate and age-appropriate mental health information that is tailored to the participants may lead to a decrease in stigmatizing attitudes. Additionally, studies have reported that interventions which include direct contact with people with mental illness can be effective in reducing stigmatization [64]. The HOAM workshop [38] utilized facilitators with lived experience of mental illness, which may have played a role in helping bring about the change in reducing stigmatizing attitudes. While Vella et al. [15] did not report statistically significant effects for stigmatizing attitudes, it is noted that those who completed the programme per protocol reported extra benefits, such as decreases in stigmatizing attitudes. The study reported that only 30% of participants completed all adolescent components of the AOTG intervention, which may suggest why there was not a significant change for this study compared to that of Liddle et al., [38].

The quantitative evaluations of Ahead of the Game both reported findings comparable to Wynters et al., [42], as they exhibited significant benefits on depression and anxiety literacy [15,38]. The increase in anxiety literacy was sustained at the follow-up evaluation of the HOAM workshop [42]. Significant improvements in resilience, intentions to seek help from formal sources, and confidence to seek mental health information was reported for HOAM by Vella et al., [15]. Whereas Liddle et al., [38] found the HOAM workshop was not effective in increasing help-seeking intentions for formal or informal sources of support when compared to the control group, but at the follow-up both groups had an increased intention to seek help from informal services. It is likely the increase in the control group was a result of conversations between friends/siblings in the intervention group. No significant increase in intentions to seek help from informal sources was reported by Vella et al., [15]. Differences in for example, intentions to seek help, may be explained by participants who completed more of the intervention than others. Out of 283 total participants in the programme, only 91 participants completed all of the six online modules, and only 85 completed every adolescent component of the intervention [15]. While Liddle et al., [38] found HOAM significantly increased participants intentions to provide help following the completion of the programme, this effect was not sustained over the four weeks. It was also noted that participants confidence to provide help was higher prior to taking part in the HOAM workshop, suggesting possible ceiling effects [38]. A systematic review and meta-analysis of the effectiveness of interventions that promote help-seeking for mental illness [65], provided evidence that interventions can improve help-seeking for mental health. However, there was no intervention effect for the child and adolescent age group, which would be in line with some of the results reported by Vella et al., [15] and Liddle et., [38]. Overall, the Ahead of the Game programme is reported to have been effective in equipping participants with the skills to recognise mental illness symptoms, warning signs and deal with problems they may face.

Results determined by Patafio et al., [41] found that the Read the Play intervention effectively improved MHL and help-seeking intentions of those in the low scoring pre intervention cohort (those who scored 120 or below at baseline), suggesting the programme is most effective for those who are most vulnerable. It is also apparent from the data that the programme has the biggest impact, and is therefore most effective, for the younger cohort and those not previously exposed to mental health awareness. When examining cohorts separately, significant improvements are displayed in all outcomes. Significant improvements are observed for the intervention group, for all cohorts, with the exception of females and their MHL, which saw no significant change. It is difficult to compare these results to existing literature, as the two other interventions included in this review that have female participants did not measure MHL and wider literature does not separate the results by gender. This makes it difficult to determine whether it is a common theme that sport-based interventions should be tailored for females, or if the null effect is an anomaly.

The quantitative outcome evaluated by McKenzie et al., [39] in the Waves of Wellness intervention, that is of interest for this study, was related to resilience. While positive changes in resilience were reported, they were not significant [39]. While a range of inferential test options were considered and explored, due to a lack of power, they were not reported and there was no discussion of inferential results. The authors acknowledged that the study’s limited participant pool led to an inability to identify statistically significant variances in inferential analyses, thereby yielding insufficient evidential support. The qualitative data provides support for the inclusion of programmes, with participants agreeing that the programme normalised their experiences with mental health through the unique learning environment. Waves of Wellness [39] reportedly facilitated a deeper understanding of mental health and acquisitions of coping strategies. It was also noted by participants that their perceptions shifted, and they no longer felt they were suffering alone. The programme seemingly fostered personal growth and development of healthy relationships and established the importance of relaxation and self-care.

Resilience was the only measured outcome included by Moore et al., [40] that was of interest in this review, for the Wellbeing Warriors programme. The results reported significant intervention effects on participants’ total resilience, with a p value of 0.12 from baseline to post intervention [40]. The outcomes appeared stronger immediately following the intervention compared to the 12-week follow-up.

### Intervention contexts, settings, and components.

The evaluations of the included interventions provided information on which contexts and settings these programmes were most effective in. Qualitative data collect by Wynters et al., [42] confirmed the participants preferred the sport setting for mental health education rather than a school setting. Vella et al., [15] found the combination of both resilience-focused and MHL focused programming effective in the promotion of one’s mental health and wellbeing. It was also noted that the blended delivery (with the inclusion of online modules) appeared more effective than face-to-face delivery alone [15]. Components identified as effective in the qualitative assessment of Ahead of the Game [42] includes relatable sports content (including elite athlete examples), interactive content and practical tasks which increased engagement. Suggested improvements include having more practical content, using language adolescents would be more likely to use, have smaller groups for presentations and reduce distractions within the workshops. Feedback gathered by Liddle et al, [38] regarding the Ahead of the Game programme, reported that the participants enjoyed the intervention being delivered in a casual, fun and interactive way that made it easy to understand the messages. Participants stated they enjoyed learning new skills and increasing their knowledge to be able to help a friend in need [38]. In relation to intervention duration, the 45-min HOAM workshop that was delivered [38] found the magnitude of the effect of the intervention was comparable between depression and anxiety literacy and is comparable to other longer programmes [66]. This provides evidence that a brief workshop can have the ability to be as effective as longer mental health awareness interventions. However, we must recognise that the changes in depression literacy and intentions to provide help dissipated by one month post workshop. This suggests there is a need for booster sessions.

### Application of psychological theory of behaviour change.

According to the IBC [43,44] which was integrated into the design and evaluation of Ahead of the Game by Liddle et al., [38], an increase in knowledge and intentions should predict an increase in behaviour. While the results showed improvements in depression and anxiety literacy, and some improvements in intentions, these did not extend to actual helping behaviours or confidence to help someone. A factor that could have influenced the outcome is the reliance on encountering individuals with mental illness to observe acts of assistance. The reported low rate of assistance might be attributed to the participants not having had an opportunity to interact with individuals in need of help. With a significant increase in intentions to provide help reported, it is possible that the participants may be more likely to use the helping behaviours learned, to actually help someone if they do come across someone with a mental illness later in their life.

Consistent with the Socio-Ecological Approach [51], Ahead of the Game was delivered at multiple levels of influence. To the extent that Ahead of the Game was successful at increasing mental health awareness, this may have been due to the multilevel nature of the intervention, which is likely to enhance the effectiveness. While multilevel intervention is promising, it relies on reaching those on all levels and requires investment from many people. It is suggested that the differences in the perceived need to seek help may be explained that those whose parents participated in the programme may be more sensitive to the needs of their adolescent and may provide additional support. While the results do demonstrate that the program was largely successful, with positive and significant outcomes for those who participated in the programme, the intervention was only aimed at the microlevel and meso level of influence, and the individual team and club levels.

Based on the ecological dynamic’s perspective [46] Waves of Wellness [39] found that the reciprocal relationship between individual, task, and environment, was beneficial to the participants in the programme. The ocean demands holistic involvement of participants while surfing demands cognitive and emotional attention. The possibilities offered by social interactions create chances to cultivate relationships and boost self-assurance. When these opportunities are combined with the natural elements and the sport of surfing, they collectively foster personal growth by helping individuals overcome challenges and develop the essential knowledge and confidence to face future obstacles. However, the ecological dynamic’s perspective was not applied to the full programme. The intervention is lacking as the approach was only integrated to the affordances of surfing, but not applied to the mental health discussions on the beach. As a result, caution must be applied when examining the findings, as it is not clear what component of the approach is bringing about the changes and therefore the application of the ecological dynamics perspective may not be responsible for the outcomes.

Overall, there is inconsistency in how psychological behaviour change theories have been incorporated into interventions. We recommend that authors provide a clear and transparent account of how theory was employed in shaping the design and assessment of the intervention to enhance the quality and credibility of the research. This involves explicitly stating the precise theoretical concepts that were integrated, and explaining their impact on different outcomes of the study. This transparency can help both researchers and practitioners understand the theoretical underpinnings of the intervention and its potential implications. It is valuable to test the underlying theory’s assumptions and hypotheses by investigating whether the mechanisms outlined in the theory are indeed responsible for the observed impacts of the intervention.

### Limitations.

The current review included peer reviewed articles in the English language. A review which included grey literature (e.g., programmes published by government, national public health agencies, sports bodies, and mental health charitable organisations) could be considered for inclusion in a future review. The present review is limited in its capacity to determine the optimal delivery method (e.g., online, one-to-one, or group-based) and the appropriate intervention duration and frequency. It is important to acknowledge that these variables hold potential significance as screening criteria for future reviews.

## Conclusion

With a 2022 estimate that one third of young people will experience a mental health disorder in their lifetime [67], there is a need for intervention programmes designed to increase adolescent mental health awareness. The interventions evaluated in this review showed convincing results in support of sport-based mental health awareness programmes for short term effects, but perhaps not for long term effects.

While this review established positive findings and subsequent support for further development of existing sport-based mental health interventions, further studies are required with larger sample sizes and with randomisation wherein participant groups and outcome assessors are blinded. The current studies focused heavily on males. It would be suggested that there is more studies including females, to determine whether programmes of this nature are effective and suitable for females. A recent study that was conducted with males and females in Northern Ireland between the ages of 13–17 years old, found that males expressed fewer mental health challenges, displayed more favourable attitudes toward general practitioners, exhibited increased confidence in addressing mental health concerns with a doctor, and showed greater willingness to seek support from both family and medical professionals when confronted with mental health issues. [68]. In the UK higher proportions of females (compared to males) experience mental and emotional problems [68][68]. If the findings of the Breslin et al., [68] study are applied broadly, it suggests that males are more inclined to seek help for their mental health. When devising interventions to encourage adolescents to seek help, it’s essential to take into account the complexities associated with gender to ensure the interventions designed are suitable and effective for females too and not just males (Tables 3–6).

**Table 3:** Downs and Black (1998) methodological quality assessment.

Domain	Items	Study			
		Liddle et al. (2021) [38]	Moore et al. (2021) [40]	Patafio et al. (2021) [41]	Vella et al. (2021) [15]
**Reporting**	1	1	1	1	1
	2	1	1	1	1
	3	1	1	1	1
	4	1	1	1	1
	5	0	0	0	0
	6	1	1	1	1
	7	1	1	1	1
	8	0	0	0	1
	9	1	1	1	1
	10	1	1	1	1
**External Validity**	11	1	1	1	1
	12	1	1	1	1
	13	1	1	1	1
**Internal Validity- bias**	14	1	0	0	0
	15	0	0	0	0
	16	1	1	1	1
	17	1	1	0	1
	18	1	1	1	1
	19	1	0	0	1
	20	1	1	1	1
**Internal Validity- confounding**	21	1	0	0	0
	22	1	1	0	1
	23	1	1	0	0
	24	1	1	0	0
	25	0	0	0	1
	26	1	1	1	1
**Power**	27	0	1	0	1
**Total Score (out of a possible 28)**		22	20	15	21
**Quality**		Excellent	Moderate	Moderate	Excellent

**Table 4:** Critical appraisal skills programme (2018).

Study:	Wynters et al., (2021) [42]
Question	Answer
1: Was there a clear statement of the aims of the research?	Yes
2: Is a qualitative methodology appropriate?	Yes
3: Was the research design appropriate to address the aims of the research?	Yes
4: Was the recruitment strategy appropriate to the aims of the research?	Yes
5: Was the data collected in a way that addressed the research issue?	Yes
6: Has the relationship between researcher and participants been adequately considered?	Yes
7: Have ethical issues been taken into consideration?	Yes
8: Was the data analysis sufficiently rigorous?	Yes
9: Is there a clear statement of findings?	Yes
10: How valuable is the research?	Valuable

**Table 5:** Mixed methods appraisal tool (2018).

Study:	McKenzie et al., (2021) [39]
Question	Answer
5.1. Is there an adequate rationale for using a mixed methods design to address the research question?	Yes
5.2. Are the different components of the study effectively integrated to answer the research question?	Yes
5.3. Are the outputs of the integration of qualitative and quantitative components adequately interpreted?	No
5.4. Are divergences and inconsistencies between quantitative and qualitative results adequately addressed?	No
5.5. Do the different components of the study adhere to the quality criteria of each tradition of the methods involved?	Yes

**Table 6:** Outcome measures, measurement tools and main findings of the six included studies.

Authors (year of study)	Outcome measures and measurement tools	Main Findings
Liddle et al. 2021 Ahead of the Game [38]	*Intentions to Provide Help-* Unvalidated measure created with participants identifying how likely they are to engage in a list of 6 behaviours. *Depression and Anxiety Literacy-* The Depression Literacy Questionnaire [60] and the Anxiety Literacy Questionnaire [61]. 13 of the 22 items for each measure were used as remaining items capture knowledge of effective treatments that were not included in the HOAM content. *Intentions to Seek Help-* General Help Seeking Questionnaire [54] *Confidence to Provide Help-* Single item question asking how confident they are to help someone [55] *Mental Health Literacy-* Two subscales of the Mental Health Literacy Scale [53]	Statistically significant interaction effect (p < 0.01) in intentions to provide help post attendance at the workshop, however this was not sustained 4 weeks post workshop. Significant interaction effect for depression and anxiety literacy for the intervention group vs. control group, time point one verse time point two (p < 0.01). Effects for depression literacy were washed out at follow-up, but were sustained for anxiety literacy (p < 0.01). Increased intentions to seek help from informal services at follow-up by both groups (this may be because of conversations between friends or siblings). Confidence to provide help was higher pre workshop, suggestions possible ceiling effects. Significant interaction effect on decreasing stigmatizing attitudes at all three time points and against age as a covariate (p > 0.01).
McKenzie et al., 2021 Waves of Wellness [39]	*Brief resiliency scale-* Participants reported how quickly they would bounce back or recover from stress on six items using a one (strongly disagree) to five (strongly agree) scale. [58] *Semi structured interviews*	Improvements in resilience post intervention, however, not maintained at follow up. Key overarching theme that came out of the interviews was noted as ‘a shared experience’. This is split into two lower order themes; a unique learning environment and personal growth.
Moore et al.2021 Wellbeing Warriors [40]	*Child and youth resilience measure -* 28 items to test total resilience which was the primary outcome [59]	Statistically significant effects in favour of the intervention group vs control from baseline to post- intervention (p < 0.01).
Patafio et al. 2021 Read the Play [41]	*Mental health literacy*- Mental Health Literacy Scale [53] with modifications *Help-seeking intentions*- General Help-Seeking Questionnaire [54]. 11 help sources included plus space to specify help source not listed in options provided. *Help-seeking behaviours*- Actual Help-Seeking Questionnaire (adapted from [56])	Significant intervention effects mental health literacy, for the low-scoring youth cohort (p = 0.03). Statistically significant intervention effects recorded for help-seeking intentions (p < 0.03), and specifically for informal (p < 0.02) and sport-related help-seeking intentions (p < 0.01), all by the low scoring cohort. Reports of actual help-seeking did not significantly change, with the exception of the low scoring cohort (p = 0.01). Qualitative assessment found there was reduced stigma associated with mental health difficulties. Normalised mental health experiences. Facilitated deeper understandings of mental health and acquisition of coping strategies.
Vella et al. 2021 Ahead of the Game [15]	*Depression and anxiety literacy*- 13 items of the Depression Literacy Questionnaire [60] and 13 items of the Anxiety Literacy Questionnaire [61] *Resilience*- 10 item version of the Connor-Davidson Resilience Scale [57] *Confidence to seek-help*- Single item from the Mental Health Literacy Scale [53] *Help-seeking intentions*- The General Help Seeking Questionnaire [54] *Stigmatizing attitudes*- The youth version of the Social Distance Scale (adapted) [62]	Significant improvements in depression and anxiety literacy for the intervention group, with group-time interaction effect of p < 0.01. Significant improvements in resilience with an interaction effect of p < 0.01. Significant interaction effect for confidence to seek help (p=<0.03). Significant group-time interaction effect in intentions to seek help from formal sources (p < 0.01). No significant effects for stigmatizing attitudes.
Wynters et al., 2021 Ahead of the Game [42]	No outcome measures quantitatively assessed	Key themes that came up in conversation were: Increased confidence to provide or seek help. Addressing mental health stigma remains a challenge.

Not all the studies including a comparison/control group acted as a limitation when analysing data, making it hard to make conclusions on the best practice for interventions of this nature. Additional considerations that would improve the quality of support in the research in this area would include having age matched controls, longer periods for follow-up to test long term effectiveness, and the inclusion of booster sessions. Future research should have a focus on the systematic application of theoretical frameworks in the design, implementation and evaluation of interventions targeted at sports-based promotion of adolescent mental health awareness. It would be suggested that mental health programme designers and evaluators consider the limitations and recommendations raised in this review.

## Supporting information

S1 File PRISMA Checklist. (DOCX)

S2 File Data Extraction Table. (DOCX)

S3 File Title and Abstract Screening Reviewer Breakdown. (DOCX)

S4 File Sample of Excluded Titles. (DOCX)

S5 File Data Extracted from Included Studies. (DOCX)

S6 File Methodological Quality Assessments. (DOCX)
