# Supplementary material for: A systematic review of sport-based adolescent mental health awareness programmes
Source: PLoS One. 2025 Mar 27;20(3):e0315315. doi: 10.1371/journal.pone.0315315 (PMC11949344; doi:10.1371/journal.pone.0315315)
Supplement: S3 File — (DOCX) [file pone.0315315.s003.docx]

S3: Title and Abstract Screening Reviewer Breakdown

| **Reviewer** | **Contribution** |
| --- | --- |
| Nora Sullivan | 10,408 |
| Marian McLaughlin | 614 |
| Gavin Breslin | 612 |
| Stephen Shannon | 527 |
