# Supplementary material for: A systematic review of sport-based adolescent mental health awareness programmes
Source: PLoS One. 2025 Mar 27;20(3):e0315315. doi: 10.1371/journal.pone.0315315 (PMC11949344; doi:10.1371/journal.pone.0315315)
Supplement: S4 File — (DOCX) [file pone.0315315.s004.docx]

S4: Sample of Excluded Titles

*All studies in the table were excluded for one of the following reasons: irrelevant; wrong outcomes; wrong population; no intervention; wrong intervention delivery (e.g. no sport component)

| **Title** | **Authors** | **Published Year** | **Journal** | **DOI** |
| --- | --- | --- | --- | --- |
| Fitness training for cardiorespiratory conditioning after traumatic brain injury | Hassett, L; Moseley, AM; Harmer, AR | 2017 | Cochrane Database of Systematic Reviews | 10.1002/14651858.CD006123.pub3 |
| Physical exercise training interventions for children and young adults during and after treatment for childhood cancer | Braam, KI; van der Torre, P; Takken, T; Veening, MA; van Dulmen‐den Broeder, E; Kaspers, GJL | 2016 | Cochrane Database of Systematic Reviews | 10.1002/14651858.CD008796.pub3 |
| Video games for people with schizophrenia | Roberts, MT; Lloyd, J; Välimäki, M; Ho, GWK; Freemantle, M; Békefi, AZ | 2021 | Cochrane Database of Systematic Reviews | 10.1002/14651858.CD012844.pub2 |
| Later school start times for supporting the education, health, and well‐being of high school students | Marx, R; Tanner‐Smith, EE; Davison, CM; Ufholz, LA; Freeman, J; Shankar, R; Newton, L; Brown, RS; Parpia, AS; Cozma, I; et al. | 2017 | Cochrane Database of Systematic Reviews | 10.1002/14651858.CD009467.pub2 |
| Exercise for pregnant women with pre‐existing diabetes for improving maternal and fetal outcomes | Brown, J; Ceysens, G; Boulvain, M | 2017 | Cochrane Database of Systematic Reviews | 10.1002/14651858.CD012696.pub2 |
| Exercise for pregnant women with gestational diabetes for improving maternal and fetal outcomes | Brown, J; Ceysens, G; Boulvain, M | 2017 | Cochrane Database of Systematic Reviews | 10.1002/14651858.CD012202.pub2 |
| Psychological treatments for people with epilepsy | Michaelis, R; Tang, V; Nevitt, SJ; Wagner, JL; Modi, AC; LaFrance Jr, WCurt; Goldstein, LH; Gandy, M; Bresnahan, R; Valente, K; et al. | 2020 | Cochrane Database of Systematic Reviews | 10.1002/14651858.CD012081.pub3 |
| Life skills programmes for chronic mental illnesses | Tungpunkom, P; Maayan, N; Soares‐Weiser, K | 2012 | Cochrane Database of Systematic Reviews | 10.1002/14651858.CD000381.pub3 |
| E‐Health interventions for anxiety and depression in children and adolescents with long‐term physical conditions | Thabrew, H; Stasiak, K; Hetrick, SE; Wong, S; Huss, JH; Merry, SN | 2018 | Cochrane Database of Systematic Reviews | 10.1002/14651858.CD012489.pub2 |
| Psychological therapies (remotely delivered) for the management of chronic and recurrent pain in children and adolescents | Fisher, E; Law, E; Dudeney, J; Eccleston, C; Palermo, TM | 2019 | Cochrane Database of Systematic Reviews | 10.1002/14651858.CD011118.pub3 |
| Psychological interventions for people with hemophilia | Palareti, L; Melotti, G; Cassis, F; Nevitt, SJ; Iorio, A | 2020 | Cochrane Database of Systematic Reviews | 10.1002/14651858.CD010215.pub2 |
| Psychological therapies for the management of chronic and recurrent pain in children and adolescents | Fisher, E; Law, E; Dudeney, J; Palermo, TM; Stewart, G; Eccleston, C | 2018 | Cochrane Database of Systematic Reviews | 10.1002/14651858.CD003968.pub5 |
| Psychosocial interventions for conversion and dissociative disorders in adults | Ganslev, CA; Storebø, OJ; Callesen, HE; Ruddy, R; Søgaard, U | 2020 | Cochrane Database of Systematic Reviews | 10.1002/14651858.CD005331.pub3 |
| A realist review of which advocacy interventions work for which abused women under what circumstances | Rivas, C; Vigurs, C; Cameron, J; Yeo, L | 2019 | Cochrane Database of Systematic Reviews | 10.1002/14651858.CD013135.pub2 |
| Transcutaneous electrical nerve stimulation (TENS) for fibromyalgia in adults | Johnson, MI; Claydon, LS; Herbison, GP; Jones, G; Paley, CA | 2017 | Cochrane Database of Systematic Reviews | 10.1002/14651858.CD012172.pub2 |
| Client feedback in psychological therapy for children and adolescents with mental health problems | Bergman, H; Kornør, H; Nikolakopoulou, A; Hanssen‐Bauer, K; Soares‐Weiser, K; Tollefsen, TK; Bjørndal, A | 2018 | Cochrane Database of Systematic Reviews | 10.1002/14651858.CD011729.pub2 |
| Dance movement therapy for depression | Meekums, B; Karkou, V; Nelson, EA | 2015 | Cochrane Database of Systematic Reviews | 10.1002/14651858.CD009895.pub2 |
| Interventions for sexual dysfunction following stroke | Stratton, H; Sansom, J; Brown-Major, A; Anderson, P; Ng, L | 2020 | Cochrane Database of Systematic Reviews | 10.1002/14651858.CD011189.pub2 |
| Oral anti‐diabetic pharmacological therapies for the treatment of women with gestational diabetes | Brown, J; Martis, R; Hughes, B; Rowan, J; Crowther, CA | 2017 | Cochrane Database of Systematic Reviews | 10.1002/14651858.CD011967.pub2 |
| Lithium for acute mania | McKnight, RF; de La Motte de Broöns de Vauvert, SJ; Chesney, E; Amit, BH; Geddes, J; Cipriani, A | 2019 | Cochrane Database of Systematic Reviews | 10.1002/14651858.CD004048.pub4 |
| Taxation of the fat content of foods for reducing their consumption and preventing obesity or other adverse health outcomes | Lhachimi, SK; Pega, F; Heise, TL; Fenton, C; Gartlehner, G; Griebler, U; Sommer, I; Bombana, M; Katikireddi, SVittal | 2020 | Cochrane Database of Systematic Reviews | 10.1002/14651858.CD012415.pub2 |
| Psychosocial interventions for recurrent abdominal pain in childhood | Abbott, RA; Martin, AE; Newlove‐Delgado, TV; Bethel, A; Thompson‐Coon, J; Whear, R; Logan, S | 2017 | Cochrane Database of Systematic Reviews | 10.1002/14651858.CD010971.pub2 |
| Non‐clinical interventions for reducing unnecessary caesarean section | Chen, I; Opiyo, N; Tavender, E; Mortazhejri, S; Rader, T; Petkovic, J; Yogasingam, S; Taljaard, M; Agarwal, S; Laopaiboon, M; et al. | 2018 | Cochrane Database of Systematic Reviews | 10.1002/14651858.CD005528.pub3 |
| Interventionist versus expectant care for severe pre‐eclampsia between 24 and 34 weeks' gestation | Churchill, D; Duley, L; Thornton, JG; Moussa, M; Ali, HSM; Walker, KF | 2018 | Cochrane Database of Systematic Reviews | 10.1002/14651858.CD003106.pub3 |
| Conservative interventions for treating functional daytime urinary incontinence in children | Buckley, BS; Sanders, CD; Spineli, L; Deng, Q; Kwong, JSW | 2019 | Cochrane Database of Systematic Reviews | 10.1002/14651858.CD012367.pub2 |
| Drug management for acute tonic‐clonic convulsions including convulsive status epilepticus in children | McTague, A; Martland, T; Appleton, R | 2018 | Cochrane Database of Systematic Reviews | 10.1002/14651858.CD001905.pub3 |
| Virtual reality distraction for acute pain in children | Lambert, V; Boylan, P; Boran, L; Hicks, P; Kirubakaran, R; Devane, D; Matthews, A | 2020 | Cochrane Database of Systematic Reviews | 10.1002/14651858.CD010686.pub2 |
| Automated telephone communication systems for preventive healthcare and management of long‐term conditions | Posadzki, P; Mastellos, N; Ryan, R; Gunn, LH; Felix, LM; Pappas, Y; Gagnon, MP; Julious, SA; Xiang, L; Oldenburg, B; et al. | 2016 | Cochrane Database of Systematic Reviews | 10.1002/14651858.CD009921.pub2 |
| Relaxation therapy for preventing and treating preterm labour | Khianman, B; Pattanittum, P; Thinkhamrop, J; Lumbiganon, P | 2012 | Cochrane Database of Systematic Reviews | 10.1002/14651858.CD007426.pub2 |
| Interventions for improving sleep quality in people with chronic kidney disease | Natale, P; Ruospo, M; Saglimbene, VM; Palmer, SC; Strippoli, GFM | 2019 | Cochrane Database of Systematic Reviews | 10.1002/14651858.CD012625.pub2 |
| Neuraminidase inhibitors for preventing and treating influenza in adults and children | Jefferson, T; Jones, MA; Doshi, P; Del Mar, CB; Hama, R; Thompson, MJ; Spencer, EA; Onakpoya, IJ; Mahtani, KR; Nunan, D; et al. | 2014 | Cochrane Database of Systematic Reviews | 10.1002/14651858.CD008965.pub4 |
| Management of gag reflex for patients undergoing dental treatment | Eachempati, P; Kumbargere Nagraj, S; Kiran Kumar Krishanappa, S; George, RP; Soe, HH; Karanth, L | 2019 | Cochrane Database of Systematic Reviews | 10.1002/14651858.CD011116.pub3 |
| Fetal biometry for guiding the medical management of women with gestational diabetes mellitus for improving maternal and perinatal health | Rao, U; de Vries, B; Ross, GP; Gordon, A | 2019 | Cochrane Database of Systematic Reviews | 10.1002/14651858.CD012544.pub2 |
| Treatments for women with gestational diabetes mellitus: an overview of Cochrane systematic reviews | Martis, R; Crowther, CA; Shepherd, E; Alsweiler, J; Downie, MR; Brown, J | 2018 | Cochrane Database of Systematic Reviews | 10.1002/14651858.CD012327.pub2 |
| Non‐pharmacological interventions for depression in adults and children with traumatic brain injury | Gertler, P; Tate, RL; Cameron, ID | 2015 | Cochrane Database of Systematic Reviews | 10.1002/14651858.CD009871.pub2 |
| Interventions for women in subsequent pregnancies following obstetric anal sphincter injury to reduce the risk of recurrent injury and associated harms | Farrar, D; Tuffnell, DJ; Ramage, C | 2014 | Cochrane Database of Systematic Reviews | 10.1002/14651858.CD010374.pub2 |
| Lifestyle interventions for the treatment of women with gestational diabetes | Brown, J; Alwan, NA; West, J; Brown, S; McKinlay, CJD; Farrar, D; Crowther, CA | 2017 | Cochrane Database of Systematic Reviews | 10.1002/14651858.CD011970.pub2 |
| Combined diet and exercise interventions for preventing gestational diabetes mellitus | Shepherd, E; Gomersall, JC; Tieu, J; Han, S; Crowther, CA; Middleton, P | 2017 | Cochrane Database of Systematic Reviews | 10.1002/14651858.CD010443.pub3 |
| Insulin for the treatment of women with gestational diabetes | Brown, J; Grzeskowiak, L; Williamson, K; Downie, MR; Crowther, CA | 2017 | Cochrane Database of Systematic Reviews | 10.1002/14651858.CD012037.pub2 |
| Self‐management for bronchiectasis | Kelly, C; Grundy, S; Lynes, D; Evans, DJW; Gudur, S; Milan, SJ; Spencer, S | 2018 | Cochrane Database of Systematic Reviews | 10.1002/14651858.CD012528.pub2 |
| (WHI03) Youth with Multiple Sclerosis Have Low Fitness Levels...2020 Virtual Annual Meeting of the Consortium of Multiple Sclerosis Centers, May 26-29, 2020. | Stephens, Samantha; Schneiderman, Jane E.; Berenbaum, Tara; Finlayson, Marcia; Motl, Robert W.; Yeh, E. Ann | 2020 | International Journal of MS Care |  |
| “In the Sport I Am Here”: Therapeutic Processes and Health Effects of Sport and Exercise on PTSD. | Ley, Clemens; Rato Barrio, María; Koch, Andreas | 2018 | Qualitative Health Research | 10.1177/1049732317744533 |
| A 12 Week Remote Based Physical Activity Intervention In Young Adults With Autism Spectrum Disorder: 1223...American College of Sports Medicine, Annual Meeting and World Congresses, May 31-June 4, 2022, San Diego, California. | Leahy, Nicholas; Garcia, Jeanette | 2022 | Medicine & Science in Sports & Exercise | 10.1249/01.mss.0000878784.14054.b5 |
| A case of life style disorder and homoeopathy. | Rajan, S. Sabari; Rani, C. T. Nisha | 2020 | Homoeopathic Heritage |  |
| A Combined Sleep Hygiene and Mindfulness Intervention to Improve Sleep and Well-Being During High-Performance Youth Tennis Tournaments. | Lever, Jonathon R.; Murphy, Alistair P.; Duffield, Rob; Fullagar, Hugh H.K. | 2021 | International Journal of Sports Physiology & Performance | 10.1123/ijspp.2019-1008 |
| A core outcome set for school-based physical activity interventions: an international consensus...14th European Public Health Conference (Virtual), Public health futures in a changing world, November 10-12, 2021. | Ram, B; Foley, K; Sluijs, E van; Hargreaves, D; Viner, R; Saxena, S | 2021 | European Journal of Public Health |  |
| A Mindfulness-Based Program among Adolescent Boys with Behavior Disorders: A Quasi-Experimental Study. | Roux, Benjamin; Philippot, Pierre | 2020 | Journal of Child & Family Studies | 10.1007/s10826-020-01751-z |
| A nationwide web-based survey of factors associated with depressive symptoms among Japanese workers. | Saito, Shota; Qi, Ruan; Tran, Huyen Thi Thanh; Suzuki, Kenji; Takiguchi, Toru; Noto, Shinichi; Ohde, Sachiko; Takahashi, Osamu | 2022 | International Journal of Social Psychiatry | 10.1177/00207640211017586 |
| A Phase I Study Examining the Feasibility and Safety of an Aerobic Exercise Intervention in Patients With Rectal Cancer During and After Neoadjuvant Chemoradiotherapy. | Morielli, Andria R.; Usmani, Nawaid; Boulé, Normand G.; Tankel, Keith; Severin, Diane; Nijjar, Tirath; Joseph, Kurian; Courneya, Kerry S. | 2016 | Oncology Nursing Forum | 10.1188/16.ONF.352-362 |
| A prospective longitudinal investigation of the (dis)continuity of mental health difficulties between mid- to late-childhood and the predictive role of familial factors. | O’Connor, Cliodhna; Reulbach, Udo; Gavin, Blanaid; McNicholas, Fiona | 2018 | European Child & Adolescent Psychiatry | 10.1007/s00787-017-1044-5 |
| A self-efficacy-enhancing intervention for Chinese patients after total hip arthroplasty: study protocol for a randomized controlled trial with 6-month follow-up. | Deng, Bo; Chen, Yumei; Meng, Ya; Zhang, Yiheng; Tan, Xingxian; Zhou, Xiaohong; Zhang, Meifen | 2022 | Journal of Orthopaedic Surgery & Research | 10.1186/s13018-021-02689-8 |
| A Virtual Resiliency Intervention Promoting Resiliency for Parents of Children with Learning and Attentional Disabilities: A Randomized Pilot Trial. | Park, Elyse R.; Perez, Giselle K.; Millstein, Rachel A.; Luberto, Christina M.; Traeger, Lara; Proszynski, Jacqueline; Chad-Friedman, Emma; Kuhlthau, Karen A. | 2020 | Maternal & Child Health Journal | 10.1007/s10995-019-02815-3 |
| A web-based intervention to promote physical activity in adolescents and young adults with cystic fibrosis: protocol for a randomized controlled trial. | Cox, Narelle S.; Eldridge, Beverley; Rawlings, Sarah; Dreger, Julianna; Corda, Jennifer; Hauser, Jennifer; Button, Brenda M.; Bishop, Jennifer; Nichols, Amanda; Middleton, Anna; Ward, Nathan; Dwyer, Tiffany; Tomlinson, Owen W.; Denford, Sarah; Barker, Alan R.; Williams, Craig A.; Kingsley, Michael; O'Halloran, Paul; Holland, Anne E. | 2019 | BMC Pulmonary Medicine | 10.1186/s12890-019-0942-3 |
| Active Commuting to School, Physical Activity, and Behavior Problems Among Third‐Grade Children. | Mann, Mana; Silver, Ellen J.; Stein, Ruth E. K. | 2018 | Journal of School Health | 10.1111/josh.12677 |
| Adolescent Concussion and Mental Health Outcomes: A Population-based Study. | Yang, Max N.; Clements-Nolle, Kristen; Parrish, Brian; Wei Yang | 2019 | American Journal of Health Behavior | 10.5993/AJHB.43.2.3 |
| Adolescent Sport Participation and Symptoms of Anxiety and Depression: A Systematic Review and Meta-Analysis. | Panza, Michael J.; Graupensperger, Scott; Agans, Jennifer P.; Doré, Isabelle; Vella, Stewart A.; Evans, Michael Blair | 2020 | Journal of Sport & Exercise Psychology | 10.1123/jsep.2019-0235 |
| Adolescents and young adults with asthma and allergies: Physical activity, self-efficacy, social support, and subsequent psychosocial outcomes. | Suorsa, Kristina I.; Cushing, Christopher C.; Mullins, Alexandria J.; Meier, Ellen; Tackett, Alayna P.; Junghans, Ashley; Chaney, John M.; Mullins, Larry L. | 2016 | Children's Health Care | 10.1080/02739615.2015.1065741 |
| ADULTS WITH LOEYS-DIETZ SYNDROME AND VASCULAR EHLERS-DANLOS SYNDROME: A CROSS-SECTIONAL STUDY OF LIFE SATISFACTION. | JOHANSEN, Heidi; VELVIN, Gry; FUGL-MEYER, Kerstin; LIDAL, Ingeborg Beate | 2021 | Journal of Rehabilitation Medicine (Stiftelsen Rehabiliteringsinformation) | 10.2340/jrm.v53.572 |
| Adverse Childhood Experiences and the Risk of Coronary Heart Disease in Adulthood: Examining Potential Psychological, Biological, and Behavioral Mediators in the Whitehall II Cohort Study. | Deschênes, Sonya S.; Kivimaki, Mika; Schmitz, Norbert | 2021 | Journal of the American Heart Association | 10.1161/JAHA.120.019013 |
| Age Differences in Death and Suicidal Ideation in Anxious Primary Care Patients. | Petkus, Andrew J.; Wetherell, Julie Loebach; Stein, Murray B.; Chavira, Denise A.; Craske, Michelle G.; Sherbourne, Cathy; Sullivan, Greer; Bystritsky, Alexander; Roy-Byrne, Peter | 2018 | Clinical Gerontologist | 10.1080/07317115.2017.1356893 |
| Allergic Disease, Short Sleep Duration, and Suicidal Ideation and Plans Among Korean Adolescents. | Kim, Ji-Su; Seo, Yeji | 2022 | Journal of School Nursing | 10.1177/1059840520921920 |
| An At-home Positive Psychology Intervention for Individuals with Multiple Sclerosis: A Phase 1 Randomized Controlled Trial. | Freedman, Melanie E.; Healy, Brian C.; Huffman, Jeff C.; Chitnis, Tanuja; Weiner, Howard L.; Glanz, Bonnie I. | 2021 | International Journal of MS Care | 10.7224/1537-2073.2020-020 |
| An integrative review of fatigue in adults with type 2 diabetes mellitus: Implications for self‐management and quality of life. | Kuo, Hsuan‐Ju; Huang, Ya‐Ching; García, Alexandra A. | 2022 | Journal of Clinical Nursing (John Wiley & Sons, Inc.) | 10.1111/jocn.16058 |
| Analysis of Current Situation and Influencing Factors of Psychological Distress in Patients with Lung Cancer during Perioperative Period. | He, Xin; Zhang, Na; Liu, Lu; Liu, Yan | 2022 | Evidence-based Complementary & Alternative Medicine (eCAM) | 10.1155/2022/1925668 |
| Anxiety and depression in patients with pulmonary hypertension: impact and management challenges. | Bussotti, Maurizio; Sommaruga, Marinella | 2018 | Vascular Health & Risk Management | 10.2147/VHRM.S147173 |
| ANXIETY, FEAR, AND MOVEMENT FOLLOWING SPORTS‐RELATED CONCUSSION: HOW DOES KINESIOPHOBIA CORRELATE TO SYMPTOMS AND REACTION TIME?...Pediatric Research in Sports Medicine (PRiSM), 8th Annual Meeting, 28-30 January, 2021 | Reinking, Sarah; Seehusen, Corrine N.; Walker, Gregory A.; Wilson, Julie C.; Howell, David R. | 2021 | Orthopaedic Journal of Sports Medicine | 10.1177/2325967121S00057 |
| APOLO-Teens, a web-based intervention for treatment-seeking adolescents with overweight or obesity: study protocol and baseline characterization of a Portuguese sample. | Ramalho, Sofia; Saint-Maurice, Pedro F.; Silva, Diana; Mansilha, Helena Ferreira; Silva, Cátia; Gonçalves, Sónia; Machado, Paulo; Conceição, Eva | 2020 | Eating & Weight Disorders | 10.1007/s40519-018-0623-x |
| Are pre- and early pregnancy lifestyle factors associated with the risk of preterm birth? A secondary cohort analysis of the cluster-randomised GeliS trial. | Raab, Roxana; Hoffmann, Julia; Spies, Monika; Geyer, Kristina; Meyer, Dorothy; Günther, Julia; Hauner, Hans | 2022 | BMC Pregnancy & Childbirth | 10.1186/s12884-022-04513-5 |
| Aspects of Depression Among Socioeconomically Disadvantaged African American Young Adults. | Kosma, Maria; Buchanan, David R. | 2019 | International Quarterly of Community Health Education | 10.1177/0272684X19829612 |
| Assessment of Turkish Patients' Knowledge about Management of Cardiac Risk Factors. | Akten, Ilknur Metin; Akin, Semiha; Kurbun, Hava; Erbatu, Betul | 2021 | International Journal of Caring Sciences |  |
| Association between 90o push-up and cardiorespiratory fitness: cross-sectional evidence of push-up as a tractable tool for physical fitness surveillance in youth. | Ajisafe, Toyin | 2019 | BMC Pediatrics | 10.1186/s12887-019-1840-9 |
| Association Between Adherence to Healthy Lifestyles and Depressive Symptoms Among Japanese Hospital Workers During the COVID-19 Pandemic. | Fukunaga, Ami; Inoue, Yosuke; Yamamoto, Shohei; Miki, Takako; Nanri, Akiko; Ishiwari, Hironori; Ishii, Masamichi; Miyo, Kengo; Konishi, Maki; Ohmagari, Norio; Mizoue, Tetsuya | 2021 | Asia-Pacific Journal of Public Health | 10.1177/10105395211007604 |
| Association between cardiorespiratory fitness and depressive symptoms in children and adolescents: A systematic review and meta-analysis. | Alves Donato, Arthur Ney; Waclawovsky, Aline Josiane; Tonello, Laís; Firth, Joseph; Smith, Lee; Stubbs, Brendon; Schuch, Felipe Barreto; Boullosa, Daniel | 2021 | Journal of Affective Disorders | 10.1016/j.jad.2021.01.032 |
| Association between Physical Activity, Food Consumption and Depressive Symptoms Among Young Adults in Spain: Findings of a National Survey. | Debbia, Fabio; Rodríguez-Muñoz, Pedro Manuel; Carmona-Torres, Juan Manuel; Hidalgo-Lopezosa, Pedro; Cobo-Cuenca, Ana Isabel; López-Soto, Pablo Jesús; Rodríguez-Borrego, Maria Aurora | 2020 | Issues in Mental Health Nursing | 10.1080/01612840.2019.1672223 |
| ASSOCIATION BETWEEN SADNESS AND SYMPTOM SEVERITY IN PEDIATRIC AND ADOLESCENT PATIENTS WITH HIP PAIN...Pediatric Research in Sports Medicine Society (PRiSM) 7th Annual Meeting, January 23-25, 2020, Glendale, Arizona. | Wagner, K. John; Tigabu, Kirubel; Sabatino, Meagan J.; Richard, Heather M.; Podeszwa, David A.; Sucato, Daniel J.; Ellis Jr., Henry B. | 2020 | Orthopaedic Journal of Sports Medicine | 10.1177/2325967120S00259 |
| Association between Sleep Duration, Physical Activity, and Mental Health Disorders: A Secondary Analysis of the National Survey of Children's Health 2017-2018. | Xiang, Shiting; Dong, Jie; Li, Xun; Li, Liping | 2021 | BioMed Research International | 10.1155/2021/5585678 |
| Association of Anxiety-Related Polymorphisms with Sports Performance in Chilean Long Distance Triathletes: A Pilot Study. | Sanhueza, Jorge A.; Zambrano, Tomás; Bahamondes-Avila, Carlos; Salazar, Luis A. | 2016 | Journal of Sports Science & Medicine |  |
| Association of Team Sports Participation With Long-term Mental Health Outcomes Among Individuals Exposed to Adverse Childhood Experiences. | Easterlin, Molly C.; Chung, Paul J.; Leng, Mei; Dudovitz, Rebecca | 2019 | JAMA Pediatrics | 10.1001/jamapediatrics.2019.1212 |
| Associations between backache and stress among undergraduate students. | Ben-Ami, Noa; Korn, Liat | 2020 | Journal of American College Health | 10.1080/07448481.2018.1515753 |
| Asthma-specific cognitions, self-focused attention, and fear of negative evaluation in adolescents and young adults diagnosed with childhood-onset asthma. | Junghans-Rutelonis, Ashley N.; Tackett, Alayna P.; Suorsa, Kristina I.; Chaney, John M.; Mullins, Larry L. | 2018 | Psychology, Health & Medicine | 10.1080/13548506.2017.1325507 |
| ATHLETE IDENTITY AND COMMON BENEFITS AND BARRIERS TO SPORT PARTICIPATION AMONGST ADOLESCENT SLED HOCKEY PLAYERS...Pediatric Research in Sports Medicine (PRiSM), 8th Annual Meeting, 28-30 January, 2021. | Napolitano, Jonathan; Iliescu, Daniela; Hoehn, Jessica; Berner, Theresa; Meyer, Carlie; Jackson, Kristen; Kemp, Erika | 2021 | Orthopaedic Journal of Sports Medicine | 10.1177/2325967121S00078 |
| ATHLETIC IDENTITY IN YOUTH ATHLETES: A SYSTEMATIC REVIEW...Pediatric Research in Sports Medicine (PRiSM), 8th Annual Meeting, 28-30 January, 2021. | Edison, Bianca; Rizzone, Katherine; Christino, Melissa | 2021 | Orthopaedic Journal of Sports Medicine | 10.1177/2325967121S00102 |
| Barriers to Education of Syrian Refugee Girls in Jordan: Gender‐Based Threats and Challenges. | Hattar‐Pollara, Marianne | 2019 | Journal of Nursing Scholarship | 10.1111/jnu.12480 |
| Basketball as a Complementary Treatment for Physical and Psychosocial Rehabilitation. | Doari, Yedidiya; Mittleman, Jerry | 2021 | Palaestra |  |
| Being a teen and learning how to surf anxiety: Integrating narrative methods with cognitive–behavioral therapy. | Vale Lucas, Carla; Soares, Luísa | 2014 | Journal of Poetry Therapy | 10.1080/08893675.2014.895489 |
| Beyond Pain: Juvenile Fibromyalgia Management for the Pediatric Nurse. | Ma, Elizabeth Hannah; McCauley, Sabrina Opiola | 2019 | Pediatric Nursing |  |
| Bingeing, Purging, and Suicidal Ideation in Clinical and Non-Clinical Samples of Youth. | Obeid, Nicole; Norris, Mark L.; Valois, Darcie D.; Buchholz, Annick; Goldfield, Gary S.; Hadjiyannakis, Stasia; Henderson, Katherine A.; Flament, Martine; Hammond, Nicole G.; Dunn, Jessica; Spettigue, Wendy | 2020 | Eating Disorders | 10.1080/10640266.2019.1642033 |
| Building Emotional Intelligence for Student Success: A Pilot Study. | Shah, Amee P.; Galantino, Mary Lou | 2019 | Perspectives of the ASHA Special Interest Groups | 10.1044/2019_PERSP-19-00101 |
| Bullying Environment Moderates the Relationship Between Exercise and Mental Health in Bullied US Children. | Sibold, Jeremy; Edwards, Erika M.; O'Neil, Linnae; Murray‐Close, Dianna; Hudziak, James J. | 2020 | Journal of School Health | 10.1111/josh.12864 |
| Bullying victimization and obesogenic behaviour among adolescents aged 12 to 15 years from 54 low‐ and middle‐income countries. | Smith, Lee; Jacob, Louis; Shin, Jae Il; Tully, Mark A; Pizzol, Damiano; López‐Sánchez, Guillermo F.; Gorely, Trish; Yang, Lin; Grabovac, Igor; Koyanagi, Ai | 2021 | Pediatric Obesity | 10.1111/ijpo.12700 |
| Cardiac Concerns in the Pediatric Athlete. | Colombo, Jamie N.; Sawda, Christine N.; White, Shelby C. | 2022 | Clinics in Sports Medicine | 10.1016/j.csm.2022.02.010 |
| CEC QUIZ. |  | 2021 | IDEA Fitness Journal |  |
| Central or overall obesity: which one is a better predictor of depressive symptoms in children, adolescents, and youths? | Esmaeilzadeh, Samad; Farzizadeh, Reza; Kalantari, Hassan-Ali; Mahmoudi, Asghar; Bilehsavar, Omid Yousefi; Mehranpour, Ali | 2018 | Eating & Weight Disorders | 10.1007/s40519-016-0320-6 |
| Characteristics of adolescent athletes seeking early versus late care for sport-related concussion. | Germann, Darrin; Cancelliere, Carol; Kazemi, Mohsen | 2021 | Journal of the Canadian Chiropractic Association |  |
| CHILD & ADOLESCENT PSYCHIATRY. Bullying and Depression in Youths. | Dineen Wagner, Karen | 2016 | Psychiatric Times |  |
| Child- compared with parent-report ratings on psychosocial measures following a mild traumatic brain injury among youth with persistent post-concussion symptoms. | Johnson, Ashleigh M; McCarty, Carolyn A; Marcynyszyn, Lyscha A; Zatzick, Douglas F; Chrisman, Sara PD; Rivara, Frederick P | 2021 | Brain Injury | 10.1080/02699052.2021.1889663 |
| CLINICAL FEATURES, TREATMENT AND REHABILITATION OF NEW CORONAVIRUS INFECTION IN PATIENTS WITH METABOLIC SYNDROME. | Ivanov, Dmitry O.; Uspenskiy, Yury P.; Sarana, Andrey M.; Fominykh, Yulia A.; Sousova, Iana V.; Zakharov, Dmitry V. | 2021 | Pediatrician (2079-7850) | 10.17816/PED1255-25 |
| Coaches' Mental Health Literacy and Role Perceptions for Supporting Young People's Mental Health. | Duffy, Jennifer; Rooney, Brendan; Matthews, James | 2021 | Journal of Applied Sport Psychology | 10.1080/10413200.2019.1646840 |
| Collision and Contact Sport Participation and Quality of Life Among Adolescent Athletes. | Howell, David R.; Kirkwood, Michael W.; Laker, Scott; Wilson, Julie C. | 2020 | Journal of Athletic Training (Allen Press) | 10.4085/1062-6050-0536.19 |
| Combinations of physical activity, sedentary time, and sleep duration and their associations with depressive symptoms and other mental health problems in children and adolescents: a systematic review. | Sampasa-Kanyinga, Hugues; Colman, Ian; Goldfield, Gary S.; Janssen, Ian; Wang, JianLi; Podinic, Irina; Tremblay, Mark S.; Saunders, Travis J.; Sampson, Margaret; Chaput, Jean-Philippe | 2020 | International Journal of Behavioral Nutrition & Physical Activity | 10.1186/s12966-020-00976-x |
| Composition Factor Analysis and Factor Invariance of the Physical Appearance State and Trait Anxiety Scale (PASTAS) in Sports and Non-Sports Practitioner Mexican Adolescents. | Ornelas, Martha; Rodríguez-Villalobos, Judith Margarita; Viciana, Jesús; Guedea, Julio César; Blanco, José René; Mayorga-Vega, Daniel | 2021 | Journal of Sports Science & Medicine | 10.52082/jssm.2021.525 |
| CONTACT AND COLLISION SPORTS PARTICIPATION DURING ADOLESCENCE IS ASSOCIATED WITH REDUCED ANXIETY AND DEPRESSIVE SYMPTOMS...Pediatric Research in Sports Medicine Society (PRiSM) 7th Annual Meeting, January 23-25, 2020, Glendale, Arizona | Howell, David R.; Laker, Scott; Kirkwood, Michael W.; Wilson, Julie | 2020 | Orthopaedic Journal of Sports Medicine | 10.1177/2325967120S00169 |
| Correlates of Physical Activity Differ by Sex and Country of Birth Among Mexican-Heritage Youth. | Miller, Erline; Wilkinson, Anna; Koehly, Laura; Daniel, Carrie; Forman, Michele | 2017 | Journal of Immigrant & Minority Health | 10.1007/s10903-016-0451-x |
| Covid-19 Restrictions and the Effects of Physical Activity on Mental Health in Midwestern Adolescent Adaptive Athletes...Pediatric Research in Sports Medicine (PRiSM) 9th Annual Meeting, January 27-29, 2022, Houston, Texas. | Choe, Joshua; Azerf, Saji; Lang, Pamela |  | Orthopaedic Journal of Sports Medicine | 10.1177/2325967121S00507 |
| Cross-sectional and longitudinal association of non-exercise estimated cardiorespiratory fitness with depression and anxiety in the general population: The HUNT study. | Shigdel, Rajesh; Stubbs, Brendon; Sui, Xuemei; Ernstsen, Linda | 2019 | Journal of Affective Disorders | 10.1016/j.jad.2019.04.016 |
| Cumulative Health Risk Behaviors and Adolescent Suicide: The Moderating Role of Future Orientation. | Yunyu Xiao; Wenhua Lu | 2019 | American Journal of Health Behavior | 10.5993/AJHB.43.6.7 |
| Demographic, Injury Characteristics, and Comorbidity Differences Between Youth with >28 Day and >90 Day Recovery from Concussion...Pediatric Research in Sports Medicine (PRiSM) 9th Annual Meeting, January 27-29, 2022, Houston, Texas | Young, Julie A.; Maki, Aaron; Feiss, Robyn; Yang, Jinzhen; Cuff, Steven C. |  | Orthopaedic Journal of Sports Medicine | 10.1177/2325967121S00397 |
| Depression and anxiety in nursing professionals during the covid-19 pandemic. | Rodrigues dos Santos, Katarina Márcia; Rodrigues Galvão, Maria Helena; Marcelino Gomes, Sávio; Araujo de Souza, Talita; de Almeida Medeiros, Arthur; Ribeiro Barbosa, Isabelle |  | Anna Nery School Journal of Nursing / Escola Anna Nery Revista de Enfermagem | 10.1590/2177-9465-EAN-2020-0370 |
| Depression is associated with lower levels of physical activity, body image dissatisfaction, and obesity in Chilean preadolescents. | Delgado-Floody, Pedro; Guzmán-Guzmán, Iris Paola; Caamaño-Navarrete, Felipe; Jerez-Mayorga, Daniel; Zulic-Agramunt, Christianne; Cofré-Lizama, Alfonso | 2021 | Psychology, Health & Medicine | 10.1080/13548506.2020.1817958 |
| Depressive Symptoms Among Urban Adolescents with Asthma: A Focus for Providers. | Shankar, Michelle; Fagnano, Maria; Blaakman, Susan W.; Rhee, Hyekyun; Halterman, Jill S. | 2019 | Academic Pediatrics | 10.1016/j.acap.2018.12.004 |
| Depressive symptoms, perceived stress, self-efficacy, and outcome expectations: Predict fitness among adolescents with obesity. | Tulloch, Heather; Heenan, Adam; Sweet, Shane; Goldfield, Gary S; Kenny, Glen P; Alberga, Angela S; Sigal, Ronald J | 2020 | Journal of Health Psychology | 10.1177/1359105317734039 |
| Determinant Factors of Achievement Motivation in School Physical Education. | García-Ceberino, Juan M.; Feu, Sebastián; Gamero, María G.; Ibáñez, Sergio J. | 2022 | Children | 10.3390/children9091366 |
| Development of a conceptual framework to underpin a health-related quality of life outcome measure in paediatric chronic fatigue syndrome/myalgic encephalopathy (CFS/ME): prioritisation through card ranking. | Parslow, Roxanne M.; Anderson, Nina; Byrne, Danielle; Haywood, Kirstie L.; Shaw, Alison; Crawley, Esther | 2020 | Quality of Life Research | 10.1007/s11136-019-02399-z |
| Diet quality in adolescents with premenstrual syndrome: A cross‐sectional study. | Isgin‐Atici, Kubra; Kanbur, Nuray; Akgül, Sinem; Buyuktuncer, Zehra | 2020 | Nutrition & Dietetics | 10.1111/1747-0080.12515 |
| DIFFERENCES IN QUALITY OF LIFE, SLEEP, AND PHYSICAL ACTIVITY IN ADOLESCENT ATHLETES BEFORE AND DURING COVID-19...Pediatric Research in Sports Medicine (PRiSM), 8th Annual Meeting, 28-30 January, 2021. | Wingerson, Mathew J.; Baugh, Christine M.; Provance, Aaron; Armento, Aubrey; Walker, Gregory; Howell, David R. | 2021 | Orthopaedic Journal of Sports Medicine | 10.1177/2325967121S00152 |
| Do changes in psychosocial factors, lifestyle factors and sitting posture influence the likelihood of musculoskeletal pain in high school computer users? | Brink, Yolandi; Louw, Quinette; Grimmer, Karen | 2020 | Physiotherapy Research International | 10.1002/pri.1865 |
| Do Healthy, Young Adults With Current Feelings Of Anxiety Have Impaired Balance? An Exploratory Study: 1705...American College of Sports Medicine, Annual Meeting and World Congresses, May 31-June 4, 2022, San Diego, California | Dowd, Dalton; Stark, Maggie; Huang, Haikun; Yu, Lap-Fai; Martin, Rebecca; McCarthy, Ryan; Locke, Emily; Yager, Chelsea; Smith, Matthew Lee; Jacobson, Bert; Boolani, Ali | 2022 | Medicine & Science in Sports & Exercise | 10.1249/01.mss.0000880260.52949.ef |
| Do you see what I see? The influence of self-objectification on appearance anxiety, intrinsic motivation, interoceptive awareness, and physical performance. | Dimas, Michelle A.; Galway, Sarah C.; Gammage, Kimberley L. | 2021 | Body Image | 10.1016/j.bodyim.2021.05.010 |
| Does Body Mass Index Influence Behavioral Regulations, Dispositional Flow and Social Physique Anxiety in Exercise Setting? | Ersöz, Gözde; Altiparmak, Ersin; Aşçı, F. Hülya | 2016 | Journal of Sports Science & Medicine |  |
| Does Pilates effect on depression status, pain, functionality, and quality of life in university students? A randomized controlled study. | Saltan, Asuman; Ankaralı, Handan | 2021 | Perspectives in Psychiatric Care | 10.1111/ppc.12547 |
| Early adolescent physical activity, sleep and symptoms of depression at 16 years of age. | Slykerman, Rebecca F.; Thompson, John MD.; Coomarasamy, Christin; Wall, Clare R.; Waldie, Karen E.; Murphy, Rinki; Mitchell, Edwin A. | 2020 | Acta Paediatrica | 10.1111/apa.15140 |
| Ease of walking associates with greater free-living physical activity and reduced depressive symptomology in breast cancer survivors: pilot randomized trial. | Carter, Stephen J.; Hunter, Gary R.; Norian, Lyse A.; Turan, Bulent; Rogers, Laura Q. | 2018 | Supportive Care in Cancer | 10.1007/s00520-017-4015-y |
| Eating disorder mental health literacy: What do psychologists, naturopaths, and fitness instructors know? | Worsfold, Kate A; Sheffield, Jeanie K | 2018 | Eating Disorders | 10.1080/10640266.2017.1397420 |
| Effect of a mindfulness programme for long‐term care residents with type 2 diabetes: A cluster randomised controlled trial measuring outcomes of glycaemic control, relocation stress and depression. | Chen, Shu‐Ming; Lin, Huey‐Shyan; Atherton, John J.; MacIsaac, Richard J.; Wu, Chiung‐Jung (Jo) | 2020 | International Journal of Older People Nursing | 10.1111/opn.12312 |
| Effect of Collaborative Care on Persistent Postconcussive Symptoms in Adolescents: A Randomized Clinical Trial. | McCarty, Carolyn A.; Zatzick, Douglas F.; Marcynyszyn, Lyscha A.; Jin Wang; Hilt, Robert; Jinguji, Thomas; Quitiquit, Celeste; Chrisman, Sara P. D.; Rivara, Frederick P. | 2021 | Journal of Clinical Chiropractic Pediatrics | 10.1001/jamanetworkopen.2021.0207 |
| Effect of Maternal Depression on the Participation of Children With Leukemia in Life Activities. | Khazaeli, Khadije; Khazaeli, Zahra; Jalili, Nasrin; Gharebaghy, Soraya | 2021 | Archives of Rehabilitation | 10.32598/RJ.22.2.3240.1 |
| Effect of Progressive Muscle Relaxation Exercise on Anxiety Reduction Among Nursing Students During Their Initial Clinical Training: A Quasi-Experimental Study. | Toqan, Dalia; Ayed, Ahmad; Joudallah, Hasan; Amoudi, Mosab; Malak, Malakeh Z.; Thultheen, Imad; Batran, Ahmad | 2022 | Inquiry (00469580) | 10.1177/00469580221097425 |
| Effectiveness of Art Therapy on Level of Anxiety among Hospitalized School Age Children in a Selected Hospital at Kanyakumari District. | Maheswari, M.; Evency, A. Reena | 2021 | International Journal of Nursing Education | 10.37506/ijone.v13i4.16584 |
| Effects of a naturally occurring stressor on health behaviors and their psychosocial correlates. | Annesi, James J. | 2020 | Psychology, Health & Medicine | 10.1080/13548506.2019.1658882 |
| Effects of Basketball and Baduanjin Exercise Interventions on Problematic Smartphone Use and Mental Health among College Students: A Randomized Controlled Trial. | Xiao, Tao; Jiao, Can; Yao, Jie; Yang, Lin; Zhang, Yanjie; Liu, Shijie; Grabovac, Igor; Yu, Qian; Kong, Zhaowei; Yu, Jane Jie; Zhang, Jieting | 2021 | Evidence-based Complementary & Alternative Medicine (eCAM) | 10.1155/2021/8880716 |
| Effects of clinical Pilates exercises on cardiovascular endurance and psychosomatic parameters on primary caregivers of special needs children: A randomized controlled trial. | Senturk, Yazgi; Kirmizigil, Berkiye; Tuzun, Emine Handan | 2021 | Journal of Back & Musculoskeletal Rehabilitation | 10.3233/BMR-191649 |
| Effects of COVID-19 pandemic on the psychological states of youth and adult elite male athletes. | Lima, Yavuz; Şenışık, Seçkin; Denerel, Nevzad; Hurşitoğlu, Onur; Balcı, Görkem A.; Bolat, Gül Ü.; Ergün, Metin | 2022 | Spor Hekimligi Dergisi/Turkish Journal of Sports Medicine | 10.47447/tjsm.0597 |
| Effects of Education and Exercise Program for Pain and Functional Capacity on Low Back Pain in Mothers of Children with Cerebral Palsy. | YANIKOĞLU, Sibel MANDIROĞLUa İnci; DELİALİOĞLU, Sibel ÜNSAL; ÇULHA, Canan; DALYAN, Meltem | 2021 | Journal of Physical Medicine & Rehabilitation Sciences | 10.31609/jpmrs.2020-79134 |
| Effects of kettlebell training and detraining on mood status and sleep and life quality of healthy women. | Rufo-Tavares, Weverton; Barbosa Lira, Claudio Andre; Andrade, Marilia Santos; Zimerer, Carla; Leopoldo, André Soares; Sarro, Karine Jacon; Gentil, Paulo; Nikolaidis, Pantelis Theodoros; Rosemann, Thomas; Knechtle, Beat; Vancini, Rodrigo Luiz | 2020 | Journal of Bodywork & Movement Therapies | 10.1016/j.jbmt.2020.07.006 |
| Effects of Rehabilitation Program on Quality of Life, Sleep, Rest-Activity Rhythms, Anxiety, and Depression of Patients With Esophageal Cancer: A Pilot Randomized Controlled Trial. | Chen, Hui-Mei; Lin, Yi-Yun; Wu, Yu-Chung; Huang, Chien-Sheng; Hsu, Po-Kuei; Chien, Ling-I; Lin, Yu-Jung MS,; Huang, Hsiu-Li MS, | 2022 | Cancer Nursing | 10.1097/NCC.0000000000000953 |
| Effects of team sports on anxiety, depression, perceived stress, and sleep quality in college students. | Johnston, Sara Ann; Roskowski, Christine; He, Zhonghui; Kong, Lingchen; Chen, Weiyun | 2021 | Journal of American College Health | 10.1080/07448481.2019.1707836 |
| Effects of Trampoline Exercise on Atentional Control and Daytime Sleepiness among Young Adults with Anxiety Disorders in Malaysia. | Nizar Abdul Majeed Kuty; Mohammed Abdul Razzaq Jabbar; Yip Soon Ving | 2017 | Disability, CBR & Inclusive Development | 10.5463/DCID.v29i4.680 |
| Efficacy of a culturally tailored cognitive-behavioural intervention for Ethiopian children with haematological malignancies: study protocol for randomised controlled trial. | Melesse, Tenaw Gualu; Chau, Janita Pak Chun; Li, William Ho Cheung | 2022 | Trials | 10.1186/s13063-022-06768-x |
| Efficacy of Physiotherapy on Spinal Mobility Parameters and Pain in Persons with Adolescent and Adult Idiopathic Structural Scoliosis. | Balne, Naveen Kumar; Jabeen, S. Afshan; Mathukumalli, Neeharika | 2021 | Indian Journal of Physiotherapy & Occupational Therapy | 10.37506/ijpot.v15i4.16524 |
| Elevated Anxiety and Depression Symptoms in Injured and Uninjured Adolescents...Pediatric Research in Sports Medicine (PRiSM) 9th Annual Meeting, January 27-29, 2022, Houston, Texas | Young, Julie A.; Allison, Ellee; Mohr, Drew; Bonny, Andrea E.; Valasek, Amy E.; Onate, James A. |  | Orthopaedic Journal of Sports Medicine | 10.1177/2325967121S00532 |
| Elevated depression and anxiety symptoms among pregnant individuals during the COVID-19 pandemic. | Lebel, Catherine; MacKinnon, Anna; Bagshawe, Mercedes; Tomfohr-Madsen, Lianne; Giesbrecht, Gerald | 2020 | Journal of Affective Disorders | 10.1016/j.jad.2020.07.126 |
| Evaluation of an exercise physiology service in a youth mental health service. | Pearce, M.; Foote, L.; Brown, E.; O'Donoghue, B.; O'Donoghue, Brian | 2021 | Irish Journal of Psychological Medicine | 10.1017/ipm.2020.91 |
| Evaluation of mood, stress levels and sense of coherence in future physiotherapists. | Kowalska, Joanna; Pawik, Malwina; Wójtowicz, Dorota; Szczepańska-Gieracha, Joanna | 2020 | Work | 10.3233/WOR-203344 |
| Exercise as Treatment for Youth With Major Depression: The Healthy Body Healthy Mind Feasibility Study. | GILES, ADRIANA; NASSTASIA, YASMINA; BAKER, AMANDA; KELLY, BRIAN; DASCOMBE, BEN; HALPIN, SEAN; HIDES, LEANNE; CALLISTER, ROBIN | 2020 | Journal of Psychiatric Practice® | 10.1097/PRA.0000000000000516 |
| Exercise habits and factors associated with exercise in systemic sclerosis: a Scleroderma Patient-centered Intervention Network (SPIN) cohort study. | Azar, Marleine; Rice, Danielle B.; Kwakkenbos, Linda; Carrier, Marie-Eve; Shrier, Ian; Bartlett, Susan J.; Hudson, Marie; Mouthon, Luc; Poiraudeau, Serge; van den Ende, Cornelia H. M.; Johnson, Sindhu R.; Rodriguez Reyna, Tatiana Sofia; Schouffoer, Anne A.; Welling, Joep; Thombs, Brett D.; SPIN investigators | 2018 | Disability & Rehabilitation | 10.1080/09638288.2017.1323023 |
| Factors affecting depression in high school students with chronic illness: A nationwide cross-sectional study in South Korea. | Kim, EunGyeong; Lee, Young-Me; Riesche, Laren | 2020 | Archives of Psychiatric Nursing | 10.1016/j.apnu.2020.01.002 |
| Factors associated with depression and anxiety in children with intellectual disabilities. | Whitney, D. G.; Shapiro, D. N.; Peterson, M. D.; Warschausky, S. A. | 2019 | Journal of Intellectual Disability Research | 10.1111/jir.12583 |
| Factors related to heavy drinking between British Columbia Asian adolescents and South Korean adolescents. | Joung, Kyoung Hwa; Saewyc, Elizabeth M. | 2020 | Journal for Specialists in Pediatric Nursing | 10.1111/jspn.12296 |
| Fear Avoidance Predicts Persistent Pain in Young Adults With Low Back Pain: A Prospective Study. | SMITH, JO ARMOUR; RUSSO, LINDSAY; SANTAYANA, NOEL | 2021 | Journal of Orthopaedic & Sports Physical Therapy | 10.2519/jospt.2021.9828 |
| Fitness, Sleep-Disordered Breathing, Symptoms of Depression, and Cognition in Inactive Overweight Children: Mediation Models. | Stojek, Monika M. K.; Montoya, Amanda K.; Drescher, Christopher F.; Newberry, Andrew; Sultan, Zain; Williams, Celestine F.; Pollock, Norman K.; Davis, Catherine L. |  | Public Health Reports | 10.1177/0033354917731308 |
| From Childbirth to the Starting Blocks: Are We Providing the Best Care to Our Postpartum Athletes? | DEERING, RITA E.; CHRISTOPHER, SHEFALI M.; HEIDERSCHEIT, BRYAN C. | 2020 | Journal of Orthopaedic & Sports Physical Therapy | 10.2519/jospt.2020.0607 |
| Gender differences in the association between functional limitation and depressive symptoms: the salience of food insecurity. | Brown, Robyn Lewis; Ciciurkaite, Gabriele; Imlay, Aimee | 2020 | Women & Health | 10.1080/03630242.2019.1696439 |
| Group activity participation at age 21 and depressive symptoms during boom and recession in Sweden: a 20-year follow-up. | Bean, Christopher G; Virtanen, Marianna; Westerlund, Hugo; Berg, Noora; Hallqvist, Johan; Hammarström, Anne | 2019 | European Journal of Public Health | 10.1093/eurpub/cky201 |
| Group comparison of individuals with and without irritable bowel syndrome in terms of psychological and lifestyle-related factors. | Selvi, Kerim; Bozo, Ozlem | 2022 | Dusunen Adam: Journal of Psychiatry & Neurological Sciences | 10.14744/DAJPNS.2022.00167 |
| Group Intervention for Adolescent Anxiety and Depression: Outcomes of a Randomized Trial with Adolescents in Kenya. | Osborn, Tom L.; Wasil, Akash R.; Venturo-Conerly, Katherine E.; Schleider, Jessica L.; Weisz, John R. | 2020 | Behavior Therapy | 10.1016/j.beth.2019.09.005 |
| Growing Fit Together: Including Social Support in Prenatal and Postnatal Fitness. | Bethany Bonura, Kimberlee | 2017 | International Journal of Childbirth Education |  |
| Gut microbiota composition associated with alterations in cardiorespiratory fitness and psychosocial outcomes among breast cancer survivors. | Paulsen, Jesseca; Ptacek, Travis; Carter, Stephen; Liu, Nianjun; Kumar, Ranjit; Hyndman, LaKeshia; Lefkowitz, Elliot; Morrow, Casey; Rogers, Laura; Paulsen, Jesseca A; Ptacek, Travis S; Carter, Stephen J; Lefkowitz, Elliot J; Morrow, Casey D; Rogers, Laura Q | 2017 | Supportive Care in Cancer | 10.1007/s00520-016-3568-5 |
| Health Conditions, Substance Use, Physical Activity, and Quality of Life in Current and Former Baseball Players. | Bullock, Garrett S.; Nicholson, Kristen F.; Waterman, Brian R.; Niesen, Eric; Salamh, Paul; Thigpen, Charles A.; Shanley, Ellen; Devaney, Laurie; Collins, Gary S.; Arden, Nigel K.; Filbay, Stephanie R. | 2021 | Orthopaedic Journal of Sports Medicine | 10.1177/23259671211056645 |
| Healthcare students' mental and physical well-being during the COVID-19 lockdown and distance learning. | Almhdawi, Khader A.; Alazrai, Alza; Obeidat, Donia; Altarifi, Ahmad A.; Oteir, Alaa O.; Aljammal, Ashraf H.; Arabiat, Alaa A.; Alrabbaie, Hassan; Jaber, Hanan; Almousa, Khaled M. | 2021 | Work | 10.3233/WOR-205309 |
| Heart Rate Variability biofeedback therapy for children and adolescents with chronic pain: A pilot study. | Yetwin, Alexis K.; Mahrer, Nicole E.; Bell, Terece S.; Gold, Jeffrey I. | 2022 | Journal of Pediatric Nursing | 10.1016/j.pedn.2022.06.008 |
| Helping soccer players help themselves: Effectiveness of a psychoeducational book in reducing perfectionism. | Donachie, Tracy C.; Hill, Andrew P. | 2022 | Journal of Applied Sport Psychology | 10.1080/10413200.2020.1819472 |
| How Did Virtual Schooling Affect Youth? | Duerr, Heidi Anne | 2021 | Psychiatric Times |  |
| How Do Hip Exercises Improve Pain in Individuals With Patellofemoral Pain? Secondary Mediation Analysis of Strength and Psychological Factors as Mechanisms. | HOLDEN, SINEAD; MATTHEWS, MARK; RATHLEFF, MICHAEL SKOVDAL; KASZA, JESSICA; VICENZINO, BILL | 2021 | Journal of Orthopaedic & Sports Physical Therapy | 10.2519/jospt.2021.10674 |
| Hypnotherapy or transcendental meditation versus progressive muscle relaxation exercises in the treatment of children with primary headaches: a multi-centre, pragmatic, randomised clinical study. | Jong, M. C.; Boers, I.; van Wietmarschen, H. A.; Tromp, E.; Busari, J. O.; Wennekes, R.; Snoeck, I.; Bekhof, J.; Vlieger, A. M. | 2019 | European Journal of Pediatrics | 10.1007/s00431-018-3270-3 |
| Impact of Covid-19 Pandemic on Paediatric Population: A Case Series. | Shah, Mona A.; Kothari, Vini J.; Chettiar, Rhea Y.; Vengurlekar, Sanjana P.; Shah, Ria C. | 2022 | Indian Practitioner |  |
| Impact of COVID‐19 Pandemic on the Mental Health of Students From 2 Semi‐Rural High Schools in Georgia. | Gazmararian, Julie; Weingart, Rachel; Campbell, Katherine; Cronin, Thomas; Ashta, Jasleen | 2021 | Journal of School Health | 10.1111/josh.13007 |
| Impact of the COVID‐19 pandemic on the physical and psychological health of female college students in Japan. | Washio, Sae; Sai, Akira; Yamauchi, Taro | 2022 | Nursing & Health Sciences | 10.1111/nhs.12962 |
| Implementation in action: how Australian Exercise Physiologists approach exercise prescription for people with mental illness. | Stanton, Robert; Rosenbaum, Simon; Lederman, Oscar; Happell, Brenda | 2018 | Journal of Mental Health | 10.1080/09638237.2017.1340627 |
| Implications of Psychological Morbidity on Physical Health and Behavior of Adolescents in Aligarh, India. | Faizi, Nafis; Shah, Mohammad Salman; Ahmad, Anees; Azmi, Suhail Ahmad; Maroof, Mohd; Khalique, Najam | 2018 | Journal of Pediatrics Review | 10.5812/jpr.11083 |
| Improvements in well-being and cardiac metrics of stress following a yogic breathing workshop: Randomized controlled trial with active comparison. | Goldstein, Michael R.; Lewin, Rivian K.; Allen, John J. B. | 2022 | Journal of American College Health | 10.1080/07448481.2020.1781867 |
| Influence of Anxiety Sensitivity and Negative Affect on Concussion Outcomes...Pediatric Research in Sports Medicine (PRiSM) 9th Annual Meeting, January 27-29, 2022, Houston, Texas. | Caze II, Todd; Williams, Kimberly; Boucher, Sarah; Price, August; Abt, John; Burkhart, Scott |  | Orthopaedic Journal of Sports Medicine | 10.1177/2325967121S00424 |
| Influence of grit and healthy lifestyle behaviors on anxiety and depression in US adults at the beginning of the COVID-19 pandemic: Cross-sectional study. | Toczko, Mike; Merrigan, Justin; Boolani, Ali; Guempel, Bishop; Milani, Italia; Martin, Joel | 2022 | Health Promotion Perspectives |  |
| Ingesting a Post-Workout Vegan-Protein Multi-Ingredient Expedites Recovery after Resistance Training in Trained Young Males. | Naclerio, PhD, Fernando; Seijo, PhD, Marcos; Earnest , PhD, Conrad P.; Puente-Fernández, MSc, Joel; Larumbe-Zabala, PhD, Eneko | 2021 | Journal of Dietary Supplements | 10.1080/19390211.2020.1832640 |
| INITIAL CLINICAL PRESENTATION OF PEDIATRIC PATIENTS WITH PSYCHOLOGICAL DISORDERS FOLLOWING CONCUSSION...Pediatric Research in Sports Medicine (PRiSM), 8th Annual Meeting, 28-30 January, 2021. | Worrall, Hannah M.; Miller, Shane M.; Cullum, C. Munro; Chung, Jane S. | 2021 | Orthopaedic Journal of Sports Medicine | 10.1177/2325967121S00080 |
| Integrating Diabetes Prevention Education Among Teenagers Involved in Summer Employment: Encouraging Environments for Health in Adolescence (ENHANCE). | Yazel-Smith, Lisa; El-Mikati, Hala K.; Adjei, Michael; Haberlin-Pittz, Kathryn M.; Agnew, Megan; Hannon, Tamara S. | 2020 | Journal of Community Health | 10.1007/s10900-020-00802-2 |
| Interaction of substance use with physical activity and its effect on depressive symptoms among adolescents. | Chauhan, Shekhar; Srivastava, Shobhit; Kumar, Pradeep; Patel, Ratna; T., Muhammad; Dhillon, Preeti | 2021 | Journal of Substance Use | 10.1080/14659891.2020.1851411 |
| Interventions Within the Scope of Occupational Therapy to Address Preventable Adverse Events in Inpatient and Home Health Postacute Care Settings: A Systematic Review. | Hunter, Elizabeth G.; Rhodus, Elizabeth | 2022 | American Journal of Occupational Therapy | 10.5014/ajot.2022.047589 |
| Intra‐and Interpersonal Factors Buffer the Relationship Between Food Insecurity and Mental Well‐Being Among Middle Schoolers. | Brinkman, Jesse; Garnett, Bernice; Kolodinsky, Jane; Wang, Weiwei; Pope, Lizzy | 2021 | Journal of School Health | 10.1111/josh.12982 |
| Invasive diagnostic and therapeutic measures are unnecessary in patients with symptomatic van Neck–Odelberg disease (ischiopubic synchondrosis): a retrospective single-center study of 21 patients with median follow-up of 5 years. | Schneider, Kristian Nikolaus; Lampe, Lukas Peter; Gosheger, Georg; Theil, Christoph; Masthoff, Max; Rödl, Robert; Vogt, Björn; Andreou, Dimosthenis | 2021 | Acta Orthopaedica | 10.1080/17453674.2021.1882237 |
| Is replacing time spent in 1 type of physical activity with another associated with health in children? | Macgregor, Anne P.; Borghese, Michael M.; Janssen, Ian | 2019 | Applied Physiology, Nutrition & Metabolism | 10.1139/apnm-2018-0323 |
| Leisure practice in adolescents and associated factors: implications for care. | Farias Marcino, Lethícia; Ciccone Giacon-Arruda, Bianca Cristina; Ferraz Teston, Elen; Schiaveto de Souza, Albert; Marcheti, Priscila Maria; de Pádua Lima, Helder; Silva Marcon, Sonia; Aratani, Nathan | 2022 | Acta Paulista de Enfermagem | 10.37689/acta-ape/2022AO02041 |
| Leisure time physical activity and depressive symptoms among adolescents in Sweden. | Ma, Li; Hagquist, Curt; Kleppang, Annette Løvheim | 2020 | BMC Public Health | 10.1186/s12889-020-09022-8 |
| Leisure-time physical activity is negatively associated with depression symptoms independently of the socioeconomic status. | Marques, Adilson; Peralta, Miguel; Gouveia, Élvio R.; Martins, João; Sarmento, Hugo; Gomez-Baya, Diego | 2020 | European Journal of Sport Science | 10.1080/17461391.2019.1701716 |
| Lessons Learned from the Impact of Adolescents' Internet Use Disorders on Adolescents' Substance Use Disorders. | Randall, Jeff; York, Janet A. | 2020 | Journal of Child & Adolescent Substance Abuse | 10.1080/1067828X.2021.1967247 |
| Life after lockdown: The role of sport, exercise and physical activity in ameliorating the mental health implications of COVID-19 restrictions. | Richardson, Darren L.; Clarke, Neil D.; Broom, David R.; Tallis, Jason; Duncan, Michael J. | 2021 | Journal of Sports Sciences | 10.1080/02640414.2021.1923186 |
| Living with hypoparathyroidism: development of the Hypoparathyroidism Patient Experience Scale-Impact (HPES-Impact). | Brod, Meryl; Waldman, Laura Tesler; Smith, Alden; Karpf, David | 2021 | Quality of Life Research | 10.1007/s11136-020-02607-1 |
| Longitudinal evaluation of the psychological impact of the COVID-19 crisis in Spain. | Planchuelo-Gómez, Álvaro; Odriozola-González, Paula; Irurtia, María Jesús; de Luis-García, Rodrigo | 2020 | Journal of Affective Disorders | 10.1016/j.jad.2020.09.018 |
| Maternal and perinatal parameters after non-pharmacological interventions: a randomised, controlled clinical trial. | de Souza Melo, Patrícia; Barbieri, Márcia; Westphal, Flavia; Fustinoni, Suzete Maria; Henrique, Angelita José; Amorim Francisco, Adriana; Gabrielloni, Maria Cristina | 2020 | Acta Paulista de Enfermagem | 10.37689/actaape/2020AO0136 |
| Maternal Characteristics that Impact Postpartum Weight Retention: Results from the 2016 Los Angeles Mommy and Baby (LAMB) Follow-Up Study. | Archuleta, Jennifer; Chao, Shin Margaret | 2021 | Maternal & Child Health Journal | 10.1007/s10995-020-03082-3 |
| ME/CFS in Adolescents -- Study Review. | Szrajda, Justyna | 2020 | Journal of Neurological & Neurosurgical Nursing | 10.15225/PNN.2020.9.2.5 |
| Media, Technology Use, and Attitudes: Associations With Physical and Mental Well-Being in Youth With Implications for Evidence-Based Practice. | Zeeni, Nadine; Doumit, Rita; Abi Kharma, Joelle; Sanchez‐Ruiz, Maria‐Jose | 2018 | Worldviews on Evidence-Based Nursing | 10.1111/wvn.12298 |
| Mental Health During Late Pregnancy and Postpartum in Mothers With and Without Type 1 Diabetes: The ENDIA Study. | Hall, Madeleine; Oakey, Helena; Penno, Megan A.S.; McGorm, Kelly; Anderson, Amanda J.; Ashwood, Pat; Colman, Peter G.; Craig, Maria E.; Davis, Elizabeth A.; Harris, Mark; Harrison, Leonard C.; Haynes, Aveni; Morbey, Claire; Sinnott, Richard O.; Soldatos, Georgia; Vuillermin, Peter J.; Wentworth, John M.; Thomson, Rebecca L.; Couper, Jennifer J.; HarrisMarkHarrisonLeonard C.HaynesAveniKimKi WookMorahanGrantOakeyHelenaPennoMegan A.S.RawlinsonWilliam D.SinnottRichard O.SoldatosGeorgiaThomsonRebecca L.VuillerminPeter J.WentworthJohn M.AndersonAmanda J.AshwoodPatBrownJames D.HuWilliamHuynhDaoMcGormKel | 2022 | Diabetes Care | 10.2337/dc21-2335 |
| Mental Health, Physical Activity, and Quality of Life of US Adolescent Athletes During COVID-19–Related School Closures and Sport Cancellations: A Study of 13 000 Athletes. | McGuine, Timothy A.; Biese, Kevin M.; Petrovska, Labina; Hetzel, Scott J.; Reardon, Claudia; Kliethermes, Stephanie; Bell, David R.; Brooks, Alison; Watson, Andrew M. | 2021 | Journal of Athletic Training (Allen Press) | 10.4085/1062-6050-0478.20 |
| Mexican‐American adolescents' perceptions about causes of perinatal depression, self‐help strategies, and how to obtain mental health information. | Recto, Pamela; Dimmitt Champion, Jane | 2018 | Journal of Child & Adolescent Psychiatric Nursing | 10.1111/jcap.12210 |
| Minding many minds: An assessment of mental health and resilience among undergraduate and graduate students; a mixed methods exploratory study. | Fried, Rebecca R.; Karmali, Shazya; Irwin, Jennifer D. | 2022 | Journal of American College Health | 10.1080/07448481.2020.1781134 |
| Modelling of South African Hypertension: Comparative Analysis of the Classical and Bayesian Quantile Regression Approaches. | Kuhudzai, Anesu Gelfand; Van Hal, Guido; Van Dongen, Stefan; Hoque, Muhammad | 2022 | Inquiry (00469580) | 10.1177/00469580221082356 |
| Mood-related changes in children and adolescents with persistent concussion symptoms following a six-week active rehabilitation program. | Hunt, Anne W.; Agnihotri, Sabrina; Sack, Leah; Tint, Ami; Greenspoon, Dayna; Gauvin-Lepage, Jerome; Gagnon, Isabelle; Reed, Nick; Scratch, Shannon | 2020 | Brain Injury | 10.1080/02699052.2020.1776396 |
| Mothers' views of health problems in the 12 months after childbirth: A concept mapping study. | Rouhi, Maryam; Stirling, Christine M.; Crisp, Elaine P. | 2019 | Journal of Advanced Nursing (John Wiley & Sons, Inc.) | 10.1111/jan.14187 |
| MsFLASH analysis of diurnal salivary cortisol and palpitations in peri- and postmenopausal women. | Carpenter, Janet S. FAAN; Tisdale, James E. PharmD; Larson, Joseph C. MS; Sheng, Ying; Chen, Chen X.; Von Ah, Diane; Kovacs, Richard; Reed, Susan D.; Thurston, Rebecca C.; Guthrie, Katherine A.; Carpenter, Janet S; Tisdale, James E; Larson, Joseph C | 2022 | Menopause (10723714) | 10.1097/GME.0000000000001897 |
| Musculoskeletal Pain and its Risk Factors Among School-Going Adolescents in Delhi, India. | Kanjilal, Maumita; Kumar, Uma; Gupta, Gajendra Kumar; Agrawal, Deepika; Arya, Ravi Kant; Dhakar, Jagmohan Singh | 2020 | Online Journal of Health & Allied Sciences |  |
| Music, Movement, and Mind: Use of Drumming to Improve Strength, Balance, Proprioception, Stamina, Coordination, and Emotional Status in a 12-Year-Old With Agenesis of the Corpus Callosum: A Case Study. | Spak, David; Card, Elizabeth | 2020 | Journal of Holistic Nursing | 10.1177/0898010119871380 |
| New Research Supports Use of a Prenatal Case Management-Style Intervention. |  | 2022 | Hospital Case Management |  |
| Non-medical interventions for depression in children and adolescents. | Freshwater, Edward | 2016 | Mental Health Practice | 10.7748/mhp.2016.e1115 |
| Non-Substance Addiction in Childhood and Adolescence: The Internet, Computer Games and Social Media. | Geisel, Olga; Lipinski, Anneke; Kaess, Michael | 2021 | Deutsches Ärzteblatt International | 10.3238/arztebl.m2021.0002 |
| Occupational Performance Issues of Adults Seeking Bariatric Surgery for Obesity. | Barclay, Karen S.; Forwell, Susan J. | 2018 | American Journal of Occupational Therapy | 10.5014/ajot.2018.025924 |
| Organized Extracurricular Activities: Are In-School and Out-of-School Activities Associated With Different Outcomes for Canadian Youth? | Guèvremont, Anne; Findlay, Leanne; Kohen, Dafna | 2014 | Journal of School Health | 10.1111/josh.12154 |
| Outcomes of Camp Participation for Youth With Cancer. | King, Joanna; Porter, Heather R. | 2016 | Therapeutic Recreation Journal | 10.18666/TRJ-2016-V50-I4-7636 |
| Pacific Islands Cohort on Cardiometabolic Health Study: rationale and design. | Guerrero, Rachael T. Leon; Hattori-Uchima, Margaret P.; Badowski, Grazyna; Aflague, Tanisha F.; Wood, Kathryn; Hammond, Kristi; Perez, Remedios | 2022 | BMC Public Health | 10.1186/s12889-022-13783-9 |
| Pain management in the first stage of labour using sensory stimulation. | Mujiyani, Surya Andina; Latifah, Lutfatul | 2022 | British Journal of Midwifery | 10.12968/bjom.2022.30.7.396 |
| Parental Factors Related to Physical Activity among Adolescent Men Living in Built and Natural Environment: A Population-Based MOPO Study. | Pyky, Riitta; Puhakka, Soile; Ikäheimo, Tiina M.; Lankila, Tiina; Kangas, Maarit; Mäntysaari, Matti; Jämsä, Timo; Korpelainen, Raija | 2021 | Journal of Environmental & Public Health | 10.1155/2021/3234083 |
| Patient Reported Outcome Measures in Pediatric Anterior Cruciate Ligament Reconstruction: Does Promis Correlate with Pedi-Ikdc?...FUGT 07/02/2022 - Orthopaedic Journal of Sports Medicine | Williams, Brendan A.; Johnson, Mitchell; Pridgen, Eric; McNeely, Lia W.; Ganley, Theodore J. |  | Orthopaedic Journal of Sports Medicine | 10.1177/2325967121S00429 |
| Pediatric Obesity in Primary Practice: A Review of the Literature. | Durbin, Jessica | 2018 | Pediatric Nursing |  |
| Peer Crowd Identification and Adolescent Health Behaviors: Results From a Statewide Representative Study. | Jordan, Jeffrey W.; Stalgaitis, Carolyn A.; Charles, John; Madden, Patrick A.; Radhakrishnan, Anjana G.; Saggese, Daniel | 2019 | Health Education & Behavior | 10.1177/1090198118759148 |
| Perceived stress, wellbeing and mental health of college student during COVID-19 pandemic. | Khadirnavar, Afreenbanu A.; Vanageri, Husenasab; Asagi, Renuka E. | 2020 | Indian Journal of Psychiatric Social Work | 10.29120/IJPSW.2020.v11.i2.231 |
| Physical Activity Among Adults With Autism: Participation, Attitudes, and Barriers. | Hillier, Ashleigh; Buckingham, Abigail; Schena II, David | 2020 | Perceptual & Motor Skills | 10.1177/0031512520927560 |
| Physical activity and analogue anxiety disorder symptoms and status: Mediating influence of social physique anxiety. | Herring, Matthew P.; Gordon, Brett R.; McDowell, Cillian P.; Quinn, Leanne M.; Lyons, Mark | 2021 | Journal of Affective Disorders | 10.1016/j.jad.2020.12.163 |
| Physical activity and cardiovascular health in depression: Links between changes in physical activity and cardiovascular risk. | Kim, Hyewon; Yoo, Juhwan; Han, Kyungdo; Jeon, Hong Jin | 2022 | General Hospital Psychiatry | 10.1016/j.genhosppsych.2022.07.002 |
| Physical Activity and Child Depressive Symptoms: Findings From the QLSCD. | Piché, Geneviève; Huỳnh, Christophe; Villatte, Aude | 2019 | Canadian Journal of Behavioural Science | 10.1037/cbs0000120 |
| Physical Activity and Depression during Pregnancy: A Scoping Review. | Abdullah, Ain Widad; Bee Suan Wee; Yahaya, Rosliza; Mohamad, Marhazlina; Shahril, Mohd Razif; Pei Lin Lua | 2021 | International Medical Journal |  |
| Physical activity and mental health in children and adolescents: An updated review of reviews and an analysis of causality. | Biddle, Stuart J.H.; Ciaccioni, Simone; Thomas, George; Vergeer, Ineke | 2019 | Psychology of Sport & Exercise | 10.1016/j.psychsport.2018.08.011 |
| Physical activity and physical fitness as protective factors of adolescent health. | Lukács, Andrea; Sasvári, Péter; Kiss-Tóth, Emőke | 2020 | International Journal of Adolescent Medicine & Health | 10.1515/ijamh-2018-0017 |
| Physical Activity and School Absenteeism Due to Illness in Adolescents. | de Groot, Renate; van Dijk, Martin; Savelberg, Hans; van Acker, Frederik; Kirschner, Paul | 2017 | Journal of School Health | 10.1111/josh.12542 |
| Physical activity correlates across the lifespan in people with epilepsy: a systematic review. | Vancampfort, Davy; Ward, Philip B. | 2021 | Disability & Rehabilitation | 10.1080/09638288.2019.1665113 |
| Physical activity in early adolescence predicts depressive symptoms 3 years later: A community-based study. | Isaksson, Johan; Selinus, Eva Noren; Åslund, Cecilia; Nilsson, Kent W | 2020 | Journal of Affective Disorders | 10.1016/j.jad.2020.09.008 |
| Physical activity in European adolescents and associations with anxiety, depression and well-being. | McMahon, Elaine; Corcoran, Paul; O'Regan, Grace; Keeley, Helen; Cannon, Mary; Carli, Vladimir; Wasserman, Camilla; Hadlaczky, Gergö; Sarchiapone, Marco; Apter, Alan; Balazs, Judit; Balint, Maria; Bobes, Julio; Brunner, Romuald; Cozman, Doina; Haring, Christian; Iosue, Miriam; Kaess, Michael; Kahn, Jean-Pierre; Nemes, Bogdan | 2017 | European Child & Adolescent Psychiatry | 10.1007/s00787-016-0875-9 |
| Physical Activity of Secondary School Adolescents at Risk of Depressive Symptoms. | Frömel, Karel; Jakubec, Lukáš; Groffik, Dorota; ChmelÍk, František; Svozil, Zbyněk; Šafář, Michal | 2020 | Journal of School Health | 10.1111/josh.12911 |
| Physical activity, pain interference and comorbidities relate to PROMIS physical function in younger adults following total knee arthroplasty. | Christensen, Jesse; Peters, Christopher; Gililland, Jeremy; Stoddard, Gregory; Pelt, Christopher | 2021 | Disability & Rehabilitation | 10.1080/09638288.2020.1749944 |
| Physical inactivity and symptoms of depression, anxiety and stress in adolescent students. | Costa, Marcos Paulo da Silva; Schmidt, Ademir; de de Oliveira Vitorino, Priscila Valver; de Sousa Corrêa, Krislainy | 2021 | Acta Paulista de Enfermagem | 10.37689/actaape/2021AO03364 |
| Physically Isolated but Socially Connected: Psychological Adjustment and Stress Among Adolescents During the Initial COVID-19 Crisis. | Ellis, Wendy E.; Dumas, Tara M.; Forbes, Lindsey M. | 2020 | Canadian Journal of Behavioural Science | 10.1037/cbs0000215 |
| Pilot study of the Sub-Symptom Threshold Exercise Program (SSTEP) for persistent concussion symptoms in youth. | Chrisman, Sara P. D.; Whitlock, Kathryn B.; Somers, Elissa; Burton, Monique S.; Herring, Stanley A.; Rowhani-Rahbar, Ali; Rivara, Frederick P. | 2017 | NeuroRehabilitation | 10.3233/NRE-161436 |
| Playing High School Football Is Not Associated With an Increased Risk for Suicidality in Early Adulthood. | Iverson, Grant L.; Merz, Zachary C.; Terry, Douglas P. | 2021 | Clinical Journal of Sport Medicine | 10.1097/JSM.0000000000000890 |
| Post-acute COVID-19 Syndrome Negatively Impacts Physical Function, Cognitive Function, Health-Related Quality of Life, and Participation. | Tabacof, Laura; Tosto-Mancuso, Jenna D; Wood, Jamie; Cortes, Mar; Kontorovich, Amy; McCarthy, Dayna DO; Rizk, Dahlia; Rozanski, Gabriela; Breyman, Erica; Nasr, Leila; Kellner, Christopher; Herrera, Joseph E. DO FAAPMR; Putrino, David | 2022 | American Journal of Physical Medicine & Rehabilitation | 10.1097/PHM.0000000000001910 |
| POST-CONCUSSION DIZZINESS SEVERITY PREDICTS DAILY STEP COUNT DURING RECOVERY AMONG ADOLESCENT ATHLETES...Pediatric Research in Sports Medicine (PRiSM) 9th Annual Meeting, January 27-29, 2022, Houston, Texas | Smulligan, Katherine L.; Seehusen, Corrine N.; Wingerson, Mathew J.; Wilson, Julie C.; Howell, David. R |  | Orthopaedic Journal of Sports Medicine | 10.1177/2325967121S00405 |
| Postural orthostatic tachycardia syndrome (POTS) in teens: A guide for behavior change to manage symptoms. | Cutitta, Katherine E.; Self, Mariella; la Uz, Caridad | 2019 | Pacing & Clinical Electrophysiology | 10.1111/pace.13571 |
| Predictors of parenting self‐agency among mothers receiving substance abuse or mental health treatment. | Matsuda, Yui; Kim, Young‐Ju; Salani, Deborah A.; McCabe, Brian E.; Mitrani, Victoria Behar | 2019 | International Journal of Mental Health Nursing | 10.1111/inm.12624 |
| Pre-post analysis of a social capital-based exercise adherence intervention for breast cancer survivors with moderate fatigue: a randomized controlled trial. | Kim, Sue; Ko, Yun Hee; Song, Yoonkyung; Kang, Min Jae; Lee, Hyojin; Kim, Sung Hae; Jeon, Justin Y.; Cho, Young Up; Yi, Gihong; Han, Jeehee | 2020 | Supportive Care in Cancer | 10.1007/s00520-020-05363-7 |
| Prescribed exercise for the treatment of depression in a college population: An interprofessional approach. | Yates, Brittany Evans; DeLetter, Mary C.; Parrish, Evelyn M. | 2020 | Perspectives in Psychiatric Care | 10.1111/ppc.12508 |
| Presence and Perceptions of Menstrual Dysfunction Among High School Female Athletes...Pediatric Research in Sports Medicine Society (PRiSM) 7th Annual Meeting, January 23-25, 2020, Glendale, Arizona | Armento, Aubrey; VanBaak, Karin; Sweeney, Emily A.; Wilson, Julie C.; Howell, David R. | 2020 | Orthopaedic Journal of Sports Medicine | 10.1177/2325967120S00168 |
| Prevalence and factors associated with depression, anxiety, and stress symptoms among older adults: A cross‐sectional population‐based study. | Thapa, Deependra K.; Visentin, Denis C.; Kornhaber, Rachel; Cleary, Michelle | 2020 | Nursing & Health Sciences | 10.1111/nhs.12783 |
| Prevalence and relevant factors of anxiety and depression among pregnant women in a cohort study from south-east China. | Zhang, Youding; Muyiduli, Xiamusiye; Wang, Shuojia; Jiang, Wen; Wu, Jinhua; Li, Minchao; Mo, Minjia; Jiang, Shuying; Wang, Zhaopin; Shao, Bule; Shen, Yu; Yu, Yunxian | 2018 | Journal of Reproductive & Infant Psychology | 10.1080/02646838.2018.1492098 |
| Prevalence of Biopsychosocial Factors of Pain in 865 Sports Students of the Dach (Germany, Austria, Switzerland) Region -- A Cross-Sectional Survey. | Bumann, Anke; Banzer, Winfried; Fleckenstein, Johannes | 2020 | Journal of Sports Science & Medicine |  |
| Prevalence, characteristics, and treatment of fatigue in oncological cancer patients in Italy: a cross-sectional study of the Italian Network for Supportive Care in Cancer (NICSO). | Roila, Fausto; Fumi, Guglielmo; Ruggeri, Benedetta; Antonuzzo, Andrea; Ripamonti, Carla; Fatigoni, Sonia; Cavanna, Luigi; Gori, Stefania; Fabi, Alessandra; Marzano, Nicola; Graiff, Claudio; De Sanctis, Vitaliana; Mirabile, Aurora; Serpentini, Samantha; Bocci, Chiara; Pino, Maria Simona; Cilenti, Giuseppina; Verusio, Claudio; Ballatori, Enzo; on behalf of NICSO (Network Italiano per le Cure di Supporto in Oncologia) | 2019 | Supportive Care in Cancer | 10.1007/s00520-018-4393-9 |
| Prospective Associations between Sport Participation and Indices of Mental Health across Adolescence. | Graupensperger, Scott; Sutcliffe, Jordan; Vella, Stewart A. | 2021 | Journal of Youth & Adolescence | 10.1007/s10964-021-01416-0 |
| Psychological Benefits of Physical Activity in Children with Mental Health Disorders. | O'Connell, Janelle K.; O'Connell, Dennis; O'Connell, Keelan K. | 2020 | International Journal of Child Development & Mental Health |  |
| Psychological Distress Differs Between Female and Male College Athletes During Baseline Concussion Assessment. | Roby, Patricia R.; Ford, Cassie B.; Wasserman, Erin B.; Rodrigo, Corey J.; DeFreese, J. D.; McCrea, Michael; Mihalik, Jason P. | 2021 | Athletic Training & Sports Health Care: The Journal for the Practicing Clinician | 10.3928/19425864-20210228-02 |
| Psychological factors associated with the use of weight management behaviours in young adults. | Mulgrew, Kate E.; Kannis-Dymand, Lee; Hughes, Emily; Carter, Janet D.; Kaye, Sherrie | 2019 | Journal of Health Psychology | 10.1177/1359105316675210 |
| Psychometric Properties and Factor Structure of the Center for Epidemiologic Studies Depression Scale in a National Sample of Arab Adolescents. | Dardas, Latefa Ali; Xu, Hanzhang; Shawashreh, Atef; Franklin, Michelle S.; Wainwright, Kristin; Pan, Wei | 2019 | Issues in Mental Health Nursing | 10.1080/01612840.2018.1534912 |
| Psychosocial Mechanism of Adolescents' Depression: A Dose-Response Relation with Physical Activity. | Man Xiang; Xiangli Gu; Xiaoxia Zhang; Moss, Samantha; Chaoqun Huang; Nelson, Larry Paul; Tao Zhang | 2020 | Children | 10.3390/children7040037 |
| Psychosocial, behavioral and clinical correlates of children with overweight and obesity. | Thaker, Vidhu V.; Osganian, Stavroula K.; deFerranti, Sarah D.; Sonneville, Kendrin R.; Cheng, Jennifer K.; Feldman, Henry A.; Richmond, Tracy K. | 2020 | BMC Pediatrics | 10.1186/s12887-020-02145-2 |
| Quality of life in people with epidermolysis bullosa: a systematic review. | Togo, C. C. G.; Zidorio, A. P. C.; Gonçalves, V. S. S.; Hubbard, L.; de Carvalho, K. M. B.; Dutra, E. S. | 2020 | Quality of Life Research | 10.1007/s11136-020-02495-5 |
| Relationship between neighbourhood social participation and depression among older adults: A longitudinal study in China. | Wang, Ruoyu; Feng, Zhixin; Liu, Ye; Lu, Yi | 2020 | Health & Social Care in the Community | 10.1111/hsc.12859 |
| RELATIONSHIP BETWEEN PHYSICAL ACTIVITY LEVEL AND DEPRESSION, ANXIETY, QUALITY OF LIFE, SELFESTEEM, AND HBA1C IN ADOLESCENTS WITH TYPE 1 DIABETES MELLITUS. | KAYA MUTLU, Ebru; MUTLU, Caner; TAŞKIRAN, Hanifegül; ÖZGEN, İlker Tolga | 2017 | Turkish Journal of Physiotherapy Rehabilitation |  |
| Relationship between sleep and exercise as colorectal cancer survivors transition off treatment. | Coles, Theresa; Bennett, Antonia V.; Tan, Xianming; Battaglini, Claudio L.; Sanoff, Hanna K.; Basch, Ethan; Jensen, Roxanne E.; Reeve, Bryce B. | 2018 | Supportive Care in Cancer | 10.1007/s00520-018-4110-8 |
| Relationship of childhood maltreatment, exercise, and emotion regulation to self-esteem, PTSD, and depression symptoms among college students. | Fasciano, Laura C.; Dale, Lourdes P.; Shaikh, Samia K.; Little Hodge, Alicia L.; Gracia, Brittney; Majdick, Jennifer M.; Holder, Alanah Y.; Ford, Julian D. | 2021 | Journal of American College Health | 10.1080/07448481.2019.1705837 |
| Relationship of Circadian Rhythm and Psychological Health in Adolescents and Young Adults With Cancer. | Li, Lijun; Duan, Yinglong; Sun, Qian; Xiao, Panpan; Wang, Lu; He, Shiwen; Liu, Xiangyu; Zhou, Jianda; Xie, Jianfei; Cheng, Andy S.K. | 2021 | Cancer Nursing | 10.1097/NCC.0000000000000971 |
| Relationships among Executive Function, Cognitive Load, and Weight-related Behaviors in University Students. | Byrd-Bredbenner, Carol; Eck, Kaitlyn M. | 2020 | American Journal of Health Behavior | 10.5993/AJHB.44.5.12 |
| Relationships Among Exercise, Mindfulness, Mental Health, and Academic Achievement Among Prelicensure Nursing Students. | Niedermeier, Josie; Mumba, Mercy Ngosa; Barron, Keri; Andrabi, Mudasir; Martin, Rebecca; McDiarmid, Alex | 2022 | Nurse Educator | 10.1097/NNE.0000000000001106 |
| Research priorities for adolescent health in low- and middle-income countries: A mixed-methods synthesis of two separate exercises. | Nagata, Jason M.; Hathi, Sejal; Ferguson, B. Jane; Hindin, Michele J.; Sachiyo Yoshida; Ross, David A.; Yoshida, Sachiyo | 2018 | Journal of Global Health | 10.7189/jogh.08.010501 |
| Resistance exercise training among young adults with analogue generalized anxiety disorder. | Gordon, Brett R.; McDowell, Cillian P.; Lyons, Mark; Herring, Matthew P. | 2021 | Journal of Affective Disorders | 10.1016/j.jad.2020.12.020 |
| Review of the unprecedented impact of the COVID-19 pandemic on the occurrence of eating disorders. | Meier, Karien; van Hoeken, Daphne; Hoek, Hans W. | 2022 | Current Opinion in Psychiatry | 10.1097/YCO.0000000000000815 |
| Risk and protective factors associated with depressive symptoms in young adults with overweight and obesity. | Kaufman, Caroline C.; Thurston, Idia B.; Maclin-Akinyemi, Courtney; Hardin, Robin N.; Decker, Kristina M.; Kamody, Rebecca C. | 2020 | Journal of American College Health | 10.1080/07448481.2018.1536057 |
| Risk Factors Related to Binge Drinking: A Comparative Study of American and South Korean Adolescents. | Chung, Sung Suk; Joung, Kyoung Hwa | 2019 | Journal of School Nursing | 10.1177/1059840518785439 |
| Risk-Taking Behaviors in Iranian Children and Adolescents: A Latent Class Analysis Approach: Caspian IV Study. | Abbasi-Ghahramanloo, Abbas; Heshmat, Ramin; Safiri, Saeid; Motlagh, Mohammad Esmaeil; Ardalan, Gelayol; Mahdavi-Gorabi, Armita; Asayesh, Hamid; Qorbani, Mostafa; Kelishadi, Roya | 2018 | Journal of Research in Health Sciences |  |
| Role of Physical Activity and Sedentary Behavior in the Mental Health of Preschoolers, Children and Adolescents: A Systematic Review and Meta-Analysis. | Rodriguez-Ayllon, María; Cadenas-Sánchez, Cristina; Estévez-López, Fernando; Muñoz, Nicolas E.; Mora-Gonzalez, Jose; Migueles, Jairo H.; Molina-García, Pablo; Henriksson, Hanna; Mena-Molina, Alejandra; Martínez-Vizcaíno, Vicente; Catena, Andrés; Löf, Marie; Erickson, Kirk I.; Lubans, David R.; Ortega, Francisco B.; Esteban-Cornejo, Irene | 2019 | Sports Medicine | 10.1007/s40279-019-01099-5 |
| role of sport in coping and resilience amongst resettled South Sudanese youth in Australia. | Pittaway, Troy; Dantas, Jaya A R | 2022 | Health Promotion International | 10.1093/heapro/daab100 |
| School-related physical activity interventions and mental health among children: a systematic review and meta-analysis. | Andermo, Susanne; Hallgren, Mats; Nguyen, Thi-Thuy-Dung; Jonsson, Sofie; Petersen, Solveig; Friberg, Marita; Romqvist, Anja; Stubbs, Brendon; Elinder, Liselotte Schäfer | 2020 | Sports Medicine - Open | 10.1186/s40798-020-00254-x |
| Screening for childhood adversity: the what and when of identifying individuals at risk for lifespan health disparities. | Kuhlman, Kate Ryan; Robles, Theodore F.; Bower, Julienne E.; Carroll, Judith E. | 2018 | Journal of Behavioral Medicine | 10.1007/s10865-018-9921-z |
| Self-Reported Amnesia Following Concussion Predicts Greater Deficits in Quality of Life Domains Among Pediatric Athletes...Pediatric Research in Sports Medicine (PRiSM) 9th Annual Meeting, January 27-29, 2022, Houston, Texas | McLeod, Tamara Valovich; Snedden, Traci; Halstead, Mark; Wilson, Julie; Master, Christina; Grady, Matthew; Fazekas, Matt; Santana, Jonathan; Zaslow, Tracy; Miller, Shane; Howell, David |  | Orthopaedic Journal of Sports Medicine | 10.1177/2325967121S00404 |
| Self-Reported Rapid Eye Movement Sleep Behavior Disturbance and Its Associated Factors among Medicine and Health Science Students at the University of Gondar. | Dagnew, Baye; Dagne, Henok; Andualem, Zewudu | 2020 | BioMed Research International | 10.1155/2020/1810836 |
| Set up for success. | GREEN, AMY | 2022 | Alive: Canada's Natural Health & Wellness Magazine |  |
| Sex and age group differences in the associations between sleep duration and BMI from adolescence to young adulthood. | Ames, Megan E.; Holfeld, Brett; Leadbeater, Bonnie J. | 2016 | Psychology & Health | 10.1080/08870446.2016.1163360 |
| SF-6D health state utilities for lifestyle, sociodemographic and clinical characteristics of a large international cohort of people with multiple sclerosis. | Campbell, Julie A.; Jelinek, George A.; Weiland, Tracey J.; Nag, Nupur; Neate, Sandra L.; Palmer, Andrew J.; Mulhern, Brendan; De Livera, Alysha; Simpson-Yap, Steve | 2020 | Quality of Life Research | 10.1007/s11136-020-02505-6 |
| Significant Improvement of Somatic Symptom Disorder With Brief Psychoeducational Intervention by PMHNP in Primary Care. | Johnson, Kim K.; Bennett, Carole; Rochani, Haresh | 2022 | Journal of the American Psychiatric Nurses Association | 10.1177/1078390320960524 |
| Skipping breakfast and its association with health risk behaviour and mental health among school-going adolescents in Curaçao: A cross-sectional national study. | Peltzer, Karl; Pengpid, Supa | 2020 | International Public Health Journal |  |
| Sleep quality of Shanghai residents: population-based cross-sectional study. | Wu, Wenjun; Jiang, Yonggen; Wang, Na; Zhu, Meiying; Liu, Xing; Jiang, Feng; Zhao, Genming; Zhao, Qi | 2020 | Quality of Life Research | 10.1007/s11136-019-02371-x |
| Sleep, stress, or social support?: Exploring the mechanisms that explain the relationship between student recreation center use and well-being. | Guan, Shu-Sha Angie; Xie, Hui; Boyns, David | 2020 | Journal of American College Health | 10.1080/07448481.2018.1535493 |
| Social Connection and Self-perceived Depression Among Adolescents: A Path Analytic Model for Abu Dhabi. | Badri, Masood; Khaili, Mugheer Al; Bahar, Muna Al; Yang, Guang; Reynhout, Georgina; Rashdi, Asma Al | 2021 | Journal of Child & Family Studies | 10.1007/s10826-020-01891-2 |
| Social inequalities in health: duration of unemployment unevenly effects on the health of men and women. | Acevedo, Paula; Mora-Urda, Ana I; Montero, Pilar | 2020 | European Journal of Public Health | 10.1093/eurpub/ckz180 |
| Sport participation moderates association between bullying and depressive symptoms in Italian adolescents. | Holbrook, Hannah M.; Voller, Fabio; Castellini, Giovanni; Silvestri, Caterina; Ricca, Valdo; Cassioli, Emanuele; Ivanova, Masha Y.; Hudziak, James J. | 2020 | Journal of Affective Disorders | 10.1016/j.jad.2020.03.142 |
| Sports and Physical Activity Attenuate the Emotional Toll of the Covid-19 Pandemic...Pediatric Research in Sports Medicine (PRiSM) 9th Annual Meeting, January 27-29, 2022, Houston, Texas | Walker, Gregory A.; Seehusen, Corrine N.; Armento, Aubrey; Provance, Aaron J.; Howell, David R. |  | Orthopaedic Journal of Sports Medicine | 10.1177/2325967121S00431 |
| SPORTS PERFORMANCE, SLEEP AND ANXIETY IN CHILDREN AND ADOLESCENTS: SECOND YEAR RESULTS OF A LONGITDUINAL STUDY...Pediatric Research in Sports Medicine Society (PRiSM) 7th Annual Meeting, January 23-25, 2020, Glendale, Arizona. | Sarkisova, Natalya; Herrera-Hamilton, Anita; Hartline, Kenneth D.; Perez, Iris; Wren, Tishya A. L.; Skaggs, David L.; Edison, Bianca | 2020 | Orthopaedic Journal of Sports Medicine | 10.1177/2325967120S00205 |
| Substance use among commercial motorcyclists and its relationship with life satisfaction and significant depressive symptoms. | Obadeji, Adetunji; Kumolalo, Banji F.; Ajiboye, Adedotun Samuel; Oluwole, Lateef Olutoyin; Oderinde, Kehinde O.; Ebeyi, Rose Chidindu | 2021 | Journal of Substance Use | 10.1080/14659891.2020.1800844 |
| Substance use behaviors in adolescent and young adult cancer patients: Associations with mental and physical health. | Asvat, Yasmin; King, Andrea C.; Smith, Lia J.; Lin, Xiaolei; Hedeker, Donald; Henderson, Tara O. | 2020 | Psycho-Oncology | 10.1002/pon.5378 |
| Survey of Clinical Practice Patterns of Korean Medicine Doctors for Anorexia in Children: A Preliminary Study for Clinical Practice Guidelines. | Lee, Jihong; Lee, Sun Haeng; Kim, Jae Hyun; Park, Yong Seok; Park, Sulgi; Chang, Gyu Tae | 2022 | Children | 10.3390/children9091409 |
| Swimming training improves mental health parameters, cognition and motor coordination in children with Attention Deficit Hyperactivity Disorder. | Silva, Luciano Acordi Da; Doyenart, Ramiro; Henrique Salvan, Paulo; Rodrigues, Welber; Felipe Lopes, João; Gomes, Karen; Thirupathi, Anand; Pinho, Ricardo Aurino De; Silveira, Paulo Cesar | 2020 | International Journal of Environmental Health Research | 10.1080/09603123.2019.1612041 |
| Systematic review of physical activity interventions assessing physical and mental health outcomes on patients with severe mental illness (SMI) within secure forensic settings. | Hassan, Jessica; Shannon, Stephen; Tully, Mark A.; McCartan, Claire; Davidson, Gavin; Bunn, Richard; Breslin, Gavin | 2022 | Journal of Psychiatric & Mental Health Nursing (John Wiley & Sons, Inc.) | 10.1111/jpm.12832 |
| Team Sport Athletes May Be Less Likely To Suffer Anxiety or Depression than Individual Sport Athletes. | Pluhar, Emily; McCracken, Caitlin; Griffith, Kelsey L.; Christino, Melissa A.; Sugimoto, Dai; Meehan III, William P. | 2019 | Journal of Sports Science & Medicine |  |
| Technology-enhanced visual desensitization home exercise program for post-concussive visually induced dizziness: a case series. | Hurtado, Julia E.; Heusel-Gillig, Lisa; Risk, Benjamin B.; Trofimova, Anna; Abidi, Syed A.; Allen, Jason W.; Gore, Russell K. | 2022 | Physiotherapy Theory & Practice | 10.1080/09593985.2020.1815259 |
| The association between physical activity, fitness and body mass index on mental well-being and quality of life in adolescents. | Eddolls, William T. B.; McNarry, Melitta A.; Lester, Leanne; Winn, Charles O. N.; Stratton, Gareth; Mackintosh, Kelly A. | 2018 | Quality of Life Research | 10.1007/s11136-018-1915-3 |
| The association of healthy lifestyle behaviors with mental health indicators among adolescents of different family affluence in Belgium. | Maenhout, L.; Peuters, C.; Cardon, G.; Compernolle, S.; Crombez, G.; DeSmet, A. | 2020 | BMC Public Health | 10.1186/s12889-020-09102-9 |
| The associations between lifestyle factors and mental well‐being in baccalaureate nursing students: An observational study. | Lee, Charlotte T.; Ting, Grace K.; Bellissimo, Nick; Khalesi, Saman | 2022 | Nursing & Health Sciences | 10.1111/nhs.12923 |
| The associations of healthful weight-control behaviors with psychological distress and changes in body mass index among young adults. | Elran-Barak, Roni | 2021 | Journal of Health Psychology | 10.1177/1359105319840693 |
| The Athlete Gratitude Group (TAGG): Effects of coach participation in a positive psychology intervention with youth athletes. | Gabana, Nicole T.; Wong, Y. Joel; D'Addario, Aaron; Chow, Graig M. | 2022 | Journal of Applied Sport Psychology | 10.1080/10413200.2020.1809551 |
| The Development, Pilot, and Process Evaluation of a Parent Mental Health Literacy Intervention Through Community Sports Clubs. | Hurley, Diarmuid; Allen, Mark S.; Swann, Christian; Okely, Anthony D.; Vella, Stewart A. | 2018 | Journal of Child & Family Studies | 10.1007/s10826-018-1071-y |
| The Dutch version of the self-report Child Activity and Limitations Interview in adolescents with chronic pain. | de Vries, Janneke E.; Dekker, Carolien; Bastiaenen, Carolien H. G.; Goossens, Mariëlle E. J. B.; Engelbert, Raoul H. H.; Verbunt, Jeanine A. M. C. F. | 2019 | Disability & Rehabilitation | 10.1080/09638288.2017.1407969 |
| The effect of COVID-19 quarantine on physical and social parameters of physical education providers and youth sport coaches. | Faraji, Sanaz; Ghayour Najafabadi, Mahboubeh; Rostad, Mitch; Anastasio, Albert Thomas | 2020 | Work | 10.3233/WOR-203329 |
| The effect of three exercise approaches on health-related quality of life, and factors associated with its improvement in chronic whiplash-associated disorders: analysis of a randomized controlled trial. | Landén Ludvigsson, Maria; Peterson, Gunnel; Peolsson, Anneli | 2019 | Quality of Life Research | 10.1007/s11136-018-2004-3 |
| The effectiveness of an indicated prevention programme for substance use in individuals with mild intellectual disabilities and borderline intellectual functioning: results of a quasi‐experimental study. | Schijven, Esmée P.; Hulsmans, Daan H. G.; VanDerNagel, Joanneke E. L.; Lammers, Jeroen; Otten, Roy; Poelen, Evelien A. P. | 2021 | Addiction | 10.1111/add.15156 |
| The effectiveness of positive mental health programs in adults: A systematic review. | Teixeira, Sónia Manuela Almeida; Coelho, Joana Catarina Ferreira; Sequeira, Carlos Alberto da Cruz; Lluch i Canut, Maria Teresa; Ferré-Grau, Carme | 2019 | Health & Social Care in the Community | 10.1111/hsc.12776 |
| The effectiveness of the COPE healthy lifestyles TEEN program: a school-based intervention in middle school adolescents with 12-month follow-up. | Ardic, Aysun; Erdogan, Semra | 2017 | Journal of Advanced Nursing (John Wiley & Sons, Inc.) | 10.1111/jan.13217 |
| The effects of breathing exercises in mothers of children with special health care needs:A randomized controlled trial. | Atilgan, E.D.; Tuncer, A. | 2021 | Journal of Back & Musculoskeletal Rehabilitation | 10.3233/BMR-200327 |
| The effects of pain neuroscience education and exercise on pain, muscle endurance, catastrophizing and anxiety in adolescents with chronic idiopathic neck pain: a school-based pilot, randomized and controlled study. | Andias, Rosa; Neto, Maritza; Silva, Anabela G. | 2018 | Physiotherapy Theory & Practice | 10.1080/09593985.2018.1423590 |
| The effects of physical exercise in children with attention deficit hyperactivity disorder: a systematic review and meta-analysis of randomized control trials. | Cerrillo‐Urbina, A. J.; García‐Hermoso, A.; Sánchez‐López, M.; Pardo‐Guijarro, M. J.; Santos Gómez, J. L.; Martínez‐Vizcaíno, V. | 2015 | Child: Care, Health & Development | 10.1111/cch.12255 |
| The Effects Of Resistance Exercise Training On Depressive Symptoms Among Young Adults: 1707...American College of Sports Medicine, Annual Meeting and World Congresses, May 31-June 4, 2022, San Diego, California | O'Sullivan, Darragh T.; Gordon, Brett R.; McDowell, Cillian P.; Lyons, Mark; Herring, Matthew P. | 2022 | Medicine & Science in Sports & Exercise | 10.1249/01.mss.0000880268.31506.e0 |
| The Impact of Auricular Vagus Nerve Stimulation on Pain and Life Quality in Patients with Fibromyalgia Syndrome. | Kutlu, Nazlı; Özden, Ali Veysel; Alptekin, Hasan Kerem; Alptekin, Jülide Öncü | 2020 | BioMed Research International | 10.1155/2020/8656218 |
| THE IMPACT OF COVID-19 RELATED SCHOOL CLOSURES AND SPORT CANCELLATIONS ON THE HEALTH OF ADOLESCENT ATHLETES...Pediatric Research in Sports Medicine (PRiSM), 8th Annual Meeting, 28-30 January, 2021. | McGuine, Timothy; Biese, Kevin; Hetzel, Scott; Kliethermes, Stephanie; Reardon, Claudia; Bell, David; Brooks, M. Alison; Watson, Andrew | 2021 | Orthopaedic Journal of Sports Medicine | 10.1177/2325967121S00170 |
| The Impact of Equine Therapy and an Audio-Visual Approach Emphasizing Rhythm and Beat Perception in Children with Developmental Coordination Disorder. | Hession, Caren E.; Law Smith, Miriam J.; Watterson, David; Oxley, Nigel; Murphy, Barbara A. | 2019 | Journal of Alternative & Complementary Medicine | 10.1089/acm.2017.0242 |
| The Impact of Partner Performance on Emotions in Doubles Racquet Sports. | Deck, Sarah; Hall, Craig; Wilson, Philip M. | 2021 | Research Quarterly for Exercise & Sport | 10.1080/02701367.2020.1726270 |
| The Impact of sport particiaption on the Health of Adolescents During the CoVID-19 pandemic...Pediatric Research in Sports Medicine (PRiSM) 9th Annual Meeting, January 27-29, 2022, Houston, Texas. | McGuine, Timothy; Biese, Kevin; Schwarz, Alison; Dickman, James; Hetzel, Scott; Reardon, Claudia; Watson, Andrew |  | Orthopaedic Journal of Sports Medicine | 10.1177/2325967121S00426 |
| The longitudinal effect of leisure time physical activity on reduced depressive symptoms: The ARIRANG Study. | Jo, Hoon; Lee, Jinhee; Lee, Solam; Lee, Hunju; Ahn, Yeon-Soon; Koh, Sang-Baek | 2021 | Journal of Affective Disorders | 10.1016/j.jad.2021.01.014 |
| The Mediating Role of Self-Efficacy in the Association Between Diabetes Education and Support and Self-Care Management. | Juarez, Lucía D.; Presley, Caroline A.; Howell, Carrie R.; Agne, April A.; Cherrington, Andrea L. | 2022 | Health Education & Behavior | 10.1177/10901981211008819 |
| The Nature of Physical Activity Programming in Irish Primary Schools for Children with Autism Spectrum Disorder. | Carey, Marie; Kinsella, Sharon; Healy, Sean; Knott, Fiona; Sheehan, Damien; O'Malley, Niall | 2022 | Palaestra |  |
| The Need for Novel Treatments for Bipolar Depression. | Hardy, Lyons | 2022 | Psychiatric Times |  |
| The positive association between perceived parental responsiveness and self-esteem, anxiety, and thriving among youth rugby players: A multigroup analysis. | Rouquette, Olivier Y.; Knight, Camilla J.; Lovett, Victoria E.; Barrell, Donald; Heuzé, Jean-Philippe | 2021 | Journal of Sports Sciences | 10.1080/02640414.2021.1883311 |
| The Profile and Prognosis of Youth With Status Migrainosus: Results From an Observational Study. | Orr, Serena L.; Turner, Abigail; Kabbouche, Marielle A.; Horn, Paul S.; O'Brien, Hope L.; Kacperski, Joanne; LeCates, Susan; White, Shannon; Weberding, Jessica; Miller, Mimi N.; Powers, Scott W.; Hershey, Andrew D. | 2020 | Headache: The Journal of Head & Face Pain | 10.1111/head.13767 |
| THE RELATIONSHIP BETWEEN MENTAL HEALTH HISTORY AND SYMPTOMS, SEX, AND RECOVERY TIME IN A CONCUSSED PEDIATRIC POPULATION...Pediatric Research in Sports Medicine Society (PRiSM) 7th Annual Meeting, January 23-25, 2020, Glendale, Arizona. | Allred, Richard Cameron; Stremlau, Sara; Gerkin, Richard; Erickson, Steven; Pardini, Jamie | 2020 | Orthopaedic Journal of Sports Medicine | 10.1177/2325967120S00233 |
| The relationship between physical activity and mental health among adolescents in six middle-income countries: A cross-sectional study. | Arat, Gizem; Wong, Paul Wai-Ching | 2017 | Child & Youth Services | 10.1080/0145935X.2017.1297202 |
| The relationship between physical activity, mental wellbeing and symptoms of mental health disorder in adolescents: a cohort study. | Bell, Sarah Louise; Audrey, Suzanne; Gunnell, David; Cooper, Ashley; Campbell, Rona | 2019 | International Journal of Behavioral Nutrition & Physical Activity | 10.1186/s12966-019-0901-7 |
| The relationship between screen-based sedentary behaviors and symptoms of depression and anxiety in youth: a systematic review of moderating variables. | Zink, Jennifer; Belcher, Britni R.; Imm, Kellie; Leventhal, Adam M. | 2020 | BMC Public Health | 10.1186/s12889-020-08572-1 |
| The Research On Raising Great Kids. | Flint, Daniel | 2021 | Psychology Today |  |
| The Role of Reported Affective Symptoms and Anxiety in Recovery Trajectories After Sport-Related Concussion. | D'Alonzo, Bernadette A.; Bretzin, Abigail C.; Wiebe, Douglas J.; Fiore, Russell; VanPatten, Bryn; Levine, William N.; Desai, Natasha; Wentzel, David C.; Sucheski-Drake, Amy; Karlson, Kristine A.; Wang, Frank; Richardson, Lars; Port, Nicholas L.; Saffarian, Mathew; Vesci, Brian; Gay, Michael; Day, Carly; Putukian, Margot; Esopenko, Carrie; Wheeler, Matthew B. | 2022 | American Journal of Sports Medicine | 10.1177/03635465221098112 |
| The Smart Use of Smartphones in Pediatrics. | Solecki, Susan | 2020 | Journal of Pediatric Nursing | 10.1016/j.pedn.2020.06.001 |
| "Tomorrow, I'll Be Fine": Impacts and coping mechanisms in adolescents and young adults with primary dysmenorrhoea. | Allyn, Katherine; Evans, Subhadra; Seidman, Laura C.; Payne, Laura A. | 2020 | Journal of Advanced Nursing (John Wiley & Sons, Inc.) | 10.1111/jan.14460 |
| Trauma informed public health nursing visits to parents and children. | Ballard, Julianne; George, Liz; Zazueta‐Lara, Eva; Turner, Laura; Aguado, Jesús; Law, Jennifer; Alger, Renée | 2019 | Public Health Nursing | 10.1111/phn.12634 |
| Unspoken Playground Rules Discourage Adolescent Physical Activity in School: A Focus Group Study of Constructs in the Prototype Willingness Model. | Wheatley, Catherine M.; Davies, Emma L.; Dawes, Helen | 2018 | Qualitative Health Research | 10.1177/1049732317744534 |
| Using Critical Incident Technique to Investigate Anxiety in Physical Activity Settings. | Dasinger, Timothy M.; Solmon, Melinda A. | 2021 | Journal of Sport & Exercise Psychology | 10.1123/jsep.2020-0065 |
| Volume and social context of physical activity in association with mental health, anxiety and depression among youth. | Doré, Isabelle; O'Loughlin, Jennifer L.; Beauchamp, Guy; Martineau, Marc; Fournier, Louise; O'Loughlin, Jennifer L | 2016 | Preventive Medicine | 10.1016/j.ypmed.2016.09.006 |
| Within-person variability in curiosity during daily life and associations with well-being. | Lydon‐Staley, David M.; Zurn, Perry; Bassett, Danielle S.; Lydon-Staley, David M | 2020 | Journal of Personality | 10.1111/jopy.12515 |
| Youth and Caregiver Physical Activity and Sedentary Time: HCHS/SOL Youth. | Gallo, Linda C.; Roesch, Scott P.; McCurley, Jessica L.; Isasi, Carmen R.; Sotres-Alvarez, Daniela; Delamater, Alan M.; Van Horn, Linda; Arredondo, Elva M.; Perreira, Krista M.; Buelna, Christina; Qibin Qi; Vidot, Denise C.; Carnethon, Mercedes R. | 2017 | American Journal of Health Behavior | 10.5993/AJHB.41.1.7 |
| ‘I’m managing myself’: how and why people use St John’s wort as a strategy to manage their mental health risk. | Lewis, Sophie; Willis, Karen; Kokanovic, Renata; Pirotta, Marie | 2015 | Health, Risk & Society | 10.1080/13698575.2015.1096328 |
| 134. The Effect of the COVID-19 Pandemic on the Video Gaming Behavior, Depressive Symptoms, Sleep Quality, and Physical Activity of Excessive Video Gamers...Society for Adolescent Health and Medicine (SAHM) Annual Meeting (Virtual)-Caring for Adolescents | Akbulut, Ozlem; Pehlivanturk-Kizilkan, Melis; Akgül, Sinem; Derman, Orhan; Kanbur, Nuray | 2022 | Journal of Adolescent Health | 10.1016/j.jadohealth.2022.01.051 |
| 647 The Superhero in You: Engaging Pediatric Patients in Therapeutic Exercises. | Yelvington, Miranda L | 2021 | Journal of Burn Care & Research | 10.1093/jbcr/irab032.297 |
| 8 Simple Ways to Soothe An Anxious Child: Telling kids to "calm down" or "buck up" doesn't work. Here's what to do instead. | Leyba, Erin | 2020 | Psychology Today |  |
| 8 WAYS TO HELP A CHILD ACHIEVE CALM: Telling anxious kids to calm down won't work. Here's what could. | LEYBA, ERIN | 2021 | Psychology Today |  |
| A Case Study on Promoting Neuroplasticity in a Patient With Schizophrenia. | Puskar, Kathryn; Slivka, Cynthia; Lee, Heeyoung; Martin, Colin; Witt, Matthew | 2016 | Perspectives in Psychiatric Care | 10.1111/ppc.12104 |
| A comparison of the effects of hatha yoga and resistance exercise on mental health and well-being in sedentary adults: A pilot study. | Taspinar, Betul; Aslan, Ummuhan Bas; Agbuga, Bulent; Taspinar, Ferruh | 2014 | Complementary Therapies in Medicine | 10.1016/j.ctim.2014.03.007 |
| A dyadic multiple mediation model of patient and spouse stressors predicting patient dietary and exercise adherence via depression symptoms and diabetes self-efficacy. | Anderson, Jared; Novak, Joshua; Johnson, Matthew; Deitz, Sharon; Walker, Ann; Wilcox, Allison; Lewis, Virginia; Robbins, David | 2016 | Journal of Behavioral Medicine | 10.1007/s10865-016-9796-9 |
| A Multimodal Mindfulness Training to Address Mental Health Symptoms in Providers Who Care for and Interact With Children in Relation to End-of-Life Care. | Thomas, O'Neil; Patricia; Kathryn, Szakatis; Tom, Fitzgerald | 2017 | American Journal of Hospice & Palliative Medicine | 10.1177/1049909116660688 |
| A Multimodal Protocol to Diminish Pain Following Common Orthopedic Sports Procedures: Can We Eliminate Postoperative Opioids? | Moutzouros, Vasilios; Jildeh, Toufic R.; Khalil, Lafi S.; Schwartz, Kaylin; Hasan, Laith; Matar, Robert N.; Okoroha, Kelechi R. | 2020 | Arthroscopy: The Journal of Arthroscopy & Related Surgery | 10.1016/j.arthro.2020.04.018 |
| A novel approach to the creation of a labelling lexicon for improving emotion analysis in text. | Segura Navarrete, Alejandra; Martinez-Araneda, Claudia; Vidal-Castro, Christian; Rubio-Manzano, Clemente | 2021 | Electronic Library | 10.1108/EL-04-2020-0110 |
| A partially supervised physical activity program for adult and adolescent survivors of childhood cancer (SURfit): study design of a randomized controlled trial [NCT02730767]. | Rueegg, Corina S.; Kriemler, Susi; Zuercher, Simeon J.; Schindera, Christina; Renner, Andrea; Hebestreit, Helge; Meier, Christian; Eser, Prisca; von der Weid, Nicolas X. | 2017 | BMC Cancer | 10.1186/s12885-017-3801-8 |
| A randomised trial on walking exercise and banana consumption on self-reported depression symptoms among female adolescents in Surakarta, Indonesia. | Putra, Egy Sunanda; Wasita, Brian; Anantanyu, Sapja | 2018 | Malaysian Journal of Nutrition |  |
| A Randomized Control Intervention Investigating the Effects of Acute Exercise on Emotional Regulation. | Edwards, Meghan K.; Rhodes, Ryan E.; Loprinzi, Paul D. | 2017 | American Journal of Health Behavior | 10.5993/AJHB.41.5.2 |
| A study of coach‐team perceptual distance concerning the coach‐created motivational climate in youth sport. | Gjesdal, Siv; Stenling, Andreas; Solstad, Bård E.; Ommundsen, Yngvar | 2019 | Scandinavian Journal of Medicine & Science in Sports | 10.1111/sms.13306 |
| A study of self-perception and communication success as perceived by adolescents with cochlear implants and their significant others. | Bantwal, Anuradha R.; Deshpande, Rashmi; Indurkar, Rewa; Bhatnagar, Salaj; Wadhera, Meenakshi; Sridhara, Aditya; Lalwani, Neera; Agarwal, Asha; Oza, Ramesh K.; Narayan, Neevita; Sasidharan, P.; Mallikarjun, B.; Saha, Chandan; Bhale, Priya; Deshpande, Shweta; Mandke, Kalyani | 2021 | Cochlear Implants International: An Interdisciplinary Journal | 10.1080/14670100.2021.1875577 |
| A systematic review of physical therapy interventions for patients with anorexia and bulemia nervosa. | Vancampfort, Davy; Vanderlinden, Johan; De Hert, Marc; Soundy, Andrew; Adámkova, Milena; Skjaerven, Liv Helvik; Catalán-Matamoros, Daniel; Lundvik Gyllensten, Amanda; Gómez-Conesa, Antonia; Probst, Michel | 2014 | Disability & Rehabilitation | 10.3109/09638288.2013.808271 |
| Acute Exercise Effects among Young Adults with Analogue Generalized Anxiety Disorder. | HERRING, MATTHEW P.; MONROE, DEREK C.; GORDON, BRETT R.; HALLGREN, MATS; CAMPBELL, MARK J. | 2019 | Medicine & Science in Sports & Exercise | 10.1249/MSS.0000000000001860 |
| Addressing engagement, anger and aggression through the Rock Water Program: Rural adolescent males' perceptions. | Edwards, Paul; Mortel, Thea; Stevens, John | 2017 | Australian Journal of Rural Health | 10.1111/ajr.12332 |
| Adherence to Multiple Treatment Recommendations in Adolescents and Young Adults with Cancer: A Mixed Methods, Multi-Informant Investigation. | Psihogios, Alexandra M.; Schwartz, Lisa A.; Ewing, Kylie B.; Czerniecki, Bryn; Kersun, Leslie S.; Pai, Ahna L.H.; Deatrick, Janet A.; Barakat, Lamia P. | 2020 | Journal of Adolescent & Young Adult Oncology | 10.1089/jayao.2020.0013 |
| Adolescent Burmese Refugees Perspectives on Determinants of Health. | Dixit, Avika; Miner, Emily M.; Wiehe, Sarah E.; McHenry, Megan S. | 2018 | Journal of Immigrant & Minority Health | 10.1007/s10903-017-0648-7 |
| Adolescent Health Risk Behaviors: Parental Concern and Concordance Between Parent and Adolescent Reports. | Gersh, Elon; Richardson, Laura P.; Katzman, Katherine; Spielvogle, Heather; Arghira, Adriana Cristina; Chuan Zhou; Mccarty, Carolyn A. | 2018 | Academic Pediatrics | 10.1016/j.acap.2017.08.012 |
| Adolescent obesity and risk of mental disorder. | Harikha, Igha Vinda; Kalalo, Royke Tony | 2021 | International Journal of Child & Adolescent Health |  |
| Adolescents and young women with functional hypothalamic amenorrhoea: is it time to move beyond the hormonal profile? | Tranoulis, Anastasios; Soldatou, Alexandra; Georgiou, Dimitra; Mavrogianni, Despoina; Loutradis, Dimitios; Michala, Lina | 2020 | Archives of Gynecology & Obstetrics | 10.1007/s00404-020-05499-1 |
| Adolescents and youth in adult hospitals: psychosocial assessment on admission - an evaluation of the youth care plan. | Sturrock, Tegan; Steinbeck, Kate | 2013 | Australian Journal of Advanced Nursing |  |
| Adolescents' experiences of menarche and menstruation in disadvantaged schools in South Africa: a qualitative exploration. | Fennie, Thelma; Moletsane, Mokgadi; Padmanabhanunni, Anita | 2021 | Health Education (0965-4283) | 10.1108/HE-12-2020-0122 |
| Adolescents with unexplained chest pain reported depression and impaired emotional and social functioning. | Eliacik, Kayi; Bolat, Nurullah; Kanik, Ali; Malas, Nasuh; Demircan, Tülay; Hortu, Hacer; Özyurt, Gonca; Orbatu, Dilek; Alaygut, Demet; Guven, Baris | 2020 | Acta Paediatrica | 10.1111/apa.15144 |
| Aerobic exercise or stretching as add-on to inpatient treatment of depression: Similar antidepressant effects on depressive symptoms and larger effects on working memory for aerobic exercise alone. | Imboden, Christian; Gerber, Markus; Beck, Johannes; Holsboer-Trachsler, Edith; Pühse, Uwe; Hatzinger, Martin | 2020 | Journal of Affective Disorders | 10.1016/j.jad.2020.07.052 |
| Age-related differences in physical activity and depressive symptoms among 10−19-year-old adolescents: A population based study. | Baldursdottir, Birna; Valdimarsdottir, Heiddis B.; Krettek, Alexandra; Gylfason, Haukur Freyr; Sigfusdottir, Inga Dora | 2017 | Psychology of Sport & Exercise | 10.1016/j.psychsport.2016.10.007 |
| An analysis of Time 4U, a therapeutic group for women with postnatal depression. | Hall, Kathy; Grundy, Sheila | 2014 | Community Practitioner |  |
| An Exploratory Study to Assess the Level of Internet Addiction and its Impact on the Health of Adolescents in Selected Schools of District Mohali, Punjab. | Sharma, Deeksha; Nitakumari; Verma, Geeta | 2019 | International Journal of Nursing Education | 10.5958/0974-9357.2019.00031.X |
| An Integrative Review of Yoga and Mindfulness-Based Approaches for Children and Adolescents with Asthma. | Lack, Sharon; Brown, Roy; Kinser, Patricia A. | 2020 | Journal of Pediatric Nursing | 10.1016/j.pedn.2020.03.006 |
| Anxiety And Depressive Symptoms Differ Between Levels Of Physical Activity And Screen Time Among Adolescents...2021 ACSM Annual Meeting & World Congresses [Virtual], June 1 -5, 2021 | Forte, Chloe; McDowell, Cillian P.; McDonnacha, Ciaran; Herring, Matthew P. | 2021 | Medicine & Science in Sports & Exercise | 10.1249/01.mss.0000762736.40087.09 |
| Anxiety and spirituality in university students: a cross-sectional study. | de Cássia Lopes Chaves, Erika; Hollanda Iunes, Denise; de Castro Moura, Caroline; César Carvalho, Leonardo; Maria Silva, Andréia; Campos de Carvalho, Emília | 2015 | Revista Brasileira de Enfermagem | 10.1590/0034-7167.2015680318i |
| Anxiety as a Moderator of the Relationship Between Body Image and Restrained Eating. | Doumit, Rita; Zeeni, Nadine; Sanchez Ruiz, Maria Jose; Khazen, Georges | 2016 | Perspectives in Psychiatric Care | 10.1111/ppc.12126 |
| Anxiety on Quiet Eye and Performance of Youth Pistol Shooters. | Shah, Eesha J.; Chow, Jia Yi; Lee, Marcus J.C. | 2020 | Journal of Sport & Exercise Psychology | 10.1123/jsep.2019-0174 |
| Assessment of Overweight and Obesity Status in Patients with Depression Referred to Baharloo Hospital in Tehran: Possible Roles For Vitamin D? | Kaviani, Mina; Nikooyeh, Bahareh; Zand, Hamid; Yaghmaei, Parichehreh; Neyestani, Tirang R. | 2021 | Nutrition & Food Sciences Research |  |
| Assisted Cycling Therapy (ACT) Improves Adaptive Behaviors in Adolescents with Down Syndrome. | Ringenbach, S. D. R.; Holzapfel, S. D.; Arnold, N. E.; Nam, K.; Lopez, C.; Chen, C.-C.; Buman, M. P.; Youngstedt, S. D.; Teslevich, J.; Wallace, K. C. | 2020 | Journal of Developmental & Physical Disabilities | 10.1007/s10882-019-09706-z |
| Association Between Bullying Victimization and Health Risk Behaviors Among High School Students in the United States. | Hertz, Marci Feldman; Everett Jones, Sherry; Barrios, Lisa; David‐Ferdon, Corinne; Holt, Melissa | 2015 | Journal of School Health | 10.1111/josh.12339 |
| Association Between Concussion History and Factors Relating to Cognitive, Behavioral, and Emotional Health Among American High School Athletes: A Cross-sectional Analysis. | Knell, Gregory; Burkhart, Scott O.; Caze, Todd J.; Polousky, John D.; Kohl III, Harold W.; Messiah, Sarah E. | 2020 | American Journal of Sports Medicine | 10.1177/0363546520938776 |
| Association between dental fear, physical activity and physical and mental well-being among Finnish university students. | Pohjola, Vesa; Puolakka, Antti; Kunttu, Kristina; Virtanen, Jorma I. | 2020 | Acta Odontologica Scandinavica | 10.1080/00016357.2019.1649457 |
| Association between muscle strength and depressive symptoms among Chinese female college freshmen: a cross-sectional study. | Ren, Zhongyu; Cao, Jianhua; Li, Yingke; Cheng, Peng; Cao, Bing; Hao, Zongji; Yao, Hui; Shi, Dongzhe; Liu, Bin; Chen, Chang; Yang, Guang; Peng, Li; Guo, Liya | 2020 | BMC Musculoskeletal Disorders | 10.1186/s12891-020-03478-w |
| Association between Non-Competitive Physical Exercise and Menstrual Disorders. | Peinado-Molina, Rocio Adriana; Peinado-Molina, Maria Dolores; Molina-Ibañez, Maria Dolores; Martínez-Galiano, Juan Miguel | 2020 | African Journal of Reproductive Health | 10.29063/ajrh2020/v24i1.8 |
| Association between premorbid neuropsychological conditions and pediatric mild traumatic brain injury/concussion recovery time and symptom severity: a systematic review. | Goreth, Michelle Borzik; Palokas, Michelle | 2019 | JBI Database of Systematic Reviews & Implementation Reports | 10.11124/JBISRIR-2017-004008 |
| Association of Adolescent Sport Participation With Cognition and Depressive Symptoms in Early Adulthood. | Bohr, Adam D.; Boardman, Jason D.; McQueen, Matthew B. | 2019 | Orthopaedic Journal of Sports Medicine | 10.1177/2325967119868658 |
| Association of depression symptoms with receipt of healthcare provider advice on physical activity among US adults. | Grabovac, I; Stefanac, S; Smith, L; Haider, S; Cao, C; Jackson, S E; Dorner, T E; Waldhoer, T; Rieder, A; Yang, L | 2020 | Journal of Affective Disorders | 10.1016/j.jad.2019.11.023 |
| Association of Exercise Intolerance With Emotional Distress, Attainment of Social Roles, and Health-Related Quality of Life Among Adult Survivors of Childhood Cancer. | Hayek, Samah; Brinkman, Tara M.; Plana, Juan C.; Joshi, Vijaya M.; Leupker, Russell V.; Durand, Jean B.; Green, Daniel M.; Partin, Robyn E.; Santucci, Aimee K.; Howell, Rebecca M.; Srivastava, Deo Kumar; Hudson, Melissa M.; Robison, Leslie L.; Armstrong, Gregory T.; Ness, Kirsten K. | 2020 | JAMA Oncology | 10.1001/jamaoncol.2020.2054 |
| Association of Physical Activity and Screen Time with Psychiatric Distress in Children and Adolescents: CASPIAN-IV Study. | Taheri, Ehsaneh; Heshmat, Ramin; Motlagh, Mohammad Esmaeil; Ardalan, Gelayol; Asayesh, Hamid; Qorbani, Mostafa; Kelishadi, Roya; Esmaeil Motlagh, Mohammad | 2019 | Journal of Tropical Pediatrics | 10.1093/tropej/fmy063 |
| Association of Screen Time and Depression in Adolescence. | Boers, Elroy; Afzali, Mohammad H.; Newton, Nicola; Conrod, Patricia | 2019 | JAMA Pediatrics | 10.1001/jamapediatrics.2019.1759 |
| Associations between aerobic and muscle-strengthening exercise with depressive symptom severity among 17,839 U.S. adults. | Bennie, Jason A.; Teychenne, Megan J.; De Cocker, Katrien; Biddle, Stuart J.H. | 2019 | Preventive Medicine | 10.1016/j.ypmed.2019.02.022 |
| Associations between depressive symptoms, cigarette smoking, and cardiovascular health: Longitudinal results from CARDIA. | Carroll, Allison J.; Huffman, Mark D.; Zhao, Lihui; Jacobs, David R.; Stewart, Jesse C.; Kiefe, Catarina I.; Brunner, Wendy; Liu, Kiang; Hitsman, Brian | 2020 | Journal of Affective Disorders | 10.1016/j.jad.2019.09.049 |
| Attention-deficit/hyperactivity disorder casts a long shadow: findings from a population-based study of adult women with self-reported ADHD. | Fuller‐Thomson, E.; Lewis, D. A.; Agbeyaka, S. K. | 2016 | Child: Care, Health & Development | 10.1111/cch.12380 |
| Barriers, Benefits, and Strategies for Physical Activity in Patients With Schizophrenia. | Rastad, Cecilia; Martin, Cathrin; Åsenlöf, Pernilla | 2014 | Physical Therapy | 10.2522/ptj.20120443 |
| Baseline Demographic, Anthropometric, Psychosocial, and Behavioral Characteristics of Rural, Southern Women in Early Pregnancy. | Thomson, Jessica; Tussing-Humphreys, Lisa; Goodman, Melissa; Olender, Sarah | 2016 | Maternal & Child Health Journal | 10.1007/s10995-016-2016-y |
| Behavioral and Psychosocial Characteristics Among Head Start Childcare Providers. | Ling, Jiying | 2018 | Journal of School Nursing | 10.1177/1059840517725791 |
| Benefits of Physical Activity during Pregnancy and Postpartum: An Umbrella Review. | DIPIETRO, LORETTA; EVENSON, KELLY R.; BLOODGOOD, BONNY; SPROW, KYLE; TROIANO, RICHARD P.; PIERCY, KATRINA L.; VAUX-BJERKE, ALISON; POWELL, KENNETH E. | 2019 | Medicine & Science in Sports & Exercise | 10.1249/MSS.0000000000001941 |
| Beyond the thin ideal: Development and validation of the Fit Ideal Internalization Test (FIIT) for women. | Uhlmann, Laura R.; Donovan, Caroline L.; Zimmer-Gembeck, Melanie J. | 2020 | Psychological Assessment | 10.1037/pas0000773 |
| Biographical disruption, adjustment and reconstruction of everyday occupations and work participation after mild traumatic brain injury. A focus group study. | Sveen, Unni; Søberg, Helene Lundgaard; Østensjø, Sigrid | 2016 | Disability & Rehabilitation | 10.3109/09638288.2015.1129445 |
| Brain health supplements, decoded: New research to examine and consider. |  | 2020 | Alive: Canada's Natural Health & Wellness Magazine |  |
| Brief report: Associations of physical activity with anxiety and depression symptoms and status among adolescents. | McDowell, Cillian P.; MacDonncha, Ciaran; Herring, Matthew P. | 2017 | Journal of Adolescence | 10.1016/j.adolescence.2016.12.004 |
| Brief report: Longitudinal associations between physical activity, sleep disturbance and depressive symptoms in adolescent girls. | Raudsepp, Lennart; Vink, Kristi | 2019 | Journal of Adolescence | 10.1016/j.adolescence.2019.02.003 |
| Building an Evidence-Based Mental Health Program for Children With History of Early Adversity. | Kroupina, Maria; Moberg, Stephanie; Vermeulen, Marlous | 2015 | Zero to Three |  |
| Bullying victimization as a predictor of suicidal ideation and suicide attempt among senior high school students in Ghana: Results from the 2012 Ghana Global School-Based Health Survey. | Baiden, Philip; Shrestha, Nibedita; Tonui, Betty C.; Kuuire, Vincent Z.; Dako-Gyeke, Mavis; Peters, Kersley K. | 2019 | Journal of School Violence | 10.1080/15388220.2018.1486200 |
| Can Couple Level Attachment Mediate the Influence of Depressive Symptoms on Health? | Sandberg, Jonathan G.; Novak, Joshua R.; Bates, Tasha | 2016 | American Journal of Family Therapy | 10.1080/01926187.2016.1145082 |
| Can dental pain be a cause of irritability in children and family distress? | Gomes, Monalisa Cesarino; Perazzo, Matheus França; Neves, Érick Tássio Barbosa; Souza, Emilly Gabrielle Carlos; Araújo, Luíza Jordânia Serafim; Martins, Carolina Castro; Paiva, Saul Martins; Granville-Garcia, Ana Flávia | 2020 | Journal of Public Health (09431853) | 10.1007/s10389-019-01025-z |
| Cardiovascular fitness in late adolescent males and later risk of serious non-affective mental disorders: a prospective, population-based study. | Nyberg, J.; Kuhn, H. G.; Åberg, M. A. I.; Henriksson, M.; Rosengren, A.; Söderberg, M.; Åberg, N. D.; Waern, M. | 2018 | Psychological Medicine | 10.1017/S0033291717001763 |
| Celiac Disease Symptoms in Athletes: Prevalence Indicators of Perceived Quality of Life. | Leone, James E.; Wise, Kimberly A.; Mullin, Elizabeth M.; Gray, Kimberly A.; Szlosek, Philip A.; Griffin, Matthew F.; Jordan, Cara A. | 2020 | Sports Health: A Multidisciplinary Approach | 10.1177/1941738120905137 |
| Change in basic need frustration in relation to perfectionism, anxiety, and performance in elite junior performers. | Haraldsen, Heidi M.; Solstad, Bård Erlend; Ivarsson, Andreas; Halvari, Hallgeir; Abrahamsen, Frank E. | 2020 | Scandinavian Journal of Medicine & Science in Sports | 10.1111/sms.13614 |
| Changes in the Prevalence and Correlates of Weight-Control Behaviors and Weight Perception in Adolescents in the UK, 1986-2015. | Solmi, Francesca; Sharpe, PhD, Helen; Gage, Suzanne H.; Maddock, Jane; Lewis, Glyn; Patalay, Praveetha | 2021 | JAMA Pediatrics | 10.1001/jamapediatrics.2020.4746 |
| Characteristics and Behaviors of Older Male Anabolic Steroid Users. | Ip, Eric J.; Trinh, Karen; Tenerowicz, Michael J.; Pal, Jai; Lindfelt, Tristan A.; Perry, Paul J. | 2015 | Journal of Pharmacy Practice | 10.1177/0897190014527319 |
| Characteristics Associated with Successful Weight Management in Youth with Obesity. | Gorecki, Michelle C.; Feinglass, Joseph M.; Binns, Helen J. | 2019 | Journal of Pediatrics | 10.1016/j.jpeds.2019.05.039 |
| Childhood overweight and obesity and the risk of depression across the lifespan. | Gibson-Smith, Deborah; Halldorsson, Thorhallur I.; Bot, Mariska; Brouwer, Ingeborg A.; Visser, Marjolein; Thorsdottir, Inga; Birgisdottir, Bryndis E.; Gudnason, Vilmundur; Eiriksdottir, Gudny; Launer, Lenore J.; Harris, Tamara B.; Gunnarsdottir, Ingibjorg | 2020 | BMC Pediatrics | 10.1186/s12887-020-1930-8 |
| Children's Physical Activity and Depression: A Meta-analysis. | Korczak, Daphne J.; Madigan, Sheri; Colasanto, Marlena | 2017 | Pediatrics | 10.1542/peds.2016-2266 |
| Child's Number of Activities as a Moderator of Depressive Symptoms. | Ryan, Paige; Kaskas, Maysa; Davis, Thompson; NoackLeSage, Franziska; Lilly, Megan; Castagna, Peter; Shaheen, Georgia | 2017 | Journal of Child & Family Studies | 10.1007/s10826-017-0853-y |
| Cigarette- and snus-modified association between unprotected exposure to noise from hunting rifle caliber weapons and high frequency hearing loss. A cross-sectional study among swedish hunters. | Honeth, Louise; StrÃ¶m, Peter; Ploner, Alexander; Bagger-SjÃ¶bÃ¤ck, Dan; Rosenhall, Ulf; NyrÃ©n, Olof | 2016 | Noise & Health | 10.4103/1463-1741.195796 |
| CLINICAL PREDICTORS OF SYMPTOM RESOLUTION FOR CHILDREN AND ADOLESCENTS WITH SPORT-RELATED CONCUSSION...PRiSM 6th Annual Meeting, January 17-19, 2019, Atlanta, Georgia. | Howell, David R.; Potter, Morgan N.; Kirkwood, Michael W.; Wilson, Pamela E.; Provance, Aaron; Wilson, Julie C. |  | Orthopaedic Journal of Sports Medicine | 10.1177/2325967119S00007 |
| Clinical Reaction-Time Performance Factors in Healthy Collegiate Athletes. | Caccese, Jaclyn B.; Eckner, James T.; Franco-MacKendrick, Lea; Hazzard, Joseph B.; Meng Ni; Broglio, Steven P.; McAllister, Thomas W.; McCrea, Michael; Buckley, Thomas A. | 2020 | Journal of Athletic Training (Allen Press) | 10.4085/1062-6050-164-19 |
| Clustering of lifestyle factors and the relationship with depressive symptoms among adolescents in Northeastern China. | Cao, Ruilin; Gao, Tingting; Hu, Yueyang; Qin, Zeying; Ren, Hui; Liang, Leilei; Li, Chuanen; Mei, Songli | 2020 | Journal of Affective Disorders | 10.1016/j.jad.2020.05.064 |
| Combating the Dangers of Sedentary Activity on Child and Adolescent Mental Health During the Time of COVID-19. | Mittal, Vijay A.; Firth, Joseph; Kimhy, David | 2020 | Journal of the American Academy of Child & Adolescent Psychiatry | 10.1016/j.jaac.2020.08.003 |
| Community-Based Preventive Interventions for Depression and Anxiety in Women. | Maleki, Fatemeh Mollarahimi; Massahikhaleghi, Parissa; Tehrani-Banihashemi, Arash; Davoudi, Farnoush; Nojomi, Marzieh | 2020 | Archives of Iranian Medicine (AIM) |  |
| Comorbid Depression and Obesity: Correlates and Synergistic Association With Noncommunicable Diseases Among Australian Men. | Tilahun Nigatu Haregu; Tayu Lee, John; Oldenburg, Brian; Armstrong, Gregory; Haregu, Tilahun Nigatu; Lee, John Tayu | 2020 | Preventing Chronic Disease | 10.5888/pcd17.190420 |
| Comparison of the effect of educational software and booklet on anxiety and pain during labour: a randomised controlled clinical trial. | Abbasi, Parastoo; Mohammad-Alizadeh Charandabi, Sakineh; Mirghafourvand, Mojgan | 2021 | Journal of Obstetrics & Gynaecology | 10.1080/01443615.2020.1736017 |
| Conceptions of Adolescent Friendship Quality in Sport and Music Domains. | Phillips Reichter, Alison; Weiss, Maureen R. | 2019 | Research Quarterly for Exercise & Sport | 10.1080/02701367.2019.1632412 |
| Consistent participation in organized physical activity predicts emotional adjustment in children. | BriÃ¨re, FrÃ©dÃ©ric N.; Imbeault, Arianne; Goldfield, Gary S.; Pagani, Linda S. | 2020 | Pediatric Research | 10.1038/s41390-019-0417-5 |
| Correction to: Is There an Association Between Diet, Physical Activity and Depressive Symptoms in the Perinatal Period? An Analysis of the UPBEAT Cohort of Obese Pregnant Women...Wilson CA, Seed P, Flynn AC, et al. Is There an Association Between Diet, Ph | Wilson, Claire A.; Seed, Paul; Flynn, Angela C.; Howard, Louise M.; Molyneaux, Emma; Sigurdardottir, Julie; Poston, Lucilla | 2020 | Maternal & Child Health Journal | 10.1007/s10995-020-02957-9 |
| Correlates of short sleep duration among adolescents. | Widome, Rachel; Berger, Aaron T.; Lenk, Kathleen M.; Erickson, Darin J.; Laska, Melissa N.; Iber, Conrad; Kilian, Gudrun; Wahlstrom, Kyla | 2019 | Journal of Adolescence | 10.1016/j.adolescence.2019.10.011 |
| Correlation of PROMIS Physical Function, Pain Interference, and Depression in Pediatric and Adolescent Patients in the Ambulatory Sports Medicine Clinic. | Makhni, Eric C.; Meldau, Jason E.; Blanchett, Jacob; Borowsky, Peter; Stephens, Jeffrey; Muh, Stephanie; Moutzouros, Vasilios | 2019 | Orthopaedic Journal of Sports Medicine | 10.1177/2325967119851100 |
| Could greater physical activity reduce population prevalence and socio-economic inequalities in children's mental health problems? A policy simulation. | Sungano Chigogora; Pearce, Anna; Law, Catherine; Viner, Russell; Chittleborough, Catherine; Griffiths, Lucy J.; Hope, Steven; Chigogora, Sungano | 2020 | Epidemiology | 10.1097/EDE.0000000000001113 |
| Could Postnatal Womenâ€™s Groups Be Used to Improve Outcomes for Mothers and Children in High-Income Countries? A Systematic Review. | Sikorski, Catherine; Van Hees, Sietske; Lakhanpaul, Monica; Benton, Lorna; Martin, Jennifer; Costello, Anthony; Heys, Michelle | 2018 | Maternal & Child Health Journal | 10.1007/s10995-018-2606-y |
| COVID-19, Mental health and intervention-priorities among Northern Italian University students...14th European Public Health Conference (Virtual), Public health futures in a changing world, November 10-12, 2021. | Patrono, A.; Placidi, D.; Calza, S.; Brunelli, P.; Cagna, G.; Manco, A.; Renzetti, S.; Rota, M.; Moncada, S.; Lucchini, R. | 2021 | European Journal of Public Health |  |
| Cross-sectional and prospective relationships of passive and mentally active sedentary behaviours and physical activity with depression. | Hallgren, Mats; Nguyen, Thi-Thuy-Dung; Owen, Neville; Stubbs, Brendon; Vancampfort, Davy; Lundin, Andreas; Dunstan, David; Bellocco, Rino; Lagerros, Ylva Trolle | 2020 | British Journal of Psychiatry | 10.1192/bjp.2019.60 |
| Cross-sectional association of exercise, strengthening activities, and cardiorespiratory fitness on generalized anxiety, panic and depressive symptoms. | Loprinzi, Paul D.; Addoh, Ovuokerie; Wong Sarver, Nina; Espinoza, Ingrid; Mann, Joshua R. | 2017 | Postgraduate Medicine | 10.1080/00325481.2017.1336054 |
| Cross-sectional associations between long-term exposure to particulate matter and depression in China: The mediating effects of sunlight, physical activity, and neighborly reciprocity. | Wang, Ruoyu; Liu, Ye; Xue, Desheng; Yao, Yao; Liu, Penghua; Helbich, Marco | 2019 | Journal of Affective Disorders | 10.1016/j.jad.2019.02.007 |
| Cumulative Lifetime Marijuana Use and Incident Cardiovascular Disease in Middle Age: The Coronary Artery Risk Development in Young Adults (CARDIA) Study. | Reis, Jared P.; Auer, Reto; Bancks, Michael P.; Jr, David C. Goff; Lewis, Cora E.; Pletcher, Mark J.; Rana, Jamal S.; Shikany, James M.; Sidney, Stephen | 2017 | American Journal of Public Health | 10.2105/AJPH.2017.303654 |
| Cyberbulling as a new form of a threat: a physiological, psychological and medicinal aspects. | Mikhaylovsky, Mikhail N.; Lopatkova, Irina V.; Komarova, Nataliya M.; Rueva, Evgeniya O.; Tereschuk, Konstantin S.; Emelyanenkova, Anna V. | 2019 | Electronic Journal of General Medicine | 10.29333/ejgm/114268 |
| Depression and Its Comorbid Conditions More Serious in Women than in Men in the United States. | Kim, Woo Kyoung; Shin, Dayeon; Song, Won O. | 2015 | Journal of Women's Health (15409996) | 10.1089/jwh.2014.4919 |
| Depression, Quality of Life, Physical Activity, and the Impact of Drugs on Sexual Activity in a Population-Based Sample, Ages 20-59Â Years. | Steinke, Elaine E.; Mosack, Victoria; Hill, Twyla J. | 2018 | Issues in Mental Health Nursing | 10.1080/01612840.2017.1413463 |
| Depressive symptom trajectories and physical health: Persistence of problems from adolescence to young adulthood. | Ames, Megan E.; Leadbeater, Bonnie J. | 2018 | Journal of Affective Disorders | 10.1016/j.jad.2018.07.001 |
| Depressive Symptoms, Body Mass Index, and Physical Activity Self-Efficacy in African American Children. | Williams, Y'Esha V.; Cowan, Patricia A.; Graff, Joyce C. | 2020 | Journal of Child & Family Studies | 10.1007/s10826-020-01761-x |
| Designing and Validating a Questionnaire on Healthy Lifestyle to Reduce Depressive Symptoms among Adolescents. | Tajik, Esra; Abd Latiff, Latiffah; Chin Yit Siew; Awang, Hamidin; Adznam, Siti Nur'Asyura | 2020 | Iranian Journal of Psychiatry |  |
| Determinants of life satisfaction in Iranian children and adolescents: the CASPIANâ€IV study. | Kelishadi, Roya; Mirmoghtadaee, Parisa; Qorbani, Mostafa; Heshmat, Ramin; Motlagh, Mohammad Esmaeil; Magoul, Arman; Raeesi, Sina; Mansourian, Morteza; Gorabi, Armita Mahdavi; Safiri, Saeid | 2018 | Child & Adolescent Mental Health | 10.1111/camh.12239 |
| Developing webâ€based health guidance for coaches and parents in child athletics (track and field). | Jacobsson, Jenny; Ekberg, Joakim; Timpka, Toomas; Haggren RÃ¥sberg, Lena; SjÃ¶berg, Marina; Mirkovic, Dejan; Nilsson, Sverker | 2020 | Scandinavian Journal of Medicine & Science in Sports | 10.1111/sms.13661 |
| Diet and physical activity in the association between depression and metabolic syndrome: Constances study. | Matta, Joane; Hoertel, Nicolas; Kesse-Guyot, Emmanuelle; Plesz, Marie; Wiernik, Emmanuel; Carette, Claire; Czernichow, SÃ©bastien; Limosin, FrÃ©dÃ©ric; Goldberg, Marcel; Zins, Marie; Lemogne, CÃ©dric | 2019 | Journal of Affective Disorders | 10.1016/j.jad.2018.09.072 |
| Differential treatment effects of an integrated motivational interviewing and exercise intervention on depressive symptom profiles and associated factors: A randomised controlled cross-over trial among youth with major depression. | Nasstasia, Yasmina; Baker, Amanda L.; Lewin, Terry J.; Halpin, Sean A.; Hides, Leanne; Kelly, Brian J.; Callister, Robin | 2019 | Journal of Affective Disorders | 10.1016/j.jad.2019.08.035 |
| Digital Phenotyping With Mobile and Wearable Devices: Advanced Symptom Measurement in Child and Adolescent Depression. | Sequeira, Lydia; Battaglia, Marco; Perrotta, Steve; Merikangas, Kathleen; Strauss, John | 2019 | Journal of the American Academy of Child & Adolescent Psychiatry | 10.1016/j.jaac.2019.04.011 |
| Distinct health behavior and psychosocial profiles of young adult survivors of childhood cancers: a mixed methods study. | Lowe, Kincaid; Escoffery, Cam; Mertens, Ann; Berg, Carla; Mertens, Ann C; Berg, Carla J | 2016 | Journal of Cancer Survivorship | 10.1007/s11764-015-0508-1 |
| Do asthmatics benefit from music therapy? A systematic review. | Sliwka, Agnieszka; Wloch, Tomasz; Tynor, Dariusz; Nowobilski, Roman | 2014 | Complementary Therapies in Medicine | 10.1016/j.ctim.2014.07.002 |
| Do Injured Adolescent Athletes and Their Parents Agree on the Athletes' Level of Psychologic and Physical Functioning? | Oosterhoff, Jacobien H. F.; Bexkens, Rens; Vranceanu, Ana-Maria; Oh, Luke S. | 2018 | Clinical Orthopaedics & Related ResearchÂ® | 10.1007/s11999.0000000000000071 |
| Does exercise habit strength moderate the relationship between emotional distress and short-term memory in Malaysian primary school children? | Zainol, Nurul Ain; Hashim, Hairul Anuar | 2015 | Psychology, Health & Medicine | 10.1080/13548506.2014.955034 |
| Does manual therapy provide additional benefit to breathing retraining in the management of dysfunctional breathing? A randomised controlled trial. | Jones, Mandy; Troup, Fiona; Nugus, John; Roughton, Michael; Hodson, Margaret; Rayner, Charlotte; Bowen, Frances; Pryor, Jennifer | 2015 | Disability & Rehabilitation | 10.3109/09638288.2014.941020 |
| Does Physical Activity Moderate The Influence Of Sedentary Behavior On Health In Young People?...2021 ACSM Annual Meeting & World Congresses [Virtual], June 1 -5, 2021 | Schembri, Emanuel; Heinz, Andreas; Samuel, Robin | 2021 | Medicine & Science in Sports & Exercise | 10.1249/01.mss.0000761192.16245.9b |
| Domain-specific physical activity and sedentary behavior during pregnancy and postpartum depression risk in the French EDEN and ELFE cohorts. | van der Waerden, Judith; Nakamura, Aurelie; Pryor, Laura; Charles, Marie-Aline; El-Khoury, Fabienne; Dargent-Molina, Patricia | 2019 | Preventive Medicine | 10.1016/j.ypmed.2019.02.012 |
| Dropout from exercise randomized controlled trials among people with anxiety and stress-related disorders: A meta-analysis and meta-regression. | Vancampfort, Davy; SÃ¡nchez, Carlos Pelayo Ramos; Hallgren, Mats; Schuch, Felipe; Firth, Joseph; Rosenbaum, Simon; Van Damme, Tine; Stubbs, Brendon | 2021 | Journal of Affective Disorders | 10.1016/j.jad.2021.01.003 |
| Dyadic Effects of Individual and Friend on Physical Activity in College Students. | Kim, Gwang Suk; Lee, Chung Yul; Kim, In Sook; Lee, Tae Hwa; Cho, Eunhee; Lee, Hyeonkyeong; McCreary, Linda L.; Kim, Su Hee | 2015 | Public Health Nursing | 10.1111/phn.12176 |
| Eating Together and Health-Related Quality of Life Among Korean Adults. | Choi, Min-Jung; Park, Yong Gyu; Kim, Yang Hyun; Cho, Kyung Hwan; Nam, Ga Eun | 2020 | Journal of Nutrition Education & Behavior | 10.1016/j.jneb.2019.11.013 |
| Economic Hardship and Depression Among Women in Latino Farmworker Families. | Pulgar, Camila; Trejo, Grisel; Suerken, Cynthia; Ip, Edward; Arcury, Thomas; Quandt, Sara | 2016 | Journal of Immigrant & Minority Health | 10.1007/s10903-015-0229-6 |
| Effect of a relaxation training exercise on behaviour, anxiety, and pain during buccal infiltration anaesthesia in children: Randomized clinical trial. | Sridhar, Sowmya; Suprabha, Baranya Shrikrishna; Shenoy, Ramya; Shwetha, Kunnumal Thekkeveetil; Rao, Arathi | 2019 | International Journal of Paediatric Dentistry | 10.1111/ipd.12497 |
| Effect of Alternate Nostril Breathing Exercise on Experimentally Induced Anxiety in Healthy Volunteers Using the Simulated Public Speaking Model: A Randomized Controlled Pilot Study. | Kamath, Ashwin; Urval, Rathnakar P.; Shenoy, Ashok K. | 2017 | BioMed Research International | 10.1155/2017/2450670 |
| Effect of Diagnosed Sleep Disorders on Baseline Concussion Symptom, Cognitive, and Balance Assessments in Collegiate Athletes. | McAllister-Deitrick, Jamie; Trbovich, Alicia M.; Broglio, Steven P.; McCrea, Michael; McAllister, Thomas W.; Kontos, Anthony P. | 2020 | American Journal of Sports Medicine | 10.1177/0363546520902701 |
| Effect of Meridian Acupressure on Aerobic Performance of Healthy Young Population: A Randomized Controlled Study. | Ahmedov, Shahin; Filiz, Baris | 2018 | Journal of Alternative & Complementary Medicine | 10.1089/acm.2017.0089 |
| Effect of Social Anxiety on the Adoption of Robotic Training Partner. | Zhu, Dong Hong; Deng, Zhong Zhun | 2021 | CyberPsychology, Behavior & Social Networking | 10.1089/cyber.2020.0179 |
| Effect of tele-rehabilitation on glucose control, exercise capacity, physical fitness, muscle strength and psychosocial status in patients with type 2 diabetes: A double blind randomized controlled trial. | Duruturk, Neslihan; Ã–zkÃ¶slÃ¼, Manolya Acar | 2019 | Primary Care Diabetes | 10.1016/j.pcd.2019.03.007 |
| Effectiveness of Acceptance and Commitment Therapy in Increasing Resilience and Reducing Attrition of Injured US Navy Recruits. | Udell, Christopher J; Ruddy, Julie L; Procento, Philip M | 2018 | Military Medicine | 10.1093/milmed/usx109 |
| Effectiveness of an Exercise-Based Active Rehabilitation Intervention for Youth Who Are Slow to Recover After Concussion. | Gauvin-Lepage, JÃ©rÃ´me; Friedman, Debbie; Grilli, Lisa; Sufrategui, Maria; De Matteo, Carol; Iverson, Grant L.; Gagnon, Isabelle | 2020 | Clinical Journal of Sport Medicine | 10.1097/JSM.0000000000000634 |
| Effectiveness of Chinese Martial Arts and Philosophy to Reduce Reactive and Proactive Aggression in Schoolchildren. | Lai Chu Fung, Annis; Ka Hung Lee, Toney; Fung, Annis Lai Chu; Lee, Toney Ka Hung | 2018 | Journal of Developmental & Behavioral Pediatrics | 10.1097/DBP.0000000000000565 |
| Effectiveness of School-based Health Center Delivery of a Cognitive Skills Building Intervention in Young, Rural Adolescents: Potential Applications for Addiction and Mood. | Carr, Kayla L.; Stewart, Mary W. | 2019 | Journal of Pediatric Nursing | 10.1016/j.pedn.2019.04.013 |
| Effectiveness of Universal Self-regulationâ€“Based Interventions in Children and Adolescents: A Systematic Review and Meta-analysis. | Pandey, Anuja; Hale, Daniel; Das, Shikta; Goddings, Anne-Lise; Blakemore, Sarah-Jayne; Viner, Russell M. | 2018 | JAMA Pediatrics | 10.1001/jamapediatrics.2018.0232 |
| Effects of a self-managed home-based walking intervention on psychosocial health outcomes for breast cancer patients receiving chemotherapy: a randomised controlled trial. | Gokal, Kajal; Wallis, Deborah; Ahmed, Samreen; Boiangiu, Ion; Kancherla, Kiran; Munir, Fehmidah | 2016 | Supportive Care in Cancer | 10.1007/s00520-015-2884-5 |
| Effects of Acute Stretching on Cognitive Function and Mood States of Physically Inactive Young Adults. | Sudo, Mizuki; Ando, Soichi | 2020 | Perceptual & Motor Skills | 10.1177/0031512519888304 |
| Effects of Yoga on Heart Rate Variability and Depressive Symptoms in Women: A Randomized Controlled Trial. | Chu, I-Hua; Wu, Wen-Lan; Lin, I-Mei; Chang, Yu-Kai; Lin, Yuh-Jen; Yang, Pin-Chen | 2017 | Journal of Alternative & Complementary Medicine | 10.1089/acm.2016.0135 |
| Efficacy of Homeopathy in Addition to a Multidisciplinary Intervention for Overweight or Obesity in Mexican Adolescents: Study Protocol for a Randomized, Double-Blind, Placebo-Controlled Trial. | MacÃ­as-CortÃ©s, Emma; Arellano-Ãlvarez, SaraÃ­; Vega-Monroy, Sandra; Vera-PÃ©rez, Violeta; Llanes-GonzÃ¡lez, Lidia; SÃ¡nchez-Navarrete, Pilar; Enciso-GonzÃ¡lez, Dolores | 2020 | Homeopathy | 10.1055/s-0039-1697927 |
| ENERGY BALANCE AND VITAL SIGNS IN PEDIATRIC PATIENTS...Pediatric Research in Sports Medicine Society (PRiSM) 7th Annual Meeting, January 23-25, 2020, Glendale, Arizona. | Young, Julie A.; Napolitano, Jessica; Rauh, Mitchell J.; Nichols, Jeanne; Fischer, Anastasia N. | 2020 | Orthopaedic Journal of Sports Medicine | 10.1177/2325967120S00206 |
| Evaluating Multiple Domains of Health in High School Athletes With Sport-Related Concussion. | Williams, Richelle M.; Johnson, Rachel S.; Snyder Valier, Alison R.; Curtis Bay, R.; Valovich McLeod, Tamara C. | 2021 | Journal of Sport Rehabilitation | 10.1123/jsr.2019-0517 |
| Evaluating the Impact of an Anti-stigma Intervention on Pharmacy Students' Willingness to Counsel People Living with Mental Illness. | Bamgbade, Benita; Barner, Jamie; Ford, Kentya | 2017 | Community Mental Health Journal | 10.1007/s10597-016-0075-6 |
| Evaluation of an online cognitive behavioural therapy weight loss programme as an adjunct to anti-obesity medications and lifestyle interventions. | Tham, Marlene; Chong, Terence WH | 2020 | Australasian Psychiatry | 10.1177/1039856219871882 |
| Evaluation of Partner Violence in Female Patients with Fibromyalgia Syndrome...Congress of Clinical Child and Adult Psychiatry held in Istanbul on 23-25 September 2016. | Onur, Ozge Sahmelikoglu; Cesur, Ender; Donmezler, Fadime Gizem; Aydin, Filiz Yildiz; Vural, Meltem; Guru, Meltem | 2020 | Medical Journal of Bakirkoy | 10.5222/BMJ.2020.58076 |
| Evaluation of the DSM-5 Severity Specifier for Bulimia Nervosa in Treatment-Seeking Youth. | Dakanalis, Antonios; Colmegna, Fabrizia; Zanetti, Maria Assunta; Di Giacomo, Ester; Riva, Giuseppe; Clerici, Massimo | 2018 | Child Psychiatry & Human Development | 10.1007/s10578-017-0735-y |
| Evaluation of the effectiveness of relaxation in lowering the level of anxiety in young adults - a pilot study. | WILCZYÅƒSKA, DOMINIKA; ÅYSAK-RADOMSKA, ANNA; PODCZARSKA-GÅOWACKA, MAGDALENA; ZAJT, JOLANTA; DORNOWSKI, MARCIN; SKONIECZNY, PAWEÅ; WilczyÅ„ska, Dominika; Podczarska-GÅ‚owacka, Magdalena; Skonieczny, PaweÅ‚ | 2019 | International Journal of Occupational Medicine & Environmental Health | 10.13075/ijomeh.1896.01457 |
| "Even If They're Being Bad, Maybe They Need a Chance to Run Around": What Children Think About Recess. | Fink, Dale B.; Ramstetter, Catherine L. | 2018 | Journal of School Health | 10.1111/josh.12704 |
| Evidence that Swimming May Be Protective of Knee Osteoarthritis: Data from the Osteoarthritis Initiative. | Lo, Grace H.; Ikpeama, Uzoh E.; Driban, Jeffrey B.; Kriska, Andrea M.; McAlindon, Timothy E.; Petersen, Nancy J.; Storti, Kristi L.; Eaton, Charles B.; Hochberg, Marc C.; Jackson, Rebecca D.; Kwoh, C. Kent; Nevitt, Michael C.; Suarezâ€Almazor, Maria E.; Suarez-Almazor, Maria E | 2020 | PM & R: Journal of Injury, Function & Rehabilitation | 10.1002/pmrj.12267 |
| Examination of a board game approach to children's involvement in family-based weight management vs. traditional family-based behavioral counseling in primary care. | Sen, Merve; Uzuner, Arzu; Akman, Mehmet; Bahadir, Aliye Tugba; Borekci, Nazire Oncul; Viggiano, Emanuela | 2018 | European Journal of Pediatrics | 10.1007/s00431-018-3177-z |
| Examination of life quality, mental conditions and cognitive status of people over the age of 90: Results of a Hungarian local research. | Czibere, Ibolya; RÃ¡cz, Andrea; SzilvÃ¡si, Henrietta; Szikszai, Zita; Imre, SÃ¡ndor | 2019 | Central European Journal of Public Health | 10.21101/cejph.a4753 |
| Examining a training effect on the state anxiety response to an acute bout of exercise in low and high anxious individuals. | Lucibello, KM; Parker, J; Heisz, JJ; Lucibello, K M; Heisz, J J | 2019 | Journal of Affective Disorders | 10.1016/j.jad.2018.12.063 |
| Examining the Effects of Exercise on Pattern Separation and the Moderating Effects of Mood Symptoms. | Bernstein, Emily E.; McNally, Richard J. | 2019 | Behavior Therapy | 10.1016/j.beth.2018.09.007 |
| Excessive exercise among adolescents with eating disorders: examination of psychological and demographic variables. | Renz, Jessica A.; Fisher, Martin; Vidair, Hilary B.; Hirsch, Dina; Malizio, Joan; Barger, Hamutal; Fornari, Victor | 2019 | International Journal of Adolescent Medicine & Health | 10.1515/ijamh-2017-0032 |
| Exercise and Diet as Potential Moderators Between Trauma, Posttraumatic Stress, Depression, and Relationship Quality Among Emerging Adults. | Smith-Marek, Erika N.; Durtschi, Jared; Brown, Cameron; Dharnidharka, Prerana | 2016 | American Journal of Family Therapy | 10.1080/01926187.2016.1145080 |
| Exercise and mental health literacy in an Australian adult population. | Stanton, Robert; Rebar, Amanda; Rosenbaum, Simon | 2019 | Depression & Anxiety (1091-4269) | 10.1002/da.22851 |
| Exercise as an adjunct treatment for postpartum depression for women living in an inner cityâ€”A pilot study. | Forsyth, Jacky; Boath, Elizabeth; Henshaw, Carol; Brown, Hannah | 2017 | Health Care for Women International | 10.1080/07399332.2017.1295049 |
| Exercise as Medicine for Mental and Substance Use Disorders: A Meta-review of the Benefits for Neuropsychiatric and Cognitive Outcomes. | Ashdown-Franks, Garcia; Firth, Joseph; Carney, Rebekah; Carvalho, Andre F.; Hallgren, Mats; Koyanagi, Ai; Rosenbaum, Simon; Schuch, Felipe B.; Smith, Lee; Solmi, Marco; Vancampfort, Davy; Stubbs, Brendon | 2020 | Sports Medicine | 10.1007/s40279-019-01187-6 |
| EXERCISE DURING PREGNANCY: A Prescription for Improved Maternal/Fetal Well-being. | Davenport, Margie H. | 2020 | ACSM's Health & Fitness Journal | 10.1249/FIT.0000000000000602 |
| Exercise for Depression: A Feasibility Trial Exploring Neural Mechanisms. | Gujral, Swathi; Aizenstein, Howard; Reynolds III, Charles F.; Butters, Meryl A.; Grove, George; Karp, Jordan F.; Erickson, Kirk I.; Reynolds, Charles F 3rd | 2019 | American Journal of Geriatric Psychiatry | 10.1016/j.jagp.2019.01.012 |
| Exercise for PTSD in Women Veterans: A Proof-of-Concept Study. | Shivakumar, Geetha; Anderson, Elizabeth H.; SurÃ­s, Alina M.; North, Carol S. | 2017 | Military Medicine | 10.7205/MILMED-D-16-00440 |
| Experimentally investigating the joint effects of physical activity and sedentary behavior on depression and anxiety: A randomized controlled trial. | Blough, Jeremiah; Loprinzi, Paul D. | 2018 | Journal of Affective Disorders | 10.1016/j.jad.2018.07.019 |
| Exploring patients' attitudes to different intervention approaches for supporting psychosocial needs. | Taylor, Francesca; Hare, Jennifer; Combes, Gill | 2016 | Journal of Renal Care | 10.1111/jorc.12182 |
| Exploring the differentiated relationship between appearance and fitnessâ€related social anxiety and the risk of eating disorders and depression in young adults. | Alcarazâ€IbÃ¡Ã±ez, Manuel; Sicilia, Ãlvaro; Paterna, Adrian | 2019 | Scandinavian Journal of Psychology | 10.1111/sjop.12584 |
| Facilitators and Barriers of Sleep in Young Adults With Type 1 Diabetes. | Griggs, Stephanie; Whittemore, Robin; Redeker, Nancy S.; Grey, Margaret | 2020 | Diabetes Educator | 10.1177/0145721720916179 |
| Factors Associated with Migraine in the General Population of Spain: Results from the European Health Survey 2014. | Roy, RubÃ©n; SÃ¡nchez-RodrÃ­guez, Elisabet; GalÃ¡n, Santiago; Racine, MÃ©lanie; Castarlenas, Elena; Jensen, Mark P; MirÃ³, Jordi | 2019 | Pain Medicine | 10.1093/pm/pny093 |
| Factors Associated with Suicidal Thought and Help-Seeking Behaviour in Transition-Aged Youth versus Adults. | MacKinnon, Nathalie; Colman, Ian | 2016 | Canadian Journal of Psychiatry | 10.1177/0706743716667417 |
| Facts and ideas from anywhere. | Roberts, William C. | 2019 | Baylor University Medical Center Proceedings | 10.1080/08998280.2019.1583692 |
| Fatigue in young people with Duchenne muscular dystrophy. | Elâ€Aloul, Basmah; Speechley, Kathy N; Wei, Yi; Wilk, Piotr; Campbell, Craig; El-Aloul, Basmah | 2020 | Developmental Medicine & Child Neurology | 10.1111/dmcn.14248 |
| Fatigue-Related Cognitive-Behavioral Factors in Survivors of Childhood Cancer: Comparison with Chronic Fatigue Syndrome and Survivors of Adult-Onset Cancer. | van Deuren, Sylvia; van Dulmen-den Broeder, Eline; Boonstra, Amilie; Gielissen, Marieke; Blijlevens, Nicole; Loonen, Jacqueline; Knoop, Hans | 2021 | Journal of Adolescent & Young Adult Oncology | 10.1089/jayao.2020.0094 |
| Feasibility, acceptability, and effectiveness of a multidisciplinary intervention in childhood obesity from primary care: Nutrition, physical activity, emotional regulation, and family. | SepÃºlveda, Ana R.; Solano, Santos; Blanco, Miriam; Lacruz, Tatiana; Veiga, Oscar | 2020 | European Eating Disorders Review | 10.1002/erv.2702 |
| Females and males do not differ for fatigability, muscle damage and magnitude of the repeated bout effect following maximal eccentric contractions. | Bruce, Christina D.; Ruggiero, Luca; Dix, Gabriel U.; Cotton, Paul D.; McNeil, Chris J. | 2021 | Applied Physiology, Nutrition & Metabolism | 10.1139/apnm-2020-0516 |
| Frequency of Breakfast and Physical Fitness among Chinese College Students. | Yufei Cui; Wenbo Zhang; Qiang Gong; Yanbo Chen; Shulei Chen; Ziqiang Wu | 2018 | American Journal of Health Behavior | 10.5993/AJHB.42.1.15 |
| Functional Resistance Training and Affective Response in Female College-Age Students. | FARO, JAMIE; WRIGHT, JULIE A.; HAYMAN, LAURA L.; HASTIE, MARISA; GONA, PHILIMON N.; WHITELEY, JESSICA A. | 2019 | Medicine & Science in Sports & Exercise | 10.1249/MSS.0000000000001895 |
| Gender differences in irritable bowel syndrome among medical students at Inner Mongolia Medical University, China: a cross-sectional study. | Wang, Ying; Jin, Feng; Chi, Baofeng; Duan, Shengyun; Zhang, Qing; Liu, Ying; Hao, Wenli; Sun, Juan | 2016 | Psychology, Health & Medicine | 10.1080/13548506.2016.1144890 |
| GENDER PREFERENCES OF YOUTH ATHLETES FOR THEIR SPORTS MEDICINE PROVIDERS: A SYSTEMATIC REVIEW...Pediatric Research in Sports Medicine (PRiSM), 8th Annual Meeting, 28-30 January, 2021. | Chung, Jane S.; Merkel, Donna; Carter, Cordelia W.; Kraus, Emily; Rizzone, Katherine | 2021 | Orthopaedic Journal of Sports Medicine | 10.1177/2325967121S00108 |
| Gender-specific prevalence and associated factors of major depressive disorder and generalized anxiety disorder in a Chinese rural population: the Henan rural cohort study. | Luo, Zhicheng; Li, Yuqian; Hou, Yitan; Liu, Xiaotian; Jiang, Jingjing; Wang, Yan; Liu, Xue; Qiao, Dou; Dong, Xiaokang; Li, Ruiying; Wang, Fang; Wang, Chongjian | 2019 | BMC Public Health | 10.1186/s12889-019-8086-1 |
| German recommendations for physical activity and physical activity promotion in adults with noncommunicable diseases. | Geidl, Wolfgang; Abu-Omar, Karim; Weege, Mayra; Messing, Sven; Pfeifer, Klaus | 2020 | International Journal of Behavioral Nutrition & Physical Activity | 10.1186/s12966-020-0919-x |
| Globus Sensation And Retching In A 21-year-old Male Football Player.: 1322...American College of Sports Medicine, Annual Meeting and World Congresses, May 31-June 4, 2022, San Diego, California | Donohoe, Brian; Goldman, Joshua; Polster, Douglas | 2022 | Medicine & Science in Sports & Exercise | 10.1249/01.mss.0000879160.87669.31 |
| Characteristics Associated with Successful Weight Management in Youth with Obesity. | Gorecki, Michelle C.; Feinglass, Joseph M.; Binns, Helen J. | 2019 | Journal of Pediatrics | 10.1016/j.jpeds.2019.05.039 |
| Childhood overweight and obesity and the risk of depression across the lifespan. | Gibson-Smith, Deborah; Halldorsson, Thorhallur I.; Bot, Mariska; Brouwer, Ingeborg A.; Visser, Marjolein; Thorsdottir, Inga; Birgisdottir, Bryndis E.; Gudnason, Vilmundur; Eiriksdottir, Gudny; Launer, Lenore J.; Harris, Tamara B.; Gunnarsdottir, Ingibjorg | 2020 | BMC Pediatrics | 10.1186/s12887-020-1930-8 |
| Children's Physical Activity and Depression: A Meta-analysis. | Korczak, Daphne J.; Madigan, Sheri; Colasanto, Marlena | 2017 | Pediatrics | 10.1542/peds.2016-2266 |
| Child's Number of Activities as a Moderator of Depressive Symptoms. | Ryan, Paige; Kaskas, Maysa; Davis, Thompson; NoackLeSage, Franziska; Lilly, Megan; Castagna, Peter; Shaheen, Georgia | 2017 | Journal of Child & Family Studies | 10.1007/s10826-017-0853-y |
| Cigarette- and snus-modified association between unprotected exposure to noise from hunting rifle caliber weapons and high frequency hearing loss. A cross-sectional study among swedish hunters. | Honeth, Louise; StrÃ¶m, Peter; Ploner, Alexander; Bagger-SjÃ¶bÃ¤ck, Dan; Rosenhall, Ulf; NyrÃ©n, Olof | 2016 | Noise & Health | 10.4103/1463-1741.195796 |
| CLINICAL PREDICTORS OF SYMPTOM RESOLUTION FOR CHILDREN AND ADOLESCENTS WITH SPORT-RELATED CONCUSSION...PRiSM 6th Annual Meeting, January 17-19, 2019, Atlanta, Georgia. | Howell, David R.; Potter, Morgan N.; Kirkwood, Michael W.; Wilson, Pamela E.; Provance, Aaron; Wilson, Julie C. |  | Orthopaedic Journal of Sports Medicine | 10.1177/2325967119S00007 |
| Clinical Reaction-Time Performance Factors in Healthy Collegiate Athletes. | Caccese, Jaclyn B.; Eckner, James T.; Franco-MacKendrick, Lea; Hazzard, Joseph B.; Meng Ni; Broglio, Steven P.; McAllister, Thomas W.; McCrea, Michael; Buckley, Thomas A. | 2020 | Journal of Athletic Training (Allen Press) | 10.4085/1062-6050-164-19 |
| Clustering of lifestyle factors and the relationship with depressive symptoms among adolescents in Northeastern China. | Cao, Ruilin; Gao, Tingting; Hu, Yueyang; Qin, Zeying; Ren, Hui; Liang, Leilei; Li, Chuanen; Mei, Songli | 2020 | Journal of Affective Disorders | 10.1016/j.jad.2020.05.064 |
| Combating the Dangers of Sedentary Activity on Child and Adolescent Mental Health During the Time of COVID-19. | Mittal, Vijay A.; Firth, Joseph; Kimhy, David | 2020 | Journal of the American Academy of Child & Adolescent Psychiatry | 10.1016/j.jaac.2020.08.003 |
| Community-Based Preventive Interventions for Depression and Anxiety in Women. | Maleki, Fatemeh Mollarahimi; Massahikhaleghi, Parissa; Tehrani-Banihashemi, Arash; Davoudi, Farnoush; Nojomi, Marzieh | 2020 | Archives of Iranian Medicine (AIM) |  |
| Comorbid Depression and Obesity: Correlates and Synergistic Association With Noncommunicable Diseases Among Australian Men. | Tilahun Nigatu Haregu; Tayu Lee, John; Oldenburg, Brian; Armstrong, Gregory; Haregu, Tilahun Nigatu; Lee, John Tayu | 2020 | Preventing Chronic Disease | 10.5888/pcd17.190420 |
| Comparison of the effect of educational software and booklet on anxiety and pain during labour: a randomised controlled clinical trial. | Abbasi, Parastoo; Mohammad-Alizadeh Charandabi, Sakineh; Mirghafourvand, Mojgan | 2021 | Journal of Obstetrics & Gynaecology | 10.1080/01443615.2020.1736017 |
| Conceptions of Adolescent Friendship Quality in Sport and Music Domains. | Phillips Reichter, Alison; Weiss, Maureen R. | 2019 | Research Quarterly for Exercise & Sport | 10.1080/02701367.2019.1632412 |
| Consistent participation in organized physical activity predicts emotional adjustment in children. | BriÃ¨re, FrÃ©dÃ©ric N.; Imbeault, Arianne; Goldfield, Gary S.; Pagani, Linda S. | 2020 | Pediatric Research | 10.1038/s41390-019-0417-5 |
| Correction to: Is There an Association Between Diet, Physical Activity and Depressive Symptoms in the Perinatal Period? An Analysis of the UPBEAT Cohort of Obese Pregnant Women...Wilson CA, Seed P, Flynn AC, et al. Is There an Association Between Diet, Ph | Wilson, Claire A.; Seed, Paul; Flynn, Angela C.; Howard, Louise M.; Molyneaux, Emma; Sigurdardottir, Julie; Poston, Lucilla | 2020 | Maternal & Child Health Journal | 10.1007/s10995-020-02957-9 |
| Correlates of short sleep duration among adolescents. | Widome, Rachel; Berger, Aaron T.; Lenk, Kathleen M.; Erickson, Darin J.; Laska, Melissa N.; Iber, Conrad; Kilian, Gudrun; Wahlstrom, Kyla | 2019 | Journal of Adolescence | 10.1016/j.adolescence.2019.10.011 |
| Correlation of PROMIS Physical Function, Pain Interference, and Depression in Pediatric and Adolescent Patients in the Ambulatory Sports Medicine Clinic. | Makhni, Eric C.; Meldau, Jason E.; Blanchett, Jacob; Borowsky, Peter; Stephens, Jeffrey; Muh, Stephanie; Moutzouros, Vasilios | 2019 | Orthopaedic Journal of Sports Medicine | 10.1177/2325967119851100 |
| Could greater physical activity reduce population prevalence and socio-economic inequalities in children's mental health problems? A policy simulation. | Sungano Chigogora; Pearce, Anna; Law, Catherine; Viner, Russell; Chittleborough, Catherine; Griffiths, Lucy J.; Hope, Steven; Chigogora, Sungano | 2020 | Epidemiology | 10.1097/EDE.0000000000001113 |
| Could Postnatal Womenâ€™s Groups Be Used to Improve Outcomes for Mothers and Children in High-Income Countries? A Systematic Review. | Sikorski, Catherine; Van Hees, Sietske; Lakhanpaul, Monica; Benton, Lorna; Martin, Jennifer; Costello, Anthony; Heys, Michelle | 2018 | Maternal & Child Health Journal | 10.1007/s10995-018-2606-y |
| COVID-19, Mental health and intervention-priorities among Northern Italian University students...14th European Public Health Conference (Virtual), Public health futures in a changing world, November 10-12, 2021. | Patrono, A.; Placidi, D.; Calza, S.; Brunelli, P.; Cagna, G.; Manco, A.; Renzetti, S.; Rota, M.; Moncada, S.; Lucchini, R. | 2021 | European Journal of Public Health |  |
| Cross-sectional and prospective relationships of passive and mentally active sedentary behaviours and physical activity with depression. | Hallgren, Mats; Nguyen, Thi-Thuy-Dung; Owen, Neville; Stubbs, Brendon; Vancampfort, Davy; Lundin, Andreas; Dunstan, David; Bellocco, Rino; Lagerros, Ylva Trolle | 2020 | British Journal of Psychiatry | 10.1192/bjp.2019.60 |
| Cross-sectional association of exercise, strengthening activities, and cardiorespiratory fitness on generalized anxiety, panic and depressive symptoms. | Loprinzi, Paul D.; Addoh, Ovuokerie; Wong Sarver, Nina; Espinoza, Ingrid; Mann, Joshua R. | 2017 | Postgraduate Medicine | 10.1080/00325481.2017.1336054 |
| Cross-sectional associations between long-term exposure to particulate matter and depression in China: The mediating effects of sunlight, physical activity, and neighborly reciprocity. | Wang, Ruoyu; Liu, Ye; Xue, Desheng; Yao, Yao; Liu, Penghua; Helbich, Marco | 2019 | Journal of Affective Disorders | 10.1016/j.jad.2019.02.007 |
| Cumulative Lifetime Marijuana Use and Incident Cardiovascular Disease in Middle Age: The Coronary Artery Risk Development in Young Adults (CARDIA) Study. | Reis, Jared P.; Auer, Reto; Bancks, Michael P.; Jr, David C. Goff; Lewis, Cora E.; Pletcher, Mark J.; Rana, Jamal S.; Shikany, James M.; Sidney, Stephen | 2017 | American Journal of Public Health | 10.2105/AJPH.2017.303654 |
| Cyberbulling as a new form of a threat: a physiological, psychological and medicinal aspects. | Mikhaylovsky, Mikhail N.; Lopatkova, Irina V.; Komarova, Nataliya M.; Rueva, Evgeniya O.; Tereschuk, Konstantin S.; Emelyanenkova, Anna V. | 2019 | Electronic Journal of General Medicine | 10.29333/ejgm/114268 |
| Depression and Its Comorbid Conditions More Serious in Women than in Men in the United States. | Kim, Woo Kyoung; Shin, Dayeon; Song, Won O. | 2015 | Journal of Women's Health (15409996) | 10.1089/jwh.2014.4919 |
| Depression, Quality of Life, Physical Activity, and the Impact of Drugs on Sexual Activity in a Population-Based Sample, Ages 20-59Â Years. | Steinke, Elaine E.; Mosack, Victoria; Hill, Twyla J. | 2018 | Issues in Mental Health Nursing | 10.1080/01612840.2017.1413463 |
| Depressive symptom trajectories and physical health: Persistence of problems from adolescence to young adulthood. | Ames, Megan E.; Leadbeater, Bonnie J. | 2018 | Journal of Affective Disorders | 10.1016/j.jad.2018.07.001 |
| Depressive Symptoms, Body Mass Index, and Physical Activity Self-Efficacy in African American Children. | Williams, Y'Esha V.; Cowan, Patricia A.; Graff, Joyce C. | 2020 | Journal of Child & Family Studies | 10.1007/s10826-020-01761-x |
| Designing and Validating a Questionnaire on Healthy Lifestyle to Reduce Depressive Symptoms among Adolescents. | Tajik, Esra; Abd Latiff, Latiffah; Chin Yit Siew; Awang, Hamidin; Adznam, Siti Nur'Asyura | 2020 | Iranian Journal of Psychiatry |  |
| Determinants of life satisfaction in Iranian children and adolescents: the CASPIANâ€IV study. | Kelishadi, Roya; Mirmoghtadaee, Parisa; Qorbani, Mostafa; Heshmat, Ramin; Motlagh, Mohammad Esmaeil; Magoul, Arman; Raeesi, Sina; Mansourian, Morteza; Gorabi, Armita Mahdavi; Safiri, Saeid | 2018 | Child & Adolescent Mental Health | 10.1111/camh.12239 |
| Developing webâ€based health guidance for coaches and parents in child athletics (track and field). | Jacobsson, Jenny; Ekberg, Joakim; Timpka, Toomas; Haggren RÃ¥sberg, Lena; SjÃ¶berg, Marina; Mirkovic, Dejan; Nilsson, Sverker | 2020 | Scandinavian Journal of Medicine & Science in Sports | 10.1111/sms.13661 |
| Diet and physical activity in the association between depression and metabolic syndrome: Constances study. | Matta, Joane; Hoertel, Nicolas; Kesse-Guyot, Emmanuelle; Plesz, Marie; Wiernik, Emmanuel; Carette, Claire; Czernichow, SÃ©bastien; Limosin, FrÃ©dÃ©ric; Goldberg, Marcel; Zins, Marie; Lemogne, CÃ©dric | 2019 | Journal of Affective Disorders | 10.1016/j.jad.2018.09.072 |
| Differential treatment effects of an integrated motivational interviewing and exercise intervention on depressive symptom profiles and associated factors: A randomised controlled cross-over trial among youth with major depression. | Nasstasia, Yasmina; Baker, Amanda L.; Lewin, Terry J.; Halpin, Sean A.; Hides, Leanne; Kelly, Brian J.; Callister, Robin | 2019 | Journal of Affective Disorders | 10.1016/j.jad.2019.08.035 |
| Digital Phenotyping With Mobile and Wearable Devices: Advanced Symptom Measurement in Child and Adolescent Depression. | Sequeira, Lydia; Battaglia, Marco; Perrotta, Steve; Merikangas, Kathleen; Strauss, John | 2019 | Journal of the American Academy of Child & Adolescent Psychiatry | 10.1016/j.jaac.2019.04.011 |
| Distinct health behavior and psychosocial profiles of young adult survivors of childhood cancers: a mixed methods study. | Lowe, Kincaid; Escoffery, Cam; Mertens, Ann; Berg, Carla; Mertens, Ann C; Berg, Carla J | 2016 | Journal of Cancer Survivorship | 10.1007/s11764-015-0508-1 |
| Do asthmatics benefit from music therapy? A systematic review. | Sliwka, Agnieszka; Wloch, Tomasz; Tynor, Dariusz; Nowobilski, Roman | 2014 | Complementary Therapies in Medicine | 10.1016/j.ctim.2014.07.002 |
| Do Injured Adolescent Athletes and Their Parents Agree on the Athletes' Level of Psychologic and Physical Functioning? | Oosterhoff, Jacobien H. F.; Bexkens, Rens; Vranceanu, Ana-Maria; Oh, Luke S. | 2018 | Clinical Orthopaedics & Related ResearchÂ® | 10.1007/s11999.0000000000000071 |
| Does exercise habit strength moderate the relationship between emotional distress and short-term memory in Malaysian primary school children? | Zainol, Nurul Ain; Hashim, Hairul Anuar | 2015 | Psychology, Health & Medicine | 10.1080/13548506.2014.955034 |
| Does manual therapy provide additional benefit to breathing retraining in the management of dysfunctional breathing? A randomised controlled trial. | Jones, Mandy; Troup, Fiona; Nugus, John; Roughton, Michael; Hodson, Margaret; Rayner, Charlotte; Bowen, Frances; Pryor, Jennifer | 2015 | Disability & Rehabilitation | 10.3109/09638288.2014.941020 |
| Does Physical Activity Moderate The Influence Of Sedentary Behavior On Health In Young People?...2021 ACSM Annual Meeting & World Congresses [Virtual], June 1 -5, 2021 | Schembri, Emanuel; Heinz, Andreas; Samuel, Robin | 2021 | Medicine & Science in Sports & Exercise | 10.1249/01.mss.0000761192.16245.9b |
| Domain-specific physical activity and sedentary behavior during pregnancy and postpartum depression risk in the French EDEN and ELFE cohorts. | van der Waerden, Judith; Nakamura, Aurelie; Pryor, Laura; Charles, Marie-Aline; El-Khoury, Fabienne; Dargent-Molina, Patricia | 2019 | Preventive Medicine | 10.1016/j.ypmed.2019.02.012 |
| Dropout from exercise randomized controlled trials among people with anxiety and stress-related disorders: A meta-analysis and meta-regression. | Vancampfort, Davy; SÃ¡nchez, Carlos Pelayo Ramos; Hallgren, Mats; Schuch, Felipe; Firth, Joseph; Rosenbaum, Simon; Van Damme, Tine; Stubbs, Brendon | 2021 | Journal of Affective Disorders | 10.1016/j.jad.2021.01.003 |
| Dyadic Effects of Individual and Friend on Physical Activity in College Students. | Kim, Gwang Suk; Lee, Chung Yul; Kim, In Sook; Lee, Tae Hwa; Cho, Eunhee; Lee, Hyeonkyeong; McCreary, Linda L.; Kim, Su Hee | 2015 | Public Health Nursing | 10.1111/phn.12176 |
| Eating Together and Health-Related Quality of Life Among Korean Adults. | Choi, Min-Jung; Park, Yong Gyu; Kim, Yang Hyun; Cho, Kyung Hwan; Nam, Ga Eun | 2020 | Journal of Nutrition Education & Behavior | 10.1016/j.jneb.2019.11.013 |
| Economic Hardship and Depression Among Women in Latino Farmworker Families. | Pulgar, Camila; Trejo, Grisel; Suerken, Cynthia; Ip, Edward; Arcury, Thomas; Quandt, Sara | 2016 | Journal of Immigrant & Minority Health | 10.1007/s10903-015-0229-6 |
| Effect of a relaxation training exercise on behaviour, anxiety, and pain during buccal infiltration anaesthesia in children: Randomized clinical trial. | Sridhar, Sowmya; Suprabha, Baranya Shrikrishna; Shenoy, Ramya; Shwetha, Kunnumal Thekkeveetil; Rao, Arathi | 2019 | International Journal of Paediatric Dentistry | 10.1111/ipd.12497 |
| Effect of Alternate Nostril Breathing Exercise on Experimentally Induced Anxiety in Healthy Volunteers Using the Simulated Public Speaking Model: A Randomized Controlled Pilot Study. | Kamath, Ashwin; Urval, Rathnakar P.; Shenoy, Ashok K. | 2017 | BioMed Research International | 10.1155/2017/2450670 |
| Effect of Diagnosed Sleep Disorders on Baseline Concussion Symptom, Cognitive, and Balance Assessments in Collegiate Athletes. | McAllister-Deitrick, Jamie; Trbovich, Alicia M.; Broglio, Steven P.; McCrea, Michael; McAllister, Thomas W.; Kontos, Anthony P. | 2020 | American Journal of Sports Medicine | 10.1177/0363546520902701 |
| Effect of Meridian Acupressure on Aerobic Performance of Healthy Young Population: A Randomized Controlled Study. | Ahmedov, Shahin; Filiz, Baris | 2018 | Journal of Alternative & Complementary Medicine | 10.1089/acm.2017.0089 |
| Effect of Social Anxiety on the Adoption of Robotic Training Partner. | Zhu, Dong Hong; Deng, Zhong Zhun | 2021 | CyberPsychology, Behavior & Social Networking | 10.1089/cyber.2020.0179 |
| Effect of tele-rehabilitation on glucose control, exercise capacity, physical fitness, muscle strength and psychosocial status in patients with type 2 diabetes: A double blind randomized controlled trial. | Duruturk, Neslihan; Ã–zkÃ¶slÃ¼, Manolya Acar | 2019 | Primary Care Diabetes | 10.1016/j.pcd.2019.03.007 |
| Effectiveness of Acceptance and Commitment Therapy in Increasing Resilience and Reducing Attrition of Injured US Navy Recruits. | Udell, Christopher J; Ruddy, Julie L; Procento, Philip M | 2018 | Military Medicine | 10.1093/milmed/usx109 |
| Effectiveness of an Exercise-Based Active Rehabilitation Intervention for Youth Who Are Slow to Recover After Concussion. | Gauvin-Lepage, JÃ©rÃ´me; Friedman, Debbie; Grilli, Lisa; Sufrategui, Maria; De Matteo, Carol; Iverson, Grant L.; Gagnon, Isabelle | 2020 | Clinical Journal of Sport Medicine | 10.1097/JSM.0000000000000634 |
| Effectiveness of Chinese Martial Arts and Philosophy to Reduce Reactive and Proactive Aggression in Schoolchildren. | Lai Chu Fung, Annis; Ka Hung Lee, Toney; Fung, Annis Lai Chu; Lee, Toney Ka Hung | 2018 | Journal of Developmental & Behavioral Pediatrics | 10.1097/DBP.0000000000000565 |
| Effectiveness of School-based Health Center Delivery of a Cognitive Skills Building Intervention in Young, Rural Adolescents: Potential Applications for Addiction and Mood. | Carr, Kayla L.; Stewart, Mary W. | 2019 | Journal of Pediatric Nursing | 10.1016/j.pedn.2019.04.013 |
| Effectiveness of Universal Self-regulationâ€“Based Interventions in Children and Adolescents: A Systematic Review and Meta-analysis. | Pandey, Anuja; Hale, Daniel; Das, Shikta; Goddings, Anne-Lise; Blakemore, Sarah-Jayne; Viner, Russell M. | 2018 | JAMA Pediatrics | 10.1001/jamapediatrics.2018.0232 |
| Effects of a self-managed home-based walking intervention on psychosocial health outcomes for breast cancer patients receiving chemotherapy: a randomised controlled trial. | Gokal, Kajal; Wallis, Deborah; Ahmed, Samreen; Boiangiu, Ion; Kancherla, Kiran; Munir, Fehmidah | 2016 | Supportive Care in Cancer | 10.1007/s00520-015-2884-5 |
| Effects of Acute Stretching on Cognitive Function and Mood States of Physically Inactive Young Adults. | Sudo, Mizuki; Ando, Soichi | 2020 | Perceptual & Motor Skills | 10.1177/0031512519888304 |
| Effects of Yoga on Heart Rate Variability and Depressive Symptoms in Women: A Randomized Controlled Trial. | Chu, I-Hua; Wu, Wen-Lan; Lin, I-Mei; Chang, Yu-Kai; Lin, Yuh-Jen; Yang, Pin-Chen | 2017 | Journal of Alternative & Complementary Medicine | 10.1089/acm.2016.0135 |
| Efficacy of Homeopathy in Addition to a Multidisciplinary Intervention for Overweight or Obesity in Mexican Adolescents: Study Protocol for a Randomized, Double-Blind, Placebo-Controlled Trial. | MacÃ­as-CortÃ©s, Emma; Arellano-Ãlvarez, SaraÃ­; Vega-Monroy, Sandra; Vera-PÃ©rez, Violeta; Llanes-GonzÃ¡lez, Lidia; SÃ¡nchez-Navarrete, Pilar; Enciso-GonzÃ¡lez, Dolores | 2020 | Homeopathy | 10.1055/s-0039-1697927 |
| ENERGY BALANCE AND VITAL SIGNS IN PEDIATRIC PATIENTS...Pediatric Research in Sports Medicine Society (PRiSM) 7th Annual Meeting, January 23-25, 2020, Glendale, Arizona. | Young, Julie A.; Napolitano, Jessica; Rauh, Mitchell J.; Nichols, Jeanne; Fischer, Anastasia N. | 2020 | Orthopaedic Journal of Sports Medicine | 10.1177/2325967120S00206 |
| Evaluating Multiple Domains of Health in High School Athletes With Sport-Related Concussion. | Williams, Richelle M.; Johnson, Rachel S.; Snyder Valier, Alison R.; Curtis Bay, R.; Valovich McLeod, Tamara C. | 2021 | Journal of Sport Rehabilitation | 10.1123/jsr.2019-0517 |
| Evaluating the Impact of an Anti-stigma Intervention on Pharmacy Students' Willingness to Counsel People Living with Mental Illness. | Bamgbade, Benita; Barner, Jamie; Ford, Kentya | 2017 | Community Mental Health Journal | 10.1007/s10597-016-0075-6 |
| Evaluation of an online cognitive behavioural therapy weight loss programme as an adjunct to anti-obesity medications and lifestyle interventions. | Tham, Marlene; Chong, Terence WH | 2020 | Australasian Psychiatry | 10.1177/1039856219871882 |
| Evaluation of Partner Violence in Female Patients with Fibromyalgia Syndrome...Congress of Clinical Child and Adult Psychiatry held in Istanbul on 23-25 September 2016. | Onur, Ozge Sahmelikoglu; Cesur, Ender; Donmezler, Fadime Gizem; Aydin, Filiz Yildiz; Vural, Meltem; Guru, Meltem | 2020 | Medical Journal of Bakirkoy | 10.5222/BMJ.2020.58076 |
| Evaluation of the DSM-5 Severity Specifier for Bulimia Nervosa in Treatment-Seeking Youth. | Dakanalis, Antonios; Colmegna, Fabrizia; Zanetti, Maria Assunta; Di Giacomo, Ester; Riva, Giuseppe; Clerici, Massimo | 2018 | Child Psychiatry & Human Development | 10.1007/s10578-017-0735-y |
| Evaluation of the effectiveness of relaxation in lowering the level of anxiety in young adults - a pilot study. | WILCZYÅƒSKA, DOMINIKA; ÅYSAK-RADOMSKA, ANNA; PODCZARSKA-GÅOWACKA, MAGDALENA; ZAJT, JOLANTA; DORNOWSKI, MARCIN; SKONIECZNY, PAWEÅ; WilczyÅ„ska, Dominika; Podczarska-GÅ‚owacka, Magdalena; Skonieczny, PaweÅ‚ | 2019 | International Journal of Occupational Medicine & Environmental Health | 10.13075/ijomeh.1896.01457 |
| "Even If They're Being Bad, Maybe They Need a Chance to Run Around": What Children Think About Recess. | Fink, Dale B.; Ramstetter, Catherine L. | 2018 | Journal of School Health | 10.1111/josh.12704 |
| Evidence that Swimming May Be Protective of Knee Osteoarthritis: Data from the Osteoarthritis Initiative. | Lo, Grace H.; Ikpeama, Uzoh E.; Driban, Jeffrey B.; Kriska, Andrea M.; McAlindon, Timothy E.; Petersen, Nancy J.; Storti, Kristi L.; Eaton, Charles B.; Hochberg, Marc C.; Jackson, Rebecca D.; Kwoh, C. Kent; Nevitt, Michael C.; Suarezâ€Almazor, Maria E.; Suarez-Almazor, Maria E | 2020 | PM & R: Journal of Injury, Function & Rehabilitation | 10.1002/pmrj.12267 |
| Examination of a board game approach to children's involvement in family-based weight management vs. traditional family-based behavioral counseling in primary care. | Sen, Merve; Uzuner, Arzu; Akman, Mehmet; Bahadir, Aliye Tugba; Borekci, Nazire Oncul; Viggiano, Emanuela | 2018 | European Journal of Pediatrics | 10.1007/s00431-018-3177-z |
| Examination of life quality, mental conditions and cognitive status of people over the age of 90: Results of a Hungarian local research. | Czibere, Ibolya; RÃ¡cz, Andrea; SzilvÃ¡si, Henrietta; Szikszai, Zita; Imre, SÃ¡ndor | 2019 | Central European Journal of Public Health | 10.21101/cejph.a4753 |
| Examining a training effect on the state anxiety response to an acute bout of exercise in low and high anxious individuals. | Lucibello, KM; Parker, J; Heisz, JJ; Lucibello, K M; Heisz, J J | 2019 | Journal of Affective Disorders | 10.1016/j.jad.2018.12.063 |
| Examining the Effects of Exercise on Pattern Separation and the Moderating Effects of Mood Symptoms. | Bernstein, Emily E.; McNally, Richard J. | 2019 | Behavior Therapy | 10.1016/j.beth.2018.09.007 |
| Excessive exercise among adolescents with eating disorders: examination of psychological and demographic variables. | Renz, Jessica A.; Fisher, Martin; Vidair, Hilary B.; Hirsch, Dina; Malizio, Joan; Barger, Hamutal; Fornari, Victor | 2019 | International Journal of Adolescent Medicine & Health | 10.1515/ijamh-2017-0032 |
| Exercise and Diet as Potential Moderators Between Trauma, Posttraumatic Stress, Depression, and Relationship Quality Among Emerging Adults. | Smith-Marek, Erika N.; Durtschi, Jared; Brown, Cameron; Dharnidharka, Prerana | 2016 | American Journal of Family Therapy | 10.1080/01926187.2016.1145080 |
| Exercise and mental health literacy in an Australian adult population. | Stanton, Robert; Rebar, Amanda; Rosenbaum, Simon | 2019 | Depression & Anxiety (1091-4269) | 10.1002/da.22851 |
| Exercise as an adjunct treatment for postpartum depression for women living in an inner cityâ€”A pilot study. | Forsyth, Jacky; Boath, Elizabeth; Henshaw, Carol; Brown, Hannah | 2017 | Health Care for Women International | 10.1080/07399332.2017.1295049 |
| Exercise as Medicine for Mental and Substance Use Disorders: A Meta-review of the Benefits for Neuropsychiatric and Cognitive Outcomes. | Ashdown-Franks, Garcia; Firth, Joseph; Carney, Rebekah; Carvalho, Andre F.; Hallgren, Mats; Koyanagi, Ai; Rosenbaum, Simon; Schuch, Felipe B.; Smith, Lee; Solmi, Marco; Vancampfort, Davy; Stubbs, Brendon | 2020 | Sports Medicine | 10.1007/s40279-019-01187-6 |
| EXERCISE DURING PREGNANCY: A Prescription for Improved Maternal/Fetal Well-being. | Davenport, Margie H. | 2020 | ACSM's Health & Fitness Journal | 10.1249/FIT.0000000000000602 |
| Exercise for Depression: A Feasibility Trial Exploring Neural Mechanisms. | Gujral, Swathi; Aizenstein, Howard; Reynolds III, Charles F.; Butters, Meryl A.; Grove, George; Karp, Jordan F.; Erickson, Kirk I.; Reynolds, Charles F 3rd | 2019 | American Journal of Geriatric Psychiatry | 10.1016/j.jagp.2019.01.012 |
| Exercise for PTSD in Women Veterans: A Proof-of-Concept Study. | Shivakumar, Geetha; Anderson, Elizabeth H.; SurÃ­s, Alina M.; North, Carol S. | 2017 | Military Medicine | 10.7205/MILMED-D-16-00440 |
| Experimentally investigating the joint effects of physical activity and sedentary behavior on depression and anxiety: A randomized controlled trial. | Blough, Jeremiah; Loprinzi, Paul D. | 2018 | Journal of Affective Disorders | 10.1016/j.jad.2018.07.019 |
| Exploring patients' attitudes to different intervention approaches for supporting psychosocial needs. | Taylor, Francesca; Hare, Jennifer; Combes, Gill | 2016 | Journal of Renal Care | 10.1111/jorc.12182 |
| Exploring the differentiated relationship between appearance and fitnessâ€related social anxiety and the risk of eating disorders and depression in young adults. | Alcarazâ€IbÃ¡Ã±ez, Manuel; Sicilia, Ãlvaro; Paterna, Adrian | 2019 | Scandinavian Journal of Psychology | 10.1111/sjop.12584 |
| Facilitators and Barriers of Sleep in Young Adults With Type 1 Diabetes. | Griggs, Stephanie; Whittemore, Robin; Redeker, Nancy S.; Grey, Margaret | 2020 | Diabetes Educator | 10.1177/0145721720916179 |
| Factors Associated with Migraine in the General Population of Spain: Results from the European Health Survey 2014. | Roy, RubÃ©n; SÃ¡nchez-RodrÃ­guez, Elisabet; GalÃ¡n, Santiago; Racine, MÃ©lanie; Castarlenas, Elena; Jensen, Mark P; MirÃ³, Jordi | 2019 | Pain Medicine | 10.1093/pm/pny093 |
| Factors Associated with Suicidal Thought and Help-Seeking Behaviour in Transition-Aged Youth versus Adults. | MacKinnon, Nathalie; Colman, Ian | 2016 | Canadian Journal of Psychiatry | 10.1177/0706743716667417 |
| Facts and ideas from anywhere. | Roberts, William C. | 2019 | Baylor University Medical Center Proceedings | 10.1080/08998280.2019.1583692 |
| Fatigue in young people with Duchenne muscular dystrophy. | Elâ€Aloul, Basmah; Speechley, Kathy N; Wei, Yi; Wilk, Piotr; Campbell, Craig; El-Aloul, Basmah | 2020 | Developmental Medicine & Child Neurology | 10.1111/dmcn.14248 |
| Fatigue-Related Cognitive-Behavioral Factors in Survivors of Childhood Cancer: Comparison with Chronic Fatigue Syndrome and Survivors of Adult-Onset Cancer. | van Deuren, Sylvia; van Dulmen-den Broeder, Eline; Boonstra, Amilie; Gielissen, Marieke; Blijlevens, Nicole; Loonen, Jacqueline; Knoop, Hans | 2021 | Journal of Adolescent & Young Adult Oncology | 10.1089/jayao.2020.0094 |
| Feasibility, acceptability, and effectiveness of a multidisciplinary intervention in childhood obesity from primary care: Nutrition, physical activity, emotional regulation, and family. | SepÃºlveda, Ana R.; Solano, Santos; Blanco, Miriam; Lacruz, Tatiana; Veiga, Oscar | 2020 | European Eating Disorders Review | 10.1002/erv.2702 |
| Females and males do not differ for fatigability, muscle damage and magnitude of the repeated bout effect following maximal eccentric contractions. | Bruce, Christina D.; Ruggiero, Luca; Dix, Gabriel U.; Cotton, Paul D.; McNeil, Chris J. | 2021 | Applied Physiology, Nutrition & Metabolism | 10.1139/apnm-2020-0516 |
| Frequency of Breakfast and Physical Fitness among Chinese College Students. | Yufei Cui; Wenbo Zhang; Qiang Gong; Yanbo Chen; Shulei Chen; Ziqiang Wu | 2018 | American Journal of Health Behavior | 10.5993/AJHB.42.1.15 |
| Functional Resistance Training and Affective Response in Female College-Age Students. | FARO, JAMIE; WRIGHT, JULIE A.; HAYMAN, LAURA L.; HASTIE, MARISA; GONA, PHILIMON N.; WHITELEY, JESSICA A. | 2019 | Medicine & Science in Sports & Exercise | 10.1249/MSS.0000000000001895 |
| Gender differences in irritable bowel syndrome among medical students at Inner Mongolia Medical University, China: a cross-sectional study. | Wang, Ying; Jin, Feng; Chi, Baofeng; Duan, Shengyun; Zhang, Qing; Liu, Ying; Hao, Wenli; Sun, Juan | 2016 | Psychology, Health & Medicine | 10.1080/13548506.2016.1144890 |
| GENDER PREFERENCES OF YOUTH ATHLETES FOR THEIR SPORTS MEDICINE PROVIDERS: A SYSTEMATIC REVIEW...Pediatric Research in Sports Medicine (PRiSM), 8th Annual Meeting, 28-30 January, 2021. | Chung, Jane S.; Merkel, Donna; Carter, Cordelia W.; Kraus, Emily; Rizzone, Katherine | 2021 | Orthopaedic Journal of Sports Medicine | 10.1177/2325967121S00108 |
| Gender-specific prevalence and associated factors of major depressive disorder and generalized anxiety disorder in a Chinese rural population: the Henan rural cohort study. | Luo, Zhicheng; Li, Yuqian; Hou, Yitan; Liu, Xiaotian; Jiang, Jingjing; Wang, Yan; Liu, Xue; Qiao, Dou; Dong, Xiaokang; Li, Ruiying; Wang, Fang; Wang, Chongjian | 2019 | BMC Public Health | 10.1186/s12889-019-8086-1 |
| German recommendations for physical activity and physical activity promotion in adults with noncommunicable diseases. | Geidl, Wolfgang; Abu-Omar, Karim; Weege, Mayra; Messing, Sven; Pfeifer, Klaus | 2020 | International Journal of Behavioral Nutrition & Physical Activity | 10.1186/s12966-020-0919-x |
| Globus Sensation And Retching In A 21-year-old Male Football Player.: 1322...American College of Sports Medicine, Annual Meeting and World Congresses, May 31-June 4, 2022, San Diego, California | Donohoe, Brian; Goldman, Joshua; Polster, Douglas | 2022 | Medicine & Science in Sports & Exercise | 10.1249/01.mss.0000879160.87669.31 |
| Characteristics Associated with Successful Weight Management in Youth with Obesity. | Gorecki, Michelle C.; Feinglass, Joseph M.; Binns, Helen J. | 2019 | Journal of Pediatrics | 10.1016/j.jpeds.2019.05.039 |
| Childhood overweight and obesity and the risk of depression across the lifespan. | Gibson-Smith, Deborah; Halldorsson, Thorhallur I.; Bot, Mariska; Brouwer, Ingeborg A.; Visser, Marjolein; Thorsdottir, Inga; Birgisdottir, Bryndis E.; Gudnason, Vilmundur; Eiriksdottir, Gudny; Launer, Lenore J.; Harris, Tamara B.; Gunnarsdottir, Ingibjorg | 2020 | BMC Pediatrics | 10.1186/s12887-020-1930-8 |
| Children's Physical Activity and Depression: A Meta-analysis. | Korczak, Daphne J.; Madigan, Sheri; Colasanto, Marlena | 2017 | Pediatrics | 10.1542/peds.2016-2266 |
| Child's Number of Activities as a Moderator of Depressive Symptoms. | Ryan, Paige; Kaskas, Maysa; Davis, Thompson; NoackLeSage, Franziska; Lilly, Megan; Castagna, Peter; Shaheen, Georgia | 2017 | Journal of Child & Family Studies | 10.1007/s10826-017-0853-y |
| Cigarette- and snus-modified association between unprotected exposure to noise from hunting rifle caliber weapons and high frequency hearing loss. A cross-sectional study among swedish hunters. | Honeth, Louise; StrÃ¶m, Peter; Ploner, Alexander; Bagger-SjÃ¶bÃ¤ck, Dan; Rosenhall, Ulf; NyrÃ©n, Olof | 2016 | Noise & Health | 10.4103/1463-1741.195796 |
| CLINICAL PREDICTORS OF SYMPTOM RESOLUTION FOR CHILDREN AND ADOLESCENTS WITH SPORT-RELATED CONCUSSION...PRiSM 6th Annual Meeting, January 17-19, 2019, Atlanta, Georgia. | Howell, David R.; Potter, Morgan N.; Kirkwood, Michael W.; Wilson, Pamela E.; Provance, Aaron; Wilson, Julie C. |  | Orthopaedic Journal of Sports Medicine | 10.1177/2325967119S00007 |
| Clinical Reaction-Time Performance Factors in Healthy Collegiate Athletes. | Caccese, Jaclyn B.; Eckner, James T.; Franco-MacKendrick, Lea; Hazzard, Joseph B.; Meng Ni; Broglio, Steven P.; McAllister, Thomas W.; McCrea, Michael; Buckley, Thomas A. | 2020 | Journal of Athletic Training (Allen Press) | 10.4085/1062-6050-164-19 |
| Clustering of lifestyle factors and the relationship with depressive symptoms among adolescents in Northeastern China. | Cao, Ruilin; Gao, Tingting; Hu, Yueyang; Qin, Zeying; Ren, Hui; Liang, Leilei; Li, Chuanen; Mei, Songli | 2020 | Journal of Affective Disorders | 10.1016/j.jad.2020.05.064 |
| Combating the Dangers of Sedentary Activity on Child and Adolescent Mental Health During the Time of COVID-19. | Mittal, Vijay A.; Firth, Joseph; Kimhy, David | 2020 | Journal of the American Academy of Child & Adolescent Psychiatry | 10.1016/j.jaac.2020.08.003 |
| Community-Based Preventive Interventions for Depression and Anxiety in Women. | Maleki, Fatemeh Mollarahimi; Massahikhaleghi, Parissa; Tehrani-Banihashemi, Arash; Davoudi, Farnoush; Nojomi, Marzieh | 2020 | Archives of Iranian Medicine (AIM) |  |
| Comorbid Depression and Obesity: Correlates and Synergistic Association With Noncommunicable Diseases Among Australian Men. | Tilahun Nigatu Haregu; Tayu Lee, John; Oldenburg, Brian; Armstrong, Gregory; Haregu, Tilahun Nigatu; Lee, John Tayu | 2020 | Preventing Chronic Disease | 10.5888/pcd17.190420 |
| Comparison of the effect of educational software and booklet on anxiety and pain during labour: a randomised controlled clinical trial. | Abbasi, Parastoo; Mohammad-Alizadeh Charandabi, Sakineh; Mirghafourvand, Mojgan | 2021 | Journal of Obstetrics & Gynaecology | 10.1080/01443615.2020.1736017 |
| Conceptions of Adolescent Friendship Quality in Sport and Music Domains. | Phillips Reichter, Alison; Weiss, Maureen R. | 2019 | Research Quarterly for Exercise & Sport | 10.1080/02701367.2019.1632412 |
| Consistent participation in organized physical activity predicts emotional adjustment in children. | BriÃ¨re, FrÃ©dÃ©ric N.; Imbeault, Arianne; Goldfield, Gary S.; Pagani, Linda S. | 2020 | Pediatric Research | 10.1038/s41390-019-0417-5 |
| Correction to: Is There an Association Between Diet, Physical Activity and Depressive Symptoms in the Perinatal Period? An Analysis of the UPBEAT Cohort of Obese Pregnant Women...Wilson CA, Seed P, Flynn AC, et al. Is There an Association Between Diet, Ph | Wilson, Claire A.; Seed, Paul; Flynn, Angela C.; Howard, Louise M.; Molyneaux, Emma; Sigurdardottir, Julie; Poston, Lucilla | 2020 | Maternal & Child Health Journal | 10.1007/s10995-020-02957-9 |
| Correlates of short sleep duration among adolescents. | Widome, Rachel; Berger, Aaron T.; Lenk, Kathleen M.; Erickson, Darin J.; Laska, Melissa N.; Iber, Conrad; Kilian, Gudrun; Wahlstrom, Kyla | 2019 | Journal of Adolescence | 10.1016/j.adolescence.2019.10.011 |
| Correlation of PROMIS Physical Function, Pain Interference, and Depression in Pediatric and Adolescent Patients in the Ambulatory Sports Medicine Clinic. | Makhni, Eric C.; Meldau, Jason E.; Blanchett, Jacob; Borowsky, Peter; Stephens, Jeffrey; Muh, Stephanie; Moutzouros, Vasilios | 2019 | Orthopaedic Journal of Sports Medicine | 10.1177/2325967119851100 |
| Could greater physical activity reduce population prevalence and socio-economic inequalities in children's mental health problems? A policy simulation. | Sungano Chigogora; Pearce, Anna; Law, Catherine; Viner, Russell; Chittleborough, Catherine; Griffiths, Lucy J.; Hope, Steven; Chigogora, Sungano | 2020 | Epidemiology | 10.1097/EDE.0000000000001113 |
| Could Postnatal Womenâ€™s Groups Be Used to Improve Outcomes for Mothers and Children in High-Income Countries? A Systematic Review. | Sikorski, Catherine; Van Hees, Sietske; Lakhanpaul, Monica; Benton, Lorna; Martin, Jennifer; Costello, Anthony; Heys, Michelle | 2018 | Maternal & Child Health Journal | 10.1007/s10995-018-2606-y |
| COVID-19, Mental health and intervention-priorities among Northern Italian University students...14th European Public Health Conference (Virtual), Public health futures in a changing world, November 10-12, 2021. | Patrono, A.; Placidi, D.; Calza, S.; Brunelli, P.; Cagna, G.; Manco, A.; Renzetti, S.; Rota, M.; Moncada, S.; Lucchini, R. | 2021 | European Journal of Public Health |  |
| Cross-sectional and prospective relationships of passive and mentally active sedentary behaviours and physical activity with depression. | Hallgren, Mats; Nguyen, Thi-Thuy-Dung; Owen, Neville; Stubbs, Brendon; Vancampfort, Davy; Lundin, Andreas; Dunstan, David; Bellocco, Rino; Lagerros, Ylva Trolle | 2020 | British Journal of Psychiatry | 10.1192/bjp.2019.60 |
| Cross-sectional association of exercise, strengthening activities, and cardiorespiratory fitness on generalized anxiety, panic and depressive symptoms. | Loprinzi, Paul D.; Addoh, Ovuokerie; Wong Sarver, Nina; Espinoza, Ingrid; Mann, Joshua R. | 2017 | Postgraduate Medicine | 10.1080/00325481.2017.1336054 |
| Cross-sectional associations between long-term exposure to particulate matter and depression in China: The mediating effects of sunlight, physical activity, and neighborly reciprocity. | Wang, Ruoyu; Liu, Ye; Xue, Desheng; Yao, Yao; Liu, Penghua; Helbich, Marco | 2019 | Journal of Affective Disorders | 10.1016/j.jad.2019.02.007 |
| Cumulative Lifetime Marijuana Use and Incident Cardiovascular Disease in Middle Age: The Coronary Artery Risk Development in Young Adults (CARDIA) Study. | Reis, Jared P.; Auer, Reto; Bancks, Michael P.; Jr, David C. Goff; Lewis, Cora E.; Pletcher, Mark J.; Rana, Jamal S.; Shikany, James M.; Sidney, Stephen | 2017 | American Journal of Public Health | 10.2105/AJPH.2017.303654 |
| Cyberbulling as a new form of a threat: a physiological, psychological and medicinal aspects. | Mikhaylovsky, Mikhail N.; Lopatkova, Irina V.; Komarova, Nataliya M.; Rueva, Evgeniya O.; Tereschuk, Konstantin S.; Emelyanenkova, Anna V. | 2019 | Electronic Journal of General Medicine | 10.29333/ejgm/114268 |
| Depression and Its Comorbid Conditions More Serious in Women than in Men in the United States. | Kim, Woo Kyoung; Shin, Dayeon; Song, Won O. | 2015 | Journal of Women's Health (15409996) | 10.1089/jwh.2014.4919 |
| Depression, Quality of Life, Physical Activity, and the Impact of Drugs on Sexual Activity in a Population-Based Sample, Ages 20-59Â Years. | Steinke, Elaine E.; Mosack, Victoria; Hill, Twyla J. | 2018 | Issues in Mental Health Nursing | 10.1080/01612840.2017.1413463 |
| Depressive symptom trajectories and physical health: Persistence of problems from adolescence to young adulthood. | Ames, Megan E.; Leadbeater, Bonnie J. | 2018 | Journal of Affective Disorders | 10.1016/j.jad.2018.07.001 |
| Depressive Symptoms, Body Mass Index, and Physical Activity Self-Efficacy in African American Children. | Williams, Y'Esha V.; Cowan, Patricia A.; Graff, Joyce C. | 2020 | Journal of Child & Family Studies | 10.1007/s10826-020-01761-x |
| Designing and Validating a Questionnaire on Healthy Lifestyle to Reduce Depressive Symptoms among Adolescents. | Tajik, Esra; Abd Latiff, Latiffah; Chin Yit Siew; Awang, Hamidin; Adznam, Siti Nur'Asyura | 2020 | Iranian Journal of Psychiatry |  |
| Determinants of life satisfaction in Iranian children and adolescents: the CASPIANâ€IV study. | Kelishadi, Roya; Mirmoghtadaee, Parisa; Qorbani, Mostafa; Heshmat, Ramin; Motlagh, Mohammad Esmaeil; Magoul, Arman; Raeesi, Sina; Mansourian, Morteza; Gorabi, Armita Mahdavi; Safiri, Saeid | 2018 | Child & Adolescent Mental Health | 10.1111/camh.12239 |
| Developing webâ€based health guidance for coaches and parents in child athletics (track and field). | Jacobsson, Jenny; Ekberg, Joakim; Timpka, Toomas; Haggren RÃ¥sberg, Lena; SjÃ¶berg, Marina; Mirkovic, Dejan; Nilsson, Sverker | 2020 | Scandinavian Journal of Medicine & Science in Sports | 10.1111/sms.13661 |
| Diet and physical activity in the association between depression and metabolic syndrome: Constances study. | Matta, Joane; Hoertel, Nicolas; Kesse-Guyot, Emmanuelle; Plesz, Marie; Wiernik, Emmanuel; Carette, Claire; Czernichow, SÃ©bastien; Limosin, FrÃ©dÃ©ric; Goldberg, Marcel; Zins, Marie; Lemogne, CÃ©dric | 2019 | Journal of Affective Disorders | 10.1016/j.jad.2018.09.072 |
| Differential treatment effects of an integrated motivational interviewing and exercise intervention on depressive symptom profiles and associated factors: A randomised controlled cross-over trial among youth with major depression. | Nasstasia, Yasmina; Baker, Amanda L.; Lewin, Terry J.; Halpin, Sean A.; Hides, Leanne; Kelly, Brian J.; Callister, Robin | 2019 | Journal of Affective Disorders | 10.1016/j.jad.2019.08.035 |
| Digital Phenotyping With Mobile and Wearable Devices: Advanced Symptom Measurement in Child and Adolescent Depression. | Sequeira, Lydia; Battaglia, Marco; Perrotta, Steve; Merikangas, Kathleen; Strauss, John | 2019 | Journal of the American Academy of Child & Adolescent Psychiatry | 10.1016/j.jaac.2019.04.011 |
| Distinct health behavior and psychosocial profiles of young adult survivors of childhood cancers: a mixed methods study. | Lowe, Kincaid; Escoffery, Cam; Mertens, Ann; Berg, Carla; Mertens, Ann C; Berg, Carla J | 2016 | Journal of Cancer Survivorship | 10.1007/s11764-015-0508-1 |
| Do asthmatics benefit from music therapy? A systematic review. | Sliwka, Agnieszka; Wloch, Tomasz; Tynor, Dariusz; Nowobilski, Roman | 2014 | Complementary Therapies in Medicine | 10.1016/j.ctim.2014.07.002 |
| Do Injured Adolescent Athletes and Their Parents Agree on the Athletes' Level of Psychologic and Physical Functioning? | Oosterhoff, Jacobien H. F.; Bexkens, Rens; Vranceanu, Ana-Maria; Oh, Luke S. | 2018 | Clinical Orthopaedics & Related ResearchÂ® | 10.1007/s11999.0000000000000071 |
| Does exercise habit strength moderate the relationship between emotional distress and short-term memory in Malaysian primary school children? | Zainol, Nurul Ain; Hashim, Hairul Anuar | 2015 | Psychology, Health & Medicine | 10.1080/13548506.2014.955034 |
| Does manual therapy provide additional benefit to breathing retraining in the management of dysfunctional breathing? A randomised controlled trial. | Jones, Mandy; Troup, Fiona; Nugus, John; Roughton, Michael; Hodson, Margaret; Rayner, Charlotte; Bowen, Frances; Pryor, Jennifer | 2015 | Disability & Rehabilitation | 10.3109/09638288.2014.941020 |
| Does Physical Activity Moderate The Influence Of Sedentary Behavior On Health In Young People?...2021 ACSM Annual Meeting & World Congresses [Virtual], June 1 -5, 2021 | Schembri, Emanuel; Heinz, Andreas; Samuel, Robin | 2021 | Medicine & Science in Sports & Exercise | 10.1249/01.mss.0000761192.16245.9b |
| Domain-specific physical activity and sedentary behavior during pregnancy and postpartum depression risk in the French EDEN and ELFE cohorts. | van der Waerden, Judith; Nakamura, Aurelie; Pryor, Laura; Charles, Marie-Aline; El-Khoury, Fabienne; Dargent-Molina, Patricia | 2019 | Preventive Medicine | 10.1016/j.ypmed.2019.02.012 |
| Dropout from exercise randomized controlled trials among people with anxiety and stress-related disorders: A meta-analysis and meta-regression. | Vancampfort, Davy; SÃ¡nchez, Carlos Pelayo Ramos; Hallgren, Mats; Schuch, Felipe; Firth, Joseph; Rosenbaum, Simon; Van Damme, Tine; Stubbs, Brendon | 2021 | Journal of Affective Disorders | 10.1016/j.jad.2021.01.003 |
| Dyadic Effects of Individual and Friend on Physical Activity in College Students. | Kim, Gwang Suk; Lee, Chung Yul; Kim, In Sook; Lee, Tae Hwa; Cho, Eunhee; Lee, Hyeonkyeong; McCreary, Linda L.; Kim, Su Hee | 2015 | Public Health Nursing | 10.1111/phn.12176 |
| Eating Together and Health-Related Quality of Life Among Korean Adults. | Choi, Min-Jung; Park, Yong Gyu; Kim, Yang Hyun; Cho, Kyung Hwan; Nam, Ga Eun | 2020 | Journal of Nutrition Education & Behavior | 10.1016/j.jneb.2019.11.013 |
| Economic Hardship and Depression Among Women in Latino Farmworker Families. | Pulgar, Camila; Trejo, Grisel; Suerken, Cynthia; Ip, Edward; Arcury, Thomas; Quandt, Sara | 2016 | Journal of Immigrant & Minority Health | 10.1007/s10903-015-0229-6 |
| Effect of a relaxation training exercise on behaviour, anxiety, and pain during buccal infiltration anaesthesia in children: Randomized clinical trial. | Sridhar, Sowmya; Suprabha, Baranya Shrikrishna; Shenoy, Ramya; Shwetha, Kunnumal Thekkeveetil; Rao, Arathi | 2019 | International Journal of Paediatric Dentistry | 10.1111/ipd.12497 |
| Effect of Alternate Nostril Breathing Exercise on Experimentally Induced Anxiety in Healthy Volunteers Using the Simulated Public Speaking Model: A Randomized Controlled Pilot Study. | Kamath, Ashwin; Urval, Rathnakar P.; Shenoy, Ashok K. | 2017 | BioMed Research International | 10.1155/2017/2450670 |
