# Supplementary material for: A systematic review of sport-based adolescent mental health awareness programmes
Source: PLoS One. 2025 Mar 27;20(3):e0315315. doi: 10.1371/journal.pone.0315315 (PMC11949344; doi:10.1371/journal.pone.0315315)
Supplement: S5 File — (DOCX) [file pone.0315315.s005.docx]

S5: Data Extracted from Included Studies

| **Author, Year of Publication, Intervention** | **Study design; duration** | **Psychological Behaviour Change Theory** | **Participant Sample characteristics** | **Outcome measures and measurement tools** | **Main Findings** |
| --- | --- | --- | --- | --- | --- |
| Liddle et al. 2021  [38]  Ahead of the Game | Clustered RCT; One 45-minute session | Integrated Behaviour Change Theory  Self-Determination Theory | 102 males (mean age 14.30)  Intervention=59  Control= 63 | *Intentions to Provide Help-* Unvalidated measure created with participants identifying how likely they are to engage in a list of 6 behaviours.  *Depression and Anxiety Literacy-* The Depression Literacy Questionnaire [60] and the Anxiety Literacy Questionnaire [61]. 13 of the 22 items for each measure were used as remaining items capture knowledge of effective treatments that were not included in the HOAM content.  *Intentions to Seek Help*- General Help Seeking Questionnaire [54]  *Confidence to Provide Help-* Single item question asking how confident they are to help someone [55]  *Mental Health Literacy-* Two subscales of the Mental Health Literacy Scale [53] | -Statistically significant interaction effect (p<0.01) in intentions to provide help post attendance at the workshop, however this was not sustained 4 weeks post workshop.  -Significant interaction effect for depression and anxiety literacy for the intervention group vs. control group, time point one verse time point two (p<0.01).  -Effects for depression literacy were washed out at follow-up, but were sustained for anxiety literacy (p<0.01).  -Increased intentions to seek help from informal services at follow-up by both groups (this may be because of conversations between friends or siblings).  -Confidence to provide help was higher pre workshop, suggestions possible ceiling effects.  -Significant interaction effect on decreasing stigmatizing attitudes at all three time points and against age as a covariate (p>0.01). |
| McKenzie et al. 2021  [39]  Waves of Wellness | Mixed-methods Exploratory Research; 8 weeks of 2-hour sessions (45 minutes mental health related discussion, 60 minutes sea-surfing) | Ecological Dynamics Perspective | 9 youth (mean age 14.9)  8 females  1 male | *Brief resiliency scale*- Participants reported how quickly they would bounce back or recover from stress on six items using a one (strongly disagree) to five (strongly agree) scale. [58]  *Semi structured interviews* | -Improvements in resilience post intervention, however, not maintained at follow up.  -Key overarching theme that came out of the interviews was noted as ‘a shared experience’. This is split into two lower order themes; a unique learning environment and personal growth. |
| Moore et al. 2021  [40]  Wellbeing Warriors | Experimental research design using a clustered RCT; 10 weeks, one 50/60-minute session per week | Not reported | Schools= 5; 283 youth (mean age 12.76)  Intervention= 142  Control= 141 | *Child and youth resilience measure -* 28 items to test total resilience which was the primary outcome [59] | -Statistically significant effects in favour of the intervention group vs control from baseline to post- intervention (p<0.01). |
| Patafio et al. 2021  [41]  Read the Play | Repeated measures experimental-control design; single 1 hour session | Not reported | Sport clubs= 12, 330 youth (mean age 13.73)  Experimental= 10 sport clubs (272)  Control= 2 sport clubs (58) | *Mental health literacy*- Mental Health Literacy Scale [53] with modifications  *Help-seeking intentions*- General Help-Seeking Questionnaire [54]. 11 help sources included plus space to specify help source not listed in options provided.  *Help-seeking behaviours*- Actual Help-Seeking Questionnaire (adapted from [56]) | -Significant intervention effects mental health literacy, for the low-scoring youth cohort (p=0.03).  -Statistically significant intervention effects recorded for help-seeking intentions (p<0.03), and specifically for informal (p<0.02) and sport-related help-seeking intentions (p<0.01), all by the low scoring cohort.  -Reports of actual help-seeking did not significantly change, with the exception of the low scoring cohort (p=0.01).  -Qualitative assessment found there was reduced stigma associated with mental health difficulties.  -Normalised mental health experiences.  -Facilitated deeper understandings of mental health and acquisition of coping strategies. |
| Vella et al. 2021  [15]  Ahead of the Game | Non-randomised control trial; 2 45-minute face-to-face sessions and 6 internet modules lasting 15 minutes each) | Socio-Ecological Approach | 816 males  Intervention= 350 males (mean age 14.53)  Control= 466 males (mean age 14.66) | *Depression and anxiety literacy*- 13 items of the Depression Literacy Questionnaire [60] and 13 items of the Anxiety Literacy Questionnaire [61]  *Resilience*- 10 item version of the Connor-Davidson Resilience Scale [57]  *Confidence to seek-help-* Single item from the Mental Health Literacy Scale [53]  *Help-seeking intentions*- The General Help Seeking Questionnaire [54]  *Stigmatizing attitudes*- The youth version of the Social Distance Scale (adapted) [62] | -Significant improvements in depression and anxiety literacy for the intervention group, with group-time interaction effect of p<0.01.  -Significant improvements in resilience with an interaction effect of p<0.01.  -Significant interaction effect for confidence to seek help (p=<0.03).  -Significant group-time interaction effect in intentions to seek help from formal sources (p<0.01).  -No significant effects for stigmatizing attitudes. |
| Wynters et al. 2021  [42]  Ahead of the Game | Retrospective qualitative design; 45 minutes in person session | Not reported | 33 males, 6 focus groups | No outcome measures quantitatively assessed | Key themes that came up in conversation were:  -Increased confidence to provide or seek help.  -Addressing mental health stigma remains a challenge. |
