# Supplementary material for: A systematic review of sport-based adolescent mental health awareness programmes
Source: PLoS One. 2025 Mar 27;20(3):e0315315. doi: 10.1371/journal.pone.0315315 (PMC11949344; doi:10.1371/journal.pone.0315315)
Supplement: S6 File — (DOCX) [file pone.0315315.s006.docx]

S6: Methodological Quality Assessments

**Table 3: Downs and Black (1998) methodological quality assessment**

|  |  | **Study** | | | |
| --- | --- | --- | --- | --- | --- |
| **Domain** | **Items** | Liddle et al. (2021)  [38] | Moore et al. (2021)  [40] | Patafio et al. (2021)  [41] | Vella et al. (2021)  [15] |
| **Reporting** | 1 | 1 | 1 | 1 | 1 |
|  | 2 | 1 | 1 | 1 | 1 |
|  | 3 | 1 | 1 | 1 | 1 |
|  | 4 | 1 | 1 | 1 | 1 |
|  | 5 | 0 | 0 | 0 | 0 |
|  | 6 | 1 | 1 | 1 | 1 |
|  | 7 | 1 | 1 | 1 | 1 |
|  | 8 | 0 | 0 | 0 | 1 |
|  | 9 | 1 | 1 | 1 | 1 |
|  | 10 | 1 | 1 | 1 | 1 |
| **External Validity** | 11 | 1 | 1 | 1 | 1 |
|  | 12 | 1 | 1 | 1 | 1 |
|  | 13 | 1 | 1 | 1 | 1 |
| **Internal Validity- bias** | 14 | 1 | 0 | 0 | 0 |
|  | 15 | 0 | 0 | 0 | 0 |
|  | 16 | 1 | 1 | 1 | 1 |
|  | 17 | 1 | 1 | 0 | 1 |
|  | 18 | 1 | 1 | 1 | 1 |
|  | 19 | 1 | 0 | 0 | 1 |
|  | 20 | 1 | 1 | 1 | 1 |
| **Internal Validity- confounding** | 21 | 1 | 0 | 0 | 0 |
|  | 22 | 1 | 1 | 0 | 1 |
|  | 23 | 1 | 1 | 0 | 0 |
|  | 24 | 1 | 1 | 0 | 0 |
|  | 25 | 0 | 0 | 0 | 1 |
|  | 26 | 1 | 1 | 1 | 1 |
| **Power** | 27 | 0 | 1 | 0 | 1 |
| **Total Score (out of a possible 28)** |  | 22 | 20 | 15 | 21 |
| **Quality** |  | Excellent | Moderate | Moderate | Excellent |

**Table 4: Critical appraisal skills programme (2018)**

| **Study:** | **Wynters et al., (2021)** [42] |
| --- | --- |
| **Question** | **Answer** |
| 1: Was there a clear statement of the aims of the research? | Yes |
| 2: Is a qualitative methodology appropriate? | Yes |
| 3: Was the research design appropriate to address the aims of the research? | Yes |
| 4: Was the recruitment strategy appropriate to the aims of the research? | Yes |
| 5: Was the data collected in a way that addressed the research issue? | Yes |
| 6: Has the relationship between researcher and participants been adequately considered? | Yes |
| 7: Have ethical issues been taken into consideration? | Yes |
| 8: Was the data analysis sufficiently rigorous? | Yes |
| 9: Is there a clear statement of findings? | Yes |
| 10: How valuable is the research? | Valuable |

**Table 5: Mixed methods appraisal tool (2018)**

| **Study:** | **McKenzie et al., (2021)** [39] |
| --- | --- |
| **Question** | **Answer** |
| 5.1. Is there an adequate rationale for using a mixed methods design to address the research question? | Yes |
| 5.2. Are the different components of the study effectively integrated to answer the research question? | Yes |
| 5.3. Are the outputs of the integration of qualitative and quantitative components adequately interpreted? | No |
| 5.4. Are divergences and inconsistencies between quantitative and qualitative results adequately addressed? | No |
| 5.5. Do the different components of the study adhere to the quality criteria of each tradition of the methods involved? | Yes |
